# Organofunctionalized borotungstate polyoxometalates as tunable photocatalysts for oxidative dimerization of amines†

**DOI:** 10.1039/d4sc03534h

**Published:** 2024-08-12

**Authors:** Nicole Tsang, Alexander J. Kibler, Stephen P. Argent, Hon Wai Lam, Kieran D. Jones, Graham N. Newton

**Affiliations:** a The GSK Carbon Neutral Laboratories for Sustainable Chemistry, University of Nottingham, Jubilee Campus Triumph Road Nottingham NG7 2TU UK hon.lam@nottingham.ac.uk kieran.jones@nottingham.ac.uk graham.newton@nottingham.ac.uk; b School of Chemistry, University of Nottingham University Park NG7 2RD UK

## Abstract

Organofunctionalized borotungstate Keggin polyoxometalates, (^*n*^Bu_4_N)_3_H[HBW_11_O_39_(P(O)Ph)_2_] (PBW_11_), (^*n*^Bu_4_N)_3_H[HBW_11_O_39_(As(O)Ph)_2_] (AsBW_11_), and (^*n*^Bu_4_N)_4_[HBW_11_O_39_(PhSiOSiPh)] (SiBW_11_), were synthesized and structurally characterized. Cyclic voltammetry showed that the electronic properties of the clusters are dependent on the nature of the appended main group atoms (P, As, or Si). The first reduction potentials were found to shift positively with respect to that of the unmodified parent species (^*n*^Bu_4_N)_5_[BW_12_O_40_], with PBW_11_ showing the largest shift at +100 mV. All clusters were evaluated as photocatalysts for the oxidative dimerization of amines where the organophosphonate hybrid PBW_11_ was found to be the most active. This study demonstrates how organofunctionalization of polyoxometalates may be used to tune and improve their performance as photocatalysts for organic reactions.

## Abstract 2

The organic hybridization of borotungstate Keggin polyoxometalates is shown to modulate the electronic structure of the clusters, allowing the rational design of single-electron transfer photocatalysts that can be cycled under oxygen.

## Graphical abstract

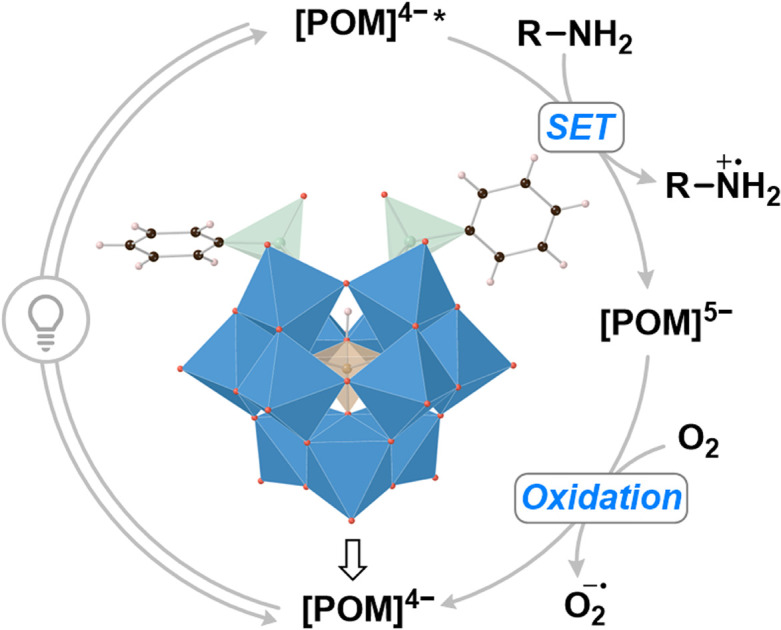

## 1 Introduction

Polyoxometalates (POMs) are anionic metal oxide clusters with tunable photo- and electrochemical properties.^1^ Typically, they can be reversibly interconverted between multiple redox states, allowing them to function as effective electron and proton reservoirs. This versatility renders POMs promising materials for applications such as energy storage,^2–5^ materials science,^6,7^ and catalysis.^8–10^ They have been shown to facilitate a range of organic reactions, including oxidations and reductions,^11^ hydrolysis,^12^ and Lewis acid processes,^13,14^ acting as both electrocatalysts^15,16^ and photocatalysts.^17,18^ The most prominent example of a POM photocatalyst in the literature is tetrabutylammonium decatungstate (TBADT), (^*n*^Bu_4_N)_4_[W_10_O_32_], an isopolyoxometalate that has proven to be efficient for hydrogen atom transfer (HAT) chemistry.^19,20^ While other POM catalysts have been reported to operate through single electron transfer (SET)^21^ and proton-coupled electron transfer (PCET) reaction pathways,^22^ none have matched the reaction diversity and performance of TBADT.

In contrast to isopolyoxometalates such as TBADT, in which the cluster anion comprises only metal ions and oxide ligands, heteropolyoxometalates contain templating anions (*i.e.* PO_4_^3−^, SiO_4_^4−^, or BO_4_^5−^) that stabilize the clusters to the extent that metal vacancies can be introduced and lacunary POMs isolated. These metal vacancy sites on monolacunary polyoxotungstates can be filled by main group elements such as P^V^, As^V^, Si^IV^, and Sn^IV^, which can act as tethers to an effectively unlimited range of organic moieties yielding hybrid organic–inorganic POMs.^23–25^ The electrochemical and photochemical properties of these hybrid POMs depend on the nature of the metal addenda ions (commonly W or Mo), the templating anion, the counter-cations, the main group linker atoms, and the appended organic groups, which gives the synthetic chemist a plethora of options when designing functional POMs.^26^

**Fig. 1:**
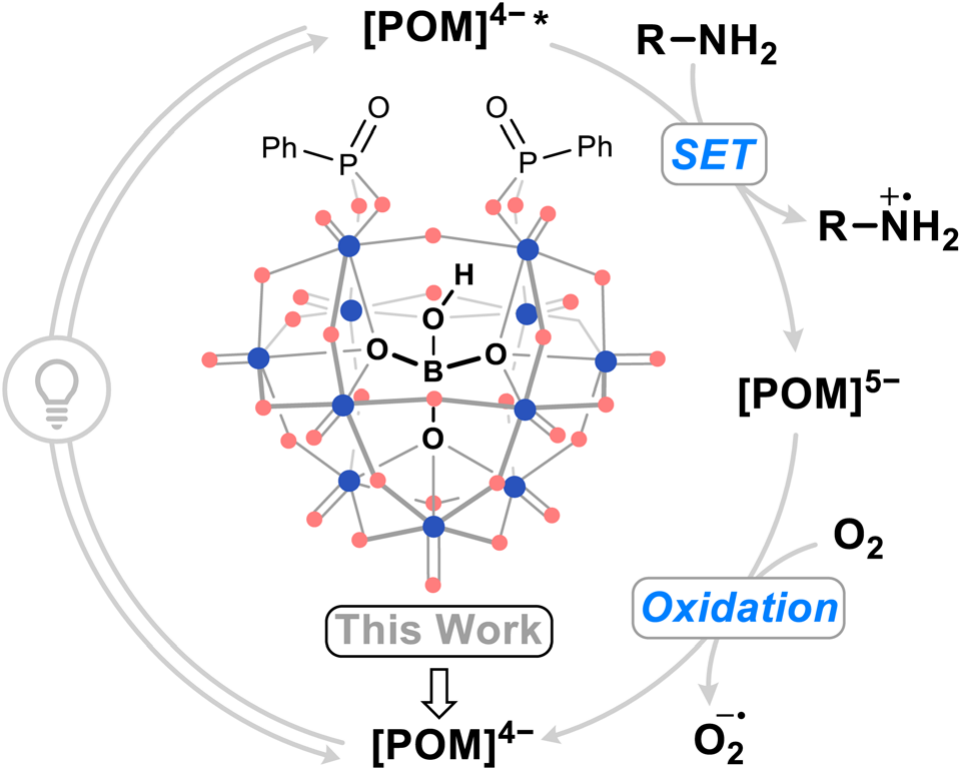
Summarized photocatalytic cycle of organic–inorganic hybrid borotungstate POM-mediated single electron transfer (SET) with amines. Blue dots = W^VI^, red dots = oxygen.

The modification of heteropolyoxometalate structures, often by transition metal substitution,^27–30^ has been shown to improve POM performance as SET photocatalysts. The oxidation of amines by a photoexcited POM photocatalyst to yield aminium radical cations (Fig. 1), versatile reactive intermediates used in amination chemistry,^31^ followed by regeneration of the catalyst by an oxidant has served as a benchmarking reaction for these POM photocatalyts. While organofunctionalization has proven effective in enhancing the visible light activity of heteropolyoxometalates compared to their non-hybridized plenary anion counterparts,^32–36^ this strategy has yet to be demonstrated towards improved photocatalysis for organic synthesis. Hybridization with organophosphonates results in visible light active Wells–Dawson phosphotungstate photocatalysts for the photodegradation of the pollutant indigo dye,^35^ however, the stability of their reduced state means that these hybrid POMs are oxidized only slowly in air. Overall, this makes for an inefficient catalytic cycle, impacting their broader applicability as photocatalysis for organic synthesis. We hypothesized that modulation of the frontier orbital energies of POMs through organofunctionalization could lead to improved catalytic turnover, preferably using molecular oxygen as a sustainable oxidant, and application of the systems in organic reactions. Considering the limitations of existing phosphotungstate systems, hybrid POMs derived from underexplored heteropolytungstates may yield promising candidates for an aerobic-SET photocatalytic cycle.

To the best of our knowledge, only three examples of organofunctionalized borotungstates, all bearing organo(thio)phosphonate groups, are known in literature.^37–39^ These reports are inconsistent in their cluster charge assignments, and no electrochemical properties have been described. Herein, we report the synthesis and characterization of three organofunctionalized Keggin borotungstate hybrid POMs, namely (^*n*^Bu_4_N)_3_H[HBW_11_O_39_(P(O)Ph)_2_] (PBW_11_), (^*n*^Bu_4_N)_3_H[HBW_11_O_39_(As(O)Ph)_2_] (AsBW_11_), and (^*n*^Bu_4_N)_4_[HBW_11_O_39_(PhSiOSiPh)] (SiBW_11_). Their structural characterization and electronic properties are discussed, with particular emphasis on the impact of hybridization on promoting photocatalytic behavior.

## 2 Results and discussion

### 2.1 Synthesis and characterization of hybrid borotungstates

**Fig. 2:**
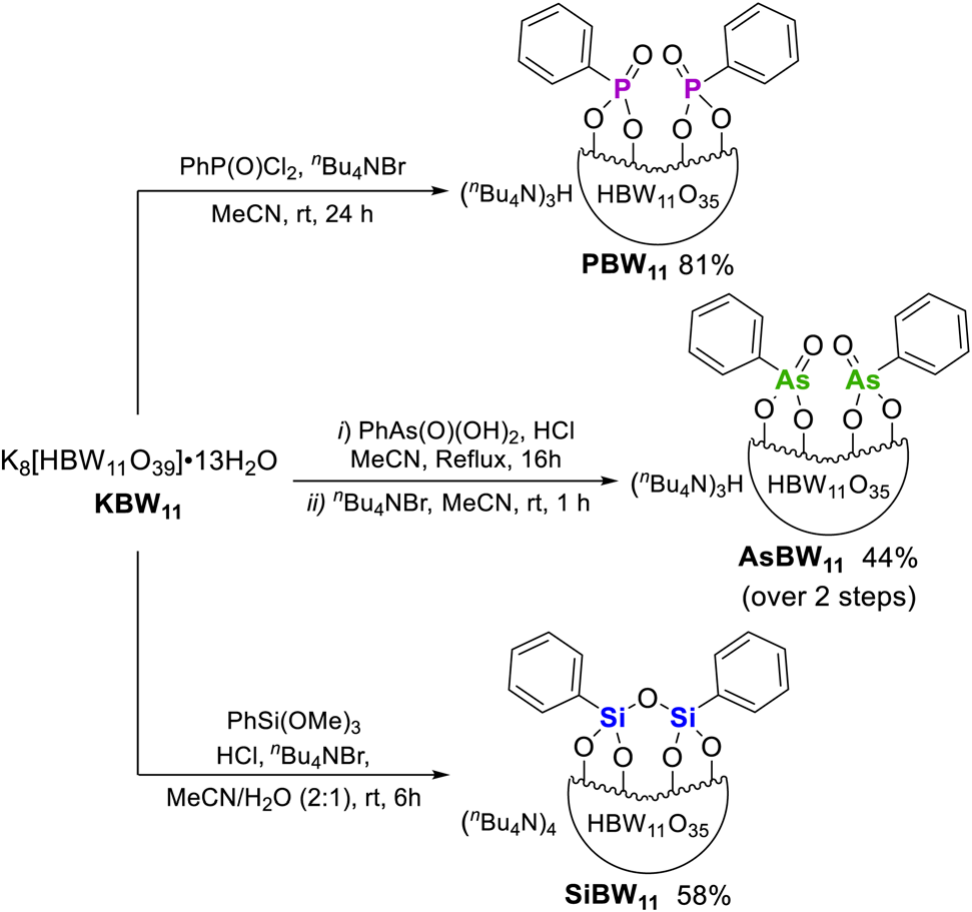
Synthesis of organic–inorganic hybrid borotungstates PBW_11_, AsBW_11_, and SiBW_11_ from the lacunary borotungstate.

Hybrid POMs (^*n*^Bu_4_N)_3_H[HBW_11_O_39_(P(O)Ph)_2_] (PBW_11_), (^*n*^Bu_4_N)_3_H[HBW_11_O_39_(As(O)Ph)_2_] (AsBW_11_), and (^*n*^Bu_4_N)_4_[HBW_11_O_39_(PhSiOSiPh)] (SiBW_11_) were prepared by hybridization of the lacunary K_8_[HBW_11_O_39_] (KBW_11_) with phenylphosphonic dichloride, phenylarsonic acid, or trimethoxy(phenyl)silane, respectively (Fig. 2). Hybrid POMs PBW_11_, AsBW_11_, and SiBW_11_ were characterized by NMR, UV-Vis and IR spectroscopies and electrospray ionization mass spectrometry (see ESI† for details). These hybrid POMs exhibited an anionic charge of 4− instead of the expected 5− due to monoprotonation of the templating anion. This proton was observed as a distinctive singlet peak in the range 4.5–4.9 ppm in the ^1^H NMR spectra (400 MHz, CD_3_CN), corresponding to a B–OH̲ peak. By ^11^B NMR spectroscopy, the hybrid borotungstates B̲O_4_H is observed as a characteristic broad singlet (2.04–2.13 ppm, 128 MHz, CD_3_CN), due to the quadrupolar effect.^40,41^ Protonation of the templating anion is not observed in the analogous hybrid silicotungstate [SiW_11_O_39_(P(O)Ph)_2_]^4−^ or phosphotungstate [PW_11_O_39_(P(O)Ph)_2_]^3−^ and may be ascribed to the comparatively smaller size and greater charge density of the central boron atom.^42^

**Fig. 3:**
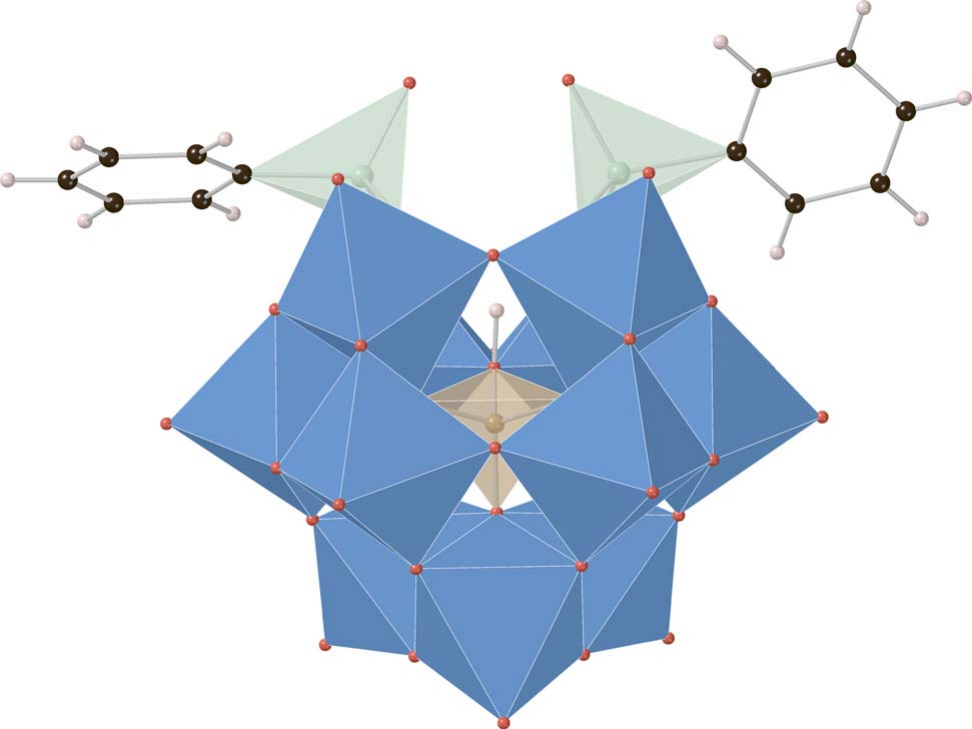
X-ray crystal structure of K_3_H[HBW_11_O_39_(As(O)Ph)_2_] with disordered elements, solvent, and potassium cations omitted for clarity. Color code: blue = W, red = O, black = carbon, green = As, orange = B, pink = H.

Crystals of K_3_H[HBW_11_O_39_(P(O)Ph)_2_] and K_3_H[HBW_11_O_39_(As(O)Ph)_2_] (Fig. 3) suitable for single crystal X-ray diffraction were prepared by the vapor diffusion method with MeCN and *tert*-butyl methyl ether (anti-solvent). The structures solve in monoclinic space groups *P*2_1_ and *P*2_1_/*n*, respectively (see ESI† for details). In both cases, three potassium cations per POM anion cluster were identified, supporting the assigned elemental compositions, and two phenylpnictogen groups were covalently bound to these clusters in a 2 : 1 ratio in a manner analogous to previously reported phenylpnictogen hybrid polyoxotungstates.^23,35,37,43^ Crystallographic support for the assignment and location of the templating boronate hydrogen atom for K_3_H[HBW_11_O_39_(As(O)Ph)_2_] was made by a hydrogen omit map calculated for all of the data which indicated an electron density peak of 0.49 e^−^ Å^−3^ in the refined location of the hydrogen atom B–OH̲ (see ESI† for details). This marks the first crystallographic report supporting the assigned protonated hybrid borotungstate structure.

The hydrolytic stability of a hybrid POM may be a useful indicator of the stability and suitability of the cluster as a photocatalyst, particularly under protic conditions. Previous studies have shown aryl phosphonate hybridized phospho-and silico-tungstate POMs to undergo hydrolytic cleavage of the phosphoryl group from the lacunary site within a period of 24 h.^35,43,44^ To date, the hydrolytic stability of hybrid borotungstates has not been investigated, and therefore compounds PBW_11_, AsBW_11_, and SiBW_11_ were dissolved in DMSO-d_6_ : D_2_O (9 : 1) and monitored over time using ^1^H, ^31^P, and ^11^B NMR spectroscopies (Fig. S9–15†). The hydrolytic stability of organophosphonate hybrid PBW_11_ is similar to that reported for the isoionic silicotungstate PSiW_11_,^43^ with hydrolyzed phenyl phosphonate (12.97 ppm) accumulating slowly over a 24 h period as indicated by ^31^P NMR spectroscopy (162 MHz, 298 K). The starting material PBW_11_ (20.42 ppm) was still present as a major component even after 7 days. Conversely, organoarsonate hybrid AsBW_11_ was observed to undergo immediate hydrolysis upon the addition of D_2_O, evident through the disappearance of BOH̲ peak at 4.81 ppm and change in appearance of the aromatic region in the ^1^H NMR spectrum (400 MHz, 298 K). By ^11^B NMR spectroscopy (128 MHz, 298 K), the absence of the broad singlet at 1.83 ppm, indicative of AsBW_11_, and presence of a sharp singlet at 1.76 ppm suggests that following cleavage of the phenyl arsonate groups, the metastable lacunary [HBW_11_O_39_]^8−^ reacts further to give the favored plenary Keggin species [BW_12_O_40_]^5−^. Finally, the organosilyl POM SiBW_11_ was found to have excellent hydrolytic stability over a month, with no apparent change in the aromatic region of the ^1^H NMR spectrum (400 MHz, 298 K) and unchanged ^11^B NMR spectroscopic (128 MHz, 298 K) data, suggesting little to no cleavage of the phenylsiloxane unit. Notably, the relative integration of the BOH̲ peak at 4.72 ppm in the ^1^H NMR spectrum decreased by *ca.* 65% over the first week; proton–deuterium exchange (B–OH/D) is proposed to have occurred without affecting the hybrid POM structure.

### 2.2 Electronic characterization

**Fig. 4:**
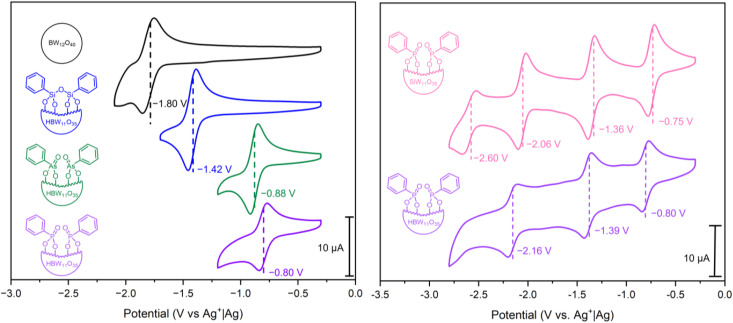
(Left) CV of 1 mM of compounds BW_12_, PBW_11_, AsBW_11_, and SiBW_11_ in anhydrous DMF with 0.1 M ^*n*^Bu_4_NPF_6_ supporting electrolyte. (Right) CV of 1 mM of compounds PBW_11_ and PSiW_11_ in anhydrous DMF with 0.1 M ^*n*^Bu_4_NPF_6_ supporting electrolyte. All CVs were recorded at 100 mV s^−1^ using a glassy carbon working electrode (*d* = 3 mm and *A* = 0.071 cm^2^), Pt wire counter electrode and a AgNO_3_|Ag non-aqueous reference electrode. The standard potentials are highlighted for the redox processes.

Cyclic voltammetry (CV) was performed in DMF [glassy carbon (*d* = 3 mm) working electrode, Pt wire counter electrode, and Ag^+^|Ag reference electrode, in 0.1 M ^*n*^Bu_4_NPF_6_ supporting electrolyte] to explore the redox properties of hybrid POMs PBW_11_, AsBW_11_, and SiBW_11_, with comparison to the parent plenary POM (^*n*^Bu_4_N)_5_[BW_12_O_40_] (BW_12_) (Fig. 4, left). In all cases, quasi-reversible redox couples corresponding to one-electron W^VI^/W^V^ processes were observed. The first reduction potentials of the hybrid POMs PBW_11_, AsBW_11_, and SiBW_11_ were shifted positively (by between 0.4 V and 1 V) compared to that of the unmodified BW_12_, attributed to the difference in cluster charge.

Organophosphonate PBW_11_ exhibits the most positive first redox process (*E*_1/2_ = −0.80 V *vs.* Ag^+^|Ag), followed by the organoarsonate hybrid AsBW_11_ (*E*_1/2_ = −0.88 V *vs.* Ag^+^|Ag), and then the organosilyl hybrid SiBW_11_ (*E*_1/2_ = −1.42 V *vs.* Ag^+^|Ag). This trend in redox potential can be ascribed to the electron-withdrawing nature of the phosphonate and arsonate groups compared to the silyl anchoring group.^23,45^ Furthermore, the 80 mV difference between the phosphonate PBW_11_ and arsonate AsBW_11_ is likely due to the variance in bond lengths and hence differing frontier orbital overlap. A similar observation was made in analysis of the previously reported phenylpnictogen hybrid Wells–Dawson phosphotungstate variants.^23^

As an extension of this study, the previously reported organofunctionalized silicotungstate (^*n*^Bu_4_N)_3_H[SiW_11_O_39_(P(O)Ph)_2_] (PSiW_11_) was synthesized and electronically characterized (see ESI† for details).^43^ Given the unique protonation of the central borate anion and subsequent charge of borotungstate hybrid POMs (4−), the silicotungstate hybrid can be described as an isoionic analogue of hybrid PBW_11_, with the only difference being the central templating anion. The CV of PSiW_11_ displayed four quasi-reversible redox processes (Fig. 4, right), with the first redox process being shifted +50 mV (*E*_1/2_ = −0.75 V *vs.* Ag^+^|Ag) compared to its isoionic analogue.^42^ Beyond the first redox potential, the voltammetry of PSiW_11_ and PBW_11_ share a similar voltametric profile with the next two reversible processes all falling within 100 mV of the corresponding processes in the voltammogram. Based on the relative reduction potentials, PSiW_11_ is the most readily reduced POM in the series. This is consistent with it having the lowest LUMO energy.

In the absorption spectra, a hyperchromic effect was observed for hybrids PBW_11_, AsBW_11_, and SiBW_11_ compared to the parent borotungstate POM BW_12_. A similar absorption profile was also observed for silicotungstate hybrid PSiW_11_. The borotungstate hybrids exhibit a higher absorbance in the UV region relative to the plenary POM, while hybrid SiBW_11_ has a lower absorbance relative to the other hybrids (Fig. S8†).

### 2.3 Photocatalytic oxidative dimerization of amines

**Fig. 5:**
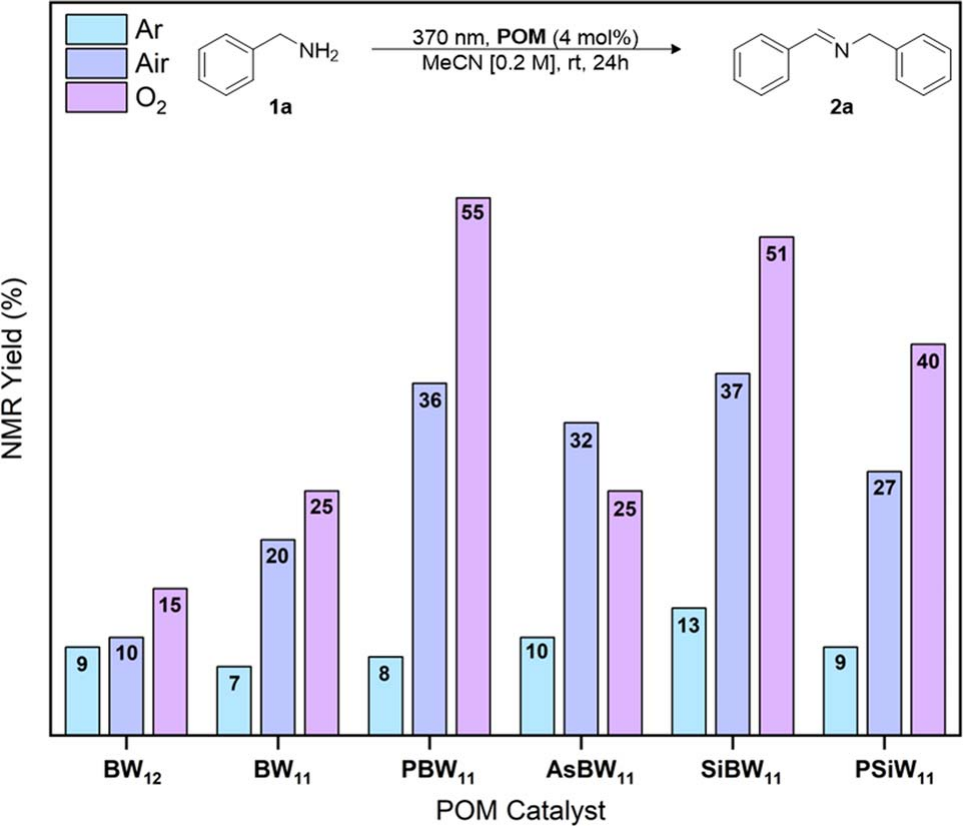
POM-mediated photooxidative dimerization of benzyl amine under anaerobic or aerobic conditions. NMR yield determined by ^1^H NMR spectroscopic analysis using 1,3-benzodioxole as an internal standard.

The oxidative dimerization of amines serves as an effective reaction to benchmark the single electron transfer (SET) photocatalytic activity (Fig. 1) of our new borotungstate hybrid POMs (Table S6†).^27,29,30,46,47^ The plenary BW_12_, lacunary (^*n*^Bu_4_N)_8_[HBW_11_O_39_] (BW_11_), hybrid PBW_11_, AsBW_11_, SiBW_11_ borotungstates, as well as the isoionic silicotungstate PSiW_11_ were tested as SET photocatalysts for the oxidative dimerization of benzyl amine. A solution of the amine 1a and POM (4 mol%) in MeCN was irradiated at 370 nm for 24 h (Fig. 5). The NMR yield of the target imine 2a was determined by ^1^H NMR spectroscopy, with 1,3-benzodioxole as the internal standard. Under aerobic conditions, the plenary (BW_12_) and lacunary (BW_11_) Keggin borotungstates gave imine 2a in low yields, 10% and 20% respectively. Under the same conditions, hybrids PBW_11_, AsBW_11_, and SiBW_11_ gave yields between 32% and 37%, indicating that organofunctionalization of Keggin borotungstates improves catalysis in this reaction. In comparison, the TBADT-catalyzed reaction gave the desired imine in 5% yield, accompanied by a multitude of side products attributed to competitive hydrogen atom transfer at the benzylic position and subsequent radical reactions with oxygen and imines leading to a series of hydroperoxides, amides, and various oligomers derived from 1a and 2a.^19,20,48,49^

Conducting the reaction in an anaerobic environment led to reduced yields for all POMs suggesting molecular oxygen is essential for catalytic turnover. With this in mind, we performed the same reactions under an oxygen atmosphere and observed a substantial increase in yield of the imine 2a when using PBW_11_ or SiBW_11_ (55% and 51%, respectively). The isoionic analogue PSiW_11_ followed a similar trend to the borotungstate PBW_11_, where an increase of yield from 27% to 40% was achieved with change of atmosphere (aerobic to O_2_, respectively). In comparison, these conditions did not improve the yield of 2a when AsBW_11_ was used, marking AsBW_11_ as the poorest performing catalyst of the hybrid POMs investigated, likely resulting from its limited hydrolytic and electrochemical stability (Fig. S6, S12 and 13†).

**Fig. 6:**
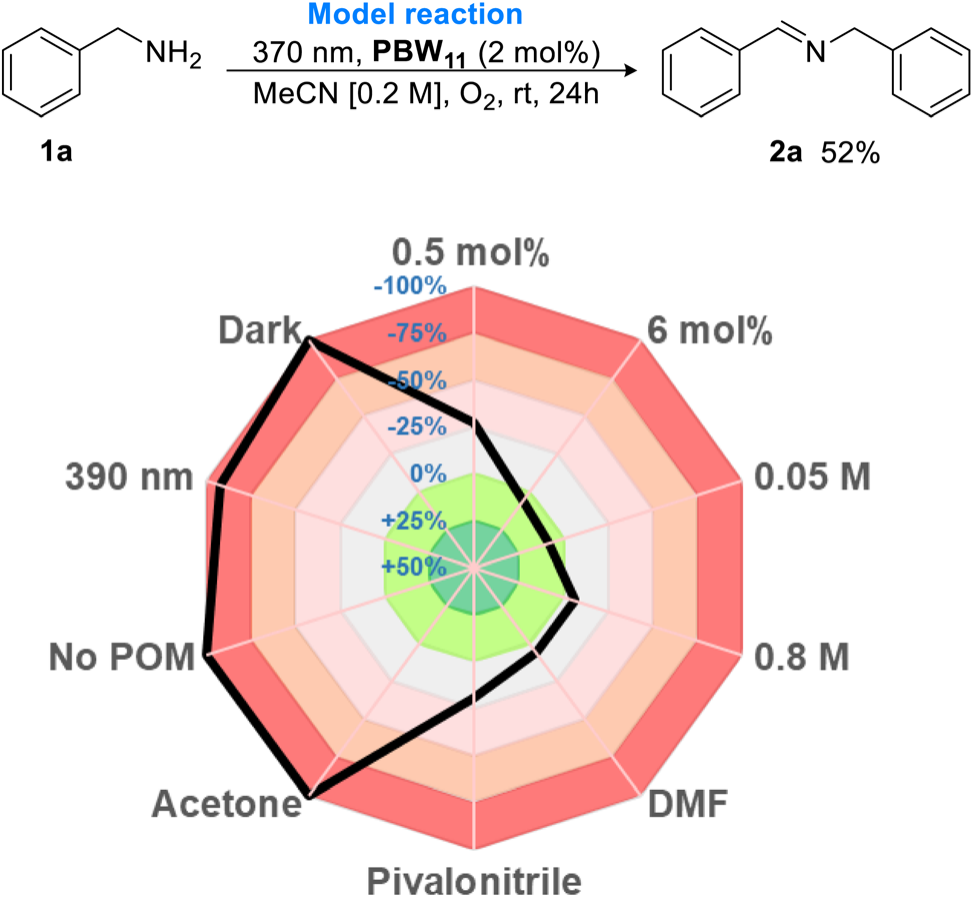
Optimization of PBW_11_ photocatalyzed oxidative dimerization of benzyl amine illustrated as a color-coded radar diagram. Yields determined by ^1^H NMR analysis using 1,3-benzodioxole as an internal standard and presented as a percentage deviation from the model reaction.

Based on these results, phenylphosphonate hybrid borotungstate PBW_11_ is the lead candidate as a SET photocatalyst for oxidative amine dimerization. The reaction parameters were further assessed using the Glorius robustness model and represented as a radar graph (Fig. 6).^50^ The percentage deviation in yield from the model reaction was determined, where negative integers represent a decrease in NMR yield, and positive integers signify an increase in NMR yield (Table S5†). In the absence of POM or light, no reaction occurred. The use of a longer wavelength of light (390 nm) was ineffective, as expected from the absorption spectrum of PBW_11_ (Fig. S8†). A 2 mol% catalyst loading was optimum, with a lower loading (0.5 mol%) resulting in a drop in yield (38%, −31% deviation from model), while increased loading did not significantly improve the yield. The reaction was robust towards concentration changes, and we therefore opted for a higher concentration of 0.4 M to reduce solvent waste. Changing the solvent from MeCN to pivalonitrile, DMF, or acetone was unfavorable, especially in the case of acetone which completely shut down reactivity. Finally, the optimal conditions were determined to be 24 h irradiation (370 nm – 43 W Kessil LED light) of benzylamine with POM catalyst PBW_11_ (2 mol%), dissolved in MeCN [0.4 M], under an oxygen atmosphere, which gave the desired imine product 2a in a moderate yield of 57%.

**Fig. 7:**
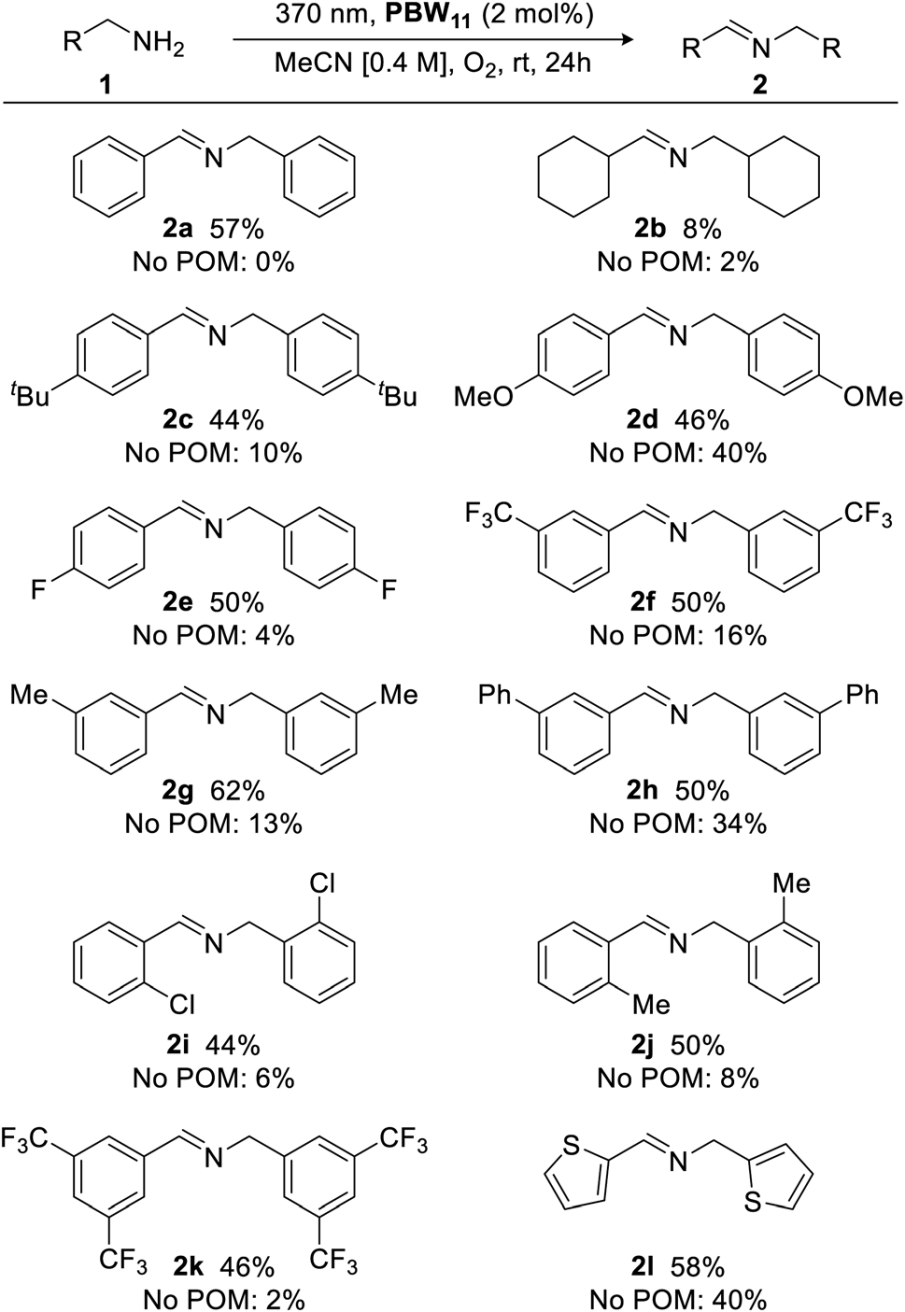
Photocatalytic oxidative dimerization of various amines. Yields determined by ^1^H NMR analysis using 1,3-benzodioxole as an internal standard.

With optimized conditions in hand, the scope of the borotungstate photocatalytic oxidative dimerization reaction of various amines was explored (Fig. 7). Cyclohexylmethylamine gave imine 2b in 8% yield, indicating the importance of an activated methylene unit for acceptable yields. Thus, a series of *para*-, *meta*-, and *ortho*-substituted benzyl amines bearing alkyl, methoxy, phenyl, as well as halogen-containing groups were evaluated and these gave the desired imines in moderate yields with little variance (44–62%), irrespective of substituent effects. Largely, these reactions proceeded cleanly with reasonable starting material recovery being observed for 2a, 2b, 2e–i and 2k (45%, 47%, 49%, 37%, 17%, 11%, 51%, 54%, respectively). Given the use of an energetic UVA light source with an atmosphere of oxygen, catalyst-free reactions were also conducted to assess radical-initiated autoxidation reaction pathways for each substrate.^51,52^ Generally, without the POM catalyst, oxidative dimerization of primary benzylic amines was insignificant (yields <10%), highlighting the efficacy of PBW_11_. However, anisole-, biphenyl-, and thiophene-bearing amines yielded secondary aldimines 2d, 2h, and 2l, respectively, in low to moderate yields (34–40%). Overall, PBW_11_ was determined to be a consistently effective photocatalyst for the oxidative dimerization of activated methylamines, albeit, giving moderate yields.

## 3 Conclusions

We have described the synthesis of three organofunctionalized Keggin borotungstate POMs bearing phenyl-substituted phosphonate (PBW_11_), arsonate (AsBW_11_) or siloxane (SiBW_11_) groups, each of which were structurally and electronically characterized, as well as investigated as photocatalysts for the oxidative dimerization of amines. *p*-Block organofunctionalization was found to dramatically influence the fundamental properties of borotungstate POMs with the phenylphosphonate hybrid determined to be the most effective catalyst in the series. PBW_11_ also outperformed the isoionic silicotungstate analogue PSiW_11_, indicating the importance of both the templating anion and linker group when considering hybrid heteropolyoxometalates as functional materials. Overall, this study has led to the development of the first generation of covalently modified organic–inorganic hybrid POMs capable of turning over photocatalytic single electron transfer chemistry for organic synthesis, a significant step forward in the field of hybrid POMs. It is also particularly interesting to note the improvement in catalytic performance of the organofunctionalized systems compared to the established photo-oxidation catalyst tetrabutylammonium decatungstate. The design strategy described in this study illustrates how POM-based catalysts can be tuned and optimized to drive specific organic reactions.

## Author contributions

GNN, KDJ and HWL conceptualized and supervised the project, contributed to data analysis and co-wrote the manuscript. NT performed experimental work, helped to develop the catalysis methodology and helped to write the manuscript. AJK contributed additional data analysis and experimental support. SPA managed the collection and analysis of single crystal data.

## Conflicts of interest

There are no conflicts to declare.

## Supplementary Material

SC-015-D4SC03534H-s001

SC-015-D4SC03534H-s002

## Data Availability

The authors confirm that the data supporting the findings of this study are available within the article and its supplementary materials.
