# Supplementary material for: Organofunctionalized borotungstate polyoxometalates as tunable photocatalysts for oxidative dimerization of amines
Source: Chem Sci. 2024 Aug 12;15(36):14685–91. doi: 10.1039/d4sc03534h (PMC11332656; doi:10.1039/d4sc03534h)
Supplement: SC-015-D4SC03534H-s001 [file SC-015-D4SC03534H-s001.pdf]

Supplementary Information (SI) for Chemical Science.
This journal is © The Royal Society of Chemistry 2024

Supporting Information

Organofunctionalized Borotungstate Polyoxometalates as Tunable Photocatalysts for Oxidative Dimerization of Amines

Nicole Tsang, Alexander J. Kibler, Stephen P. Argent, Hon Wai Lam,* Kieran D. Jones* and Graham N. Newton*

Contents

Contents 2
1 General experimental 3
2 Hybrid POM synthesis & characterisation 5
2.1 Preparation & characterisation 5
$(^{n}Bu_4N)_3H[HBW_{11}O_{39}(P(O)Ph)_2]$ (PBW11) 5
$K_3H[HBW_{11}O_{39}(P(O)Ph)_2]$ (KPBW11) 6
$K_3H[HBW_{11}O_{39}(As(O)Ph)_2]$ (KAsBW11) 8
$(^{n}Bu_4N)_3H[HBW_{11}O_{39}(As(O)Ph)_2]$ (AsBW11) 10
$(^{n}Bu_4N)_4[HBW_{11}O_{39}(PhSiOSiPh)]$ (SiBW11) 11
$(^{n}Bu_4N)_3H[SiW_{11}O_{39}(P(O)Ph)_2]$ (PSiW11) 12
2.2 Cyclic voltammetry studies 14
2.3 UV-Vis Spectroscopic studies 17
3 Hydrolytic Stability Studies 18
4 Experimental Details for Imine Photo-oxidative Cross-Coupling 23
4.1 Photoreactor & Light Source 23
4.2 General Procedures 24
4.3 Reaction Optimisation 25
4.4 Substrate Scope 29
5 References 44
6 NMR Spectra 45
7 Crystallography 57

1 General experimental

Reactions. All stirred reactions were fitted with a polytetrafluoroethylene (PTFE) coated magnetic stirring bar and magnetically stirred using a hot plate magnetic stirrer unless otherwise stated.

Reagents and Solvents. All commercially available reagents were used as received unless otherwise stated. The following materials were prepared according to literature procedures: $K_5[\alpha\text{-}BW_{12}O_{40}]$,[1] $(^nBu_4N)_5[\alpha\text{-}BW_{12}O_{40}]$ (**BW_{12}**),[1] $K_8[HBW_{11}O_{39}]$,[2] $(^nBu_4N)_8[HBW_{11}O_{39}]$ (**BW_{11}**),[2] $(^nBu_4N)_4(\alpha\text{-}SiW_{11}O_{39}(POPh)_2]$ (**$SiBW_{11}$**).[3]

IR Spectra. Attenuated Total Reflection Fourier-transform Infra-red spectroscopy (ATR-FTIR) was recorded on a Bruker Tensor 27 spectrometer equipped with a Pike GladiATR module with a diamond crystal.

NMR Spectra. ^1H, ^{13}C, ^{11}B, ^{19}F, and ^{31}P Nuclear Magnetic Resonance (NMR) spectra were acquired on a Bruker AV(III)400 HD or a Bruker AV(III)500 HD fitted with a 5 mm BBFO and 5 mm prodigy BBO probes, respectively. Processing of all NMR data was conducted with Bruker TopSpin or Mestrelab Research Mnova software suites. All signals δ are reported in parts per million (ppm). Where appropriate, chemical shifts in ^1H and ^{13}C spectra were referenced to the residual (partially) non-deuterated solvent according to Fulmer *et al*.[7] ^{19}F, ^{11}B, and ^{31}P NMR spectra were referenced through the solvent lock (^2H) signal according to the IUPAC-recommended secondary referencing method following Bruker protocols.[8]
Data are reported as follows where appropriate: chemical shift, multiplicity, coupling constant, integration, and assignment. Broad signals are labelled with the prefix “br” (*e.g.,* br s = broad singlet). Complex signals of high order or due to signal overlap, that also display no apparent multiplicity, are reported with m (multiplet). Coupling constants *J* are reported in hertz (Hz).

Mass Spectra. Electrospray ionisation mass spectrometry (ESI-MS) was performed on a Bruker MicroTOF spectrometer. Samples were prepared for analysis by dissolving ca. 1 mg of the solid compound in 1 mL of HPLC grade acetonitrile. 50 µL of this stock solution was then introduced to the spectrometer through an auto-sampler by mixing into a stream of 30:70 H_2O: MeOH. All data was subsequently analysed using the Bruker DataAnalysis

software suite. Modified instrument parameters were used for the detection of high molecular weight polyoxometalate anions while operating in negative mode.

Cyclic Voltammetry. Cyclic voltammetry (CV) was performed on a CHI600E workstation for solution state measurements. A standard three electrode set-up was employed with a glassy carbon (GC) working electrode (d = 3 mm), Pt wire counter electrode, and a $Ag^+|Ag$ reference electrode. CV was carried out on 1 mM of analyte in deoxygenated anhydrous DMF (5 mL) with 0.1 M $^nBu_4NPF_6$ supporting electrolyte, scan rate 100 mVs^{-1}. Redox potentials are referenced to the $Ag^+|Ag$ couple. The GC working electrode was polished using aqueous alumina slurries of decreasing particle size (1, 0.3, and 0.05 µm) and rinsed thoroughly with deionised water (18.2 MΩ cm, MilliQ). The Pt wire auxiliary electrode was cleaned in a butane flame prior to use.

X-ray Crystallography. X-ray diffraction measurements were performed in Experiments Hutch 1 (EH1) of Beamline I19, at Diamond Light Source.[4] The data were collected at a wavelength of 0.6889 Å on a Fluid Film Devices 3-circle fixed-chi diffractometer using a Dectris Pilatus 2M detector. The crystal was mounted on a MiTeGen MicroMountTM using a perfluoropolyether oil and plunged into liquid nitrogen before being cooled for data collection by a Cryostream nitrogen-gas stream.[5] For **$KPBW_{11}$**: Cell parameters were refined from the observed positions of all strong reflections and absorption corrections were applied using a Gaussian numerical method with beam profile correction (CrysAlisPro).[6] For **$KAsBW_{11}$**: The collected frames were integrated using XIA26 software[7] and the data were corrected for absorption effects using AIMLESS,[8] an empirical method. Structures were solved within Olex2[9] by dual space iterative methods (SHELXT)[10] and all non-hydrogen atoms refined by full-matrix least-squares on all unique F2 values with anisotropic displacement parameters (SHELXL).[10] Hydrogen atoms were treated by a mixture of independent and constrained refinement riding geometries with thermal parameters linked to Uiso of their parent atoms. Structures were checked with checkCIF.[11] CCDC 2358933-4 contains the supplementary data for these compounds. These data can be obtained free of charge from The Cambridge Crystallographic Data Centre via www.ccdc.cam.ac.uk/data_request/cif.

UV/Vis. A 12.5 µM solution of analyte in MeCN was prepared and measurements performed on a Cary UV-Vis NIR Spectrometer using a quartz cuvette.

2 Hybrid POM synthesis & characterisation

2.1 Preparation & characterisation

($^{n}Bu_4N)_3H[HBW_{11}O_{39}(P(O)Ph)_2]$ (PBW$_{11}$)

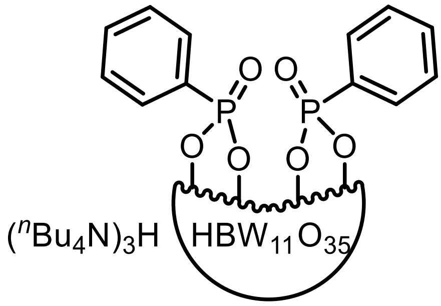

An oven-dried flask equipped with a stirrer bar was charged with $K_8[HBW_{11}O_{39}]•13H_2O$ (250 mg, 0.078 mmol, 1.0 eq.), $^{n}Bu_4NBr$ (150 mg, 0.47 mmol, 6.0 eq.) and placed under an argon environment. Anhydrous MeCN (1 mL) was added followed by dropwise addition of phenylphosphonic dichloride (44 µL, 0.31 mmol, 4.0 eq.) with stirring. The resulting mixture was stirred at reflux for 24 h. The reaction mixture was then cooled to room temperature and subjected to centrifugation (8000 rpm, 5 min) to remove the remaining solid. The separated solution was then diluted with EtOH (150 mL) to give a white suspension, centrifugation (8000 rpm, 5 min) and decantation gave a fine white precipitate which was dried under vacuum before dissolution in minimum MeCN (2 mL). Centrifugation (6000 rpm, 2 min), decantation and a final trituration (Et_2O, 40 mL), centrifugation, decantation, and vacuum drying sequence gave the hybrid POM **PBW$_{11}$** as an off-white solid (228 mg, 0.063 mmol, 81%).

^{1}H NMR (400 MHz, CD_3CN) δ 8.13 – 8.07 (m, 4H, ArC<u>H</u>), 7.64 – 7.61 (m, 2H, ArC<u>H</u>), 7.58 – 7.52 (m, 4H, ArC<u>H</u>), 4.54 (s, 1H, BO<u>H</u>), 3.15 – 3.10 (m, 24H, C<u>H</u>$_2$), 1.62 (tt, J = 8.3, 6.1 Hz, 24H, C<u>H</u>$_2$), 1.38 (sext., J = 7.4 Hz, 24 Hz, C<u>H</u>$_2$), 0.98 (t, J = 7.3 Hz, 36H, C<u>H</u>$_3$).

^{13}C NMR (101 MHz, CD_3CN) δ 132.2 (d, J^{C-P} = 3.0 Hz, 2 × Ar<u>C</u>H), 131.9 (d, J^{C-P} = 11.0 Hz, 4 × Ar<u>C</u>H), 128.5 (d, J^{C-P} = 194.1 Hz, 2 × Ar<u>C</u>), 128.2 (d, J =16.0, 4 × Ar<u>C</u>H), 58.4 (t, J = 2.6 Hz, 12 × <u>C</u>H$_2$), 23.4 (12 × <u>C</u>H$_2$), 19.4 (12 × <u>C</u>H$_2$), 12.9 (12 × <u>C</u>H$_3$).

^{11}B NMR (128 MHz, CD_3CN) δ 2.04 (br s)

^{31}P NMR (162 MHz, CD_3CN) δ 20.34 (tt, J^{H-P} = 14.2, 4.5 Hz, 2 x Ph-<u>P</u>(O)).

ATR-IR (neat, cm^{-1}): 2960, 2934, 2873, 1483, 1380, 1141, 1061, 1006, 958, 892, 825, 777, 647, 527.

Elemental Analysis calc (%) for $[C_{16}H_{36}N]_3H[HBW_{11}O_{39}][POPh]_2$ (3634.60): C, 19.83, H, 3.33, N, 1.16. Found: C, 20.10, H, 3.40, N, 1.07.

$K_3H[HBW_{11}O_{39}(P(O)Ph)_2]$ ($KPBW_{11}$)

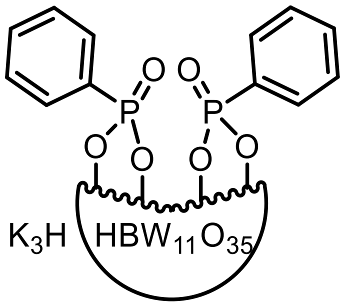

To a stirring solution of phenylphosphonic acid (24 mg, 0.15 mmol, 2.0 eq.) in MeCN (3.9 mL), $K_8[HBW_{11}O_{39}]\cdot 13H_2O$ (250 mg, 0.078 mmol, 1.0 eq.) was added followed by dropwise addition of 4 M HCl (150 µL, 6.0 mmol, 40.0 eq.). The resulting mixture was heated at reflux for 16 h before cooling to room temperature and subjected to centrifugation (8000 rpm, 5 min) to remove any remaining precipitate. The solution was then diluted with Et_2O (45 mL) to give an off-white suspension, centrifugation (8000 rpm, 5 min) and decantation gave a yellow precipitate which was dried under vacuum before dissolution in minimum MeCN (2 mL). Centrifugation (6000 rpm, 2 min), decantation and a final trituration (Et_2O, 40 mL), centrifugation, decantation, and vacuum drying sequence yielded a crude pale-yellow solid. Further purification was achieved via vapour diffusion crystallisation with *tert*-butyl methyl ether (antisolvent) and nitromethane to yield **$KPBW_{11}$** as a pale-yellow crystalline solid (130 mg, 0.043 mmol, 55%).

^{1}H NMR (500 MHz, CD_3CN) δ 8.14 – 8.08 (m, 4H, ArC<u>H</u>), 7.66 – 7.62 (m, 2H, ArC<u>H</u>), 7.59 – 7.55 (m, 4H, ArC<u>H</u>), 4.53 (s, 1H, BO<u>H</u>).

^{13}C NMR (126 MHz, CD_3CN) δ 132.2 (d, $J^{C\text{-}P}$ = 3.2 Hz, 2 × Ar<u>C</u>), 132.9 (d, $J^{C\text{-}P}$ = 11.2 Hz, 4 × Ar<u>C</u>H), 129.2 (d, $J^{C\text{-}P}$ = 16.0 Hz, 4 × Ar<u>C</u>H), 129.1 (d, $J^{C\text{-}P}$ = 194.2 Hz, 2 × Ar<u>C</u>P).

^{11}B NMR (128 MHz, CD_3CN) δ 1.93 (br s).

^{31}P NMR (202 MHz, CD_3CN) δ 20.38 (tt, $J^{H\text{-}P}$ = 14.2, 4.5 Hz, 2 × Ph-<u>P</u>(O)).

ATR-IR (neat, cm^{-1}): 3603, 1619, 1439, 1138, 1054, 971, 896, 826, 567.

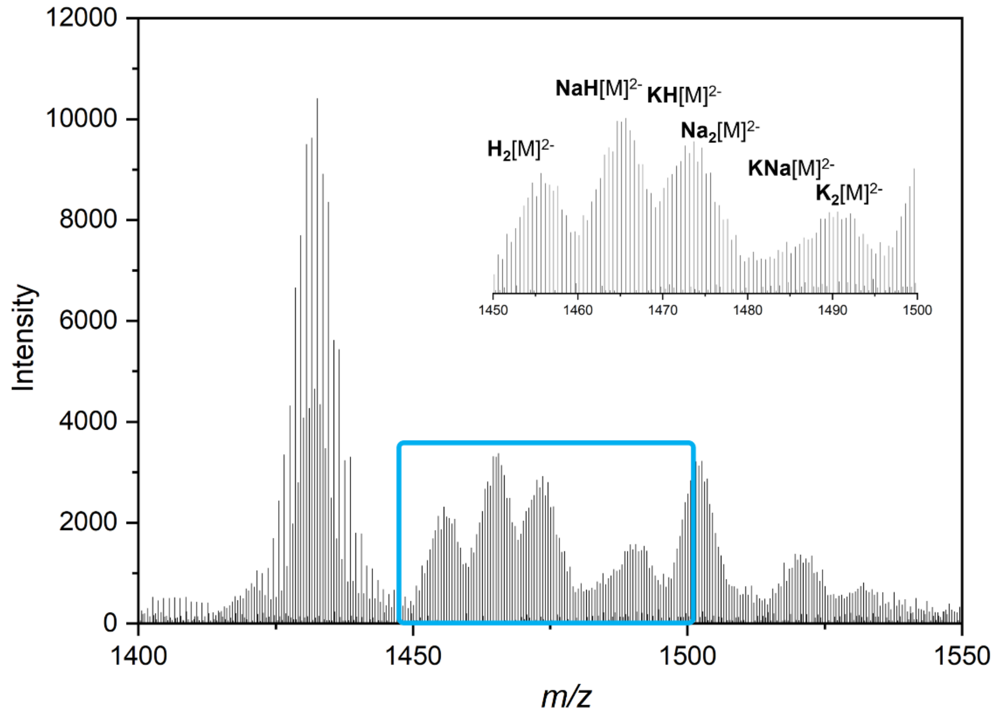

Figure S1. Negative mode ESI-MS spectrum of compound **KPBW₁₁** in the *m/z* range 1400-1550. [M] = $[HBW_{11}O_{39}(P(O)C_6H_5)_2]^{4-}$.

Table S1. Mass spectra (ESI) assignments of **KPBW₁₁**.

Assignment	z	*m/z* (calc.)	*m/z* (obs.)
**H_2**$[HBW_{11}O_{39}(POC_6H_5)_2]$	2−	1453.66	1453.66
**NaH**$[HBW_{11}O_{39}(POC_6H_5)_2]$	2−	1464.65	1464.65
**KH**$[HBW_{11}O_{39}(POC_6H_5)_2]$	2−	1472.63	1472.64
**Na_2**$[HBW_{11}O_{39}(POC_6H_5)_2]$	2−	1475.64	1475.65
**KNa**$[HBW_{11}O_{39}(POC_6H_5)_2]$	2−	1483.62	1483.62
**K_2**$[HBW_{11}O_{39}(POC_6H_5)_2]$	2−	1492.11	1492.13

$K_3H[HBW_{11}O_{39}(As(O)Ph)_2]$ ($KAsBW_{11}$)

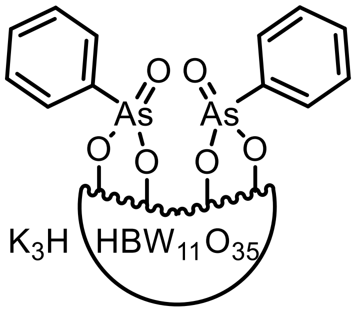

To a stirring mixture of $K_8[HBW_{11}O_{39}]•13H_2O$ (500 mg, 0.16 mmol, 1.0 eq.) and phenyl arsonic acid (63 mg, 0.30 mmol, 2.0 eq.) in MeCN (7.8 mL), 4 M HCl (150 µL, 6.0 mmol, 40.0 eq.) was added dropwise before heating at reflux for 16 h. The white mixture was then cooled to room temperature and subjected to centrifugation (8000 rpm, 5 min) to remove any remaining precipitate before trituration with Et_2O (45 mL), centrifugation (8000 rpm, 5 min) and decantation to give a crude white powder. Dissolution in minimum MeCN (2 mL) followed by centrifugation (6000 rpm, 2 min), decantation and a final trituration (Et_2O, 40 mL), centrifugation, decantation, and vacuum drying sequence yielded hybrid POM **$KAsBW_{11}$** as a white solid (290 mg, 0.093 mmol, 58%).

^{1}H NMR (400 MHz, CD_3CN) δ 8.21 – 8.18 (m, 4H, ArC<u>H</u>), 7.81 – 7.7 (m, 2H, ArC<u>H</u>), 7.74 – 7.70 (m, 4H, ArC<u>H</u>), 4.90 (s, 1H, BO<u>H</u>).
^{13}C NMR (101 MHz, CD_3CN) δ 135.1 (2 × Ar<u>C</u>H), 132.4 (4 × Ar<u>C</u>H), 130.8 (4 × Ar<u>C</u>H), 130.1 (2 × Ar<u>C</u>).
^{11}B NMR (128 MHz, CD_3CN) δ 2.13 (br s).
ATR-IR (neat, cm^{-1}): 3505, 2255, 1612, 1441, 1404, 1368, 1218, 1085, 1015, 968, 898, 783, 524, 466.

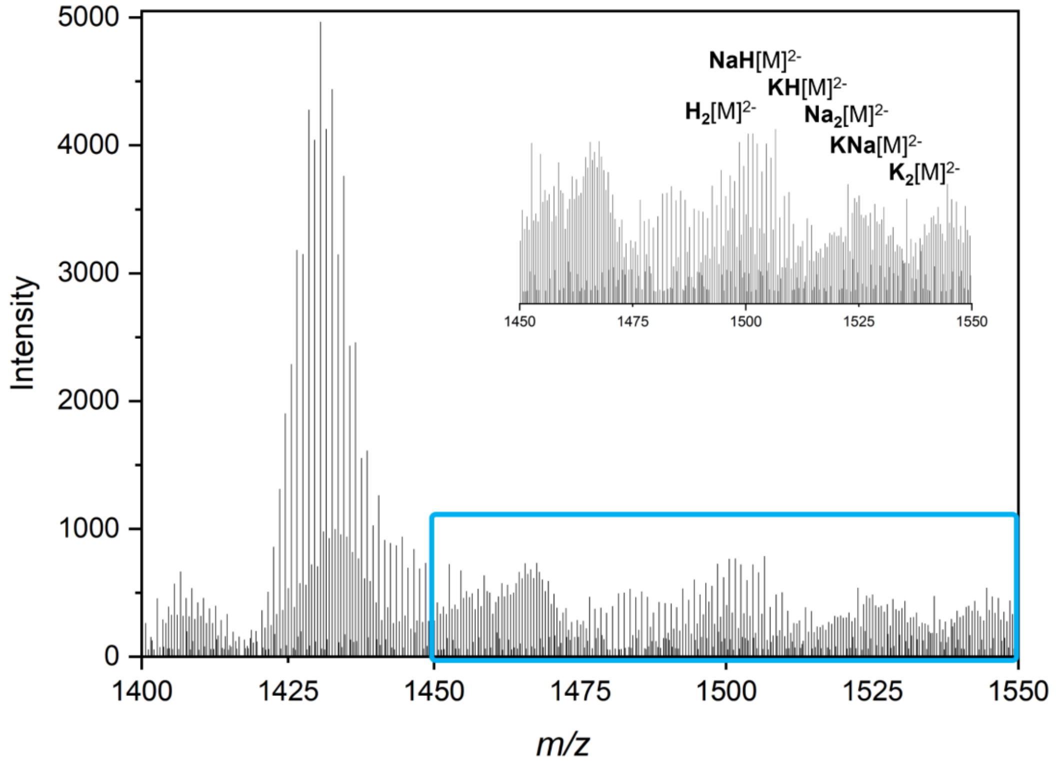

Figure S2. Negative mode ESI-MS spectrum of compound **KAsBW$_{11}$** in the *m/z* range 1400-1550. [M] = [$HBW_{11}O_{39}(As(O)C_6H_5)_2$]$^{4-}$.

Table S2. Mass spectra (ESI) assignments of **KAsBW$_{11}$**.

Assignment	*z*	*m/z* (calc.)	*m/z* (obs.)
**H$_2$**[$HBW_{11}O_{39}(AsOC_6H_5)_2$]	2−	1497.60	1497.58
**NaH**[$HBW_{11}O_{39}(AsOC_6H_5)_2$]	2−	1508.59	1508.58
**KH**[$HBW_{11}O_{39}(AsOC_6H_5)_2$]	2−	1516.58	1516.56
**Na$_2$**[$HBW_{11}O_{39}(AsOC_6H_5)_2$]	2−	1519.59	1519.61
**KNa**[$HBW_{11}O_{39}(AsOC_6H_5)_2$]	2−	1527.57	1527.58
**K$_2$**[$HBW_{11}O_{39}(AsOC_6H_5)_2$]	2−	1536.06	1536.08

(^{n}Bu$_4$N)$_3$H[HBW$_{11}$O$_{39}$(As(O)Ph)$_2$] (AsBW$_{11}$)

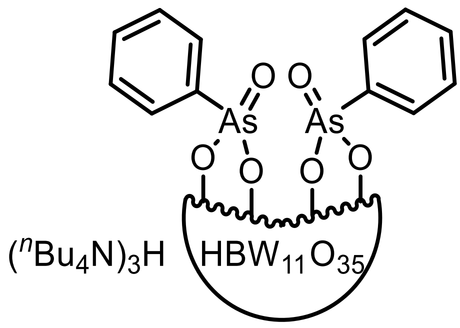

A solution of ^{n}Bu$_4$NBr (58 mg, 0.18 mmol, 6.0 eq.) in MeCN (2 mL) was added in one portion to a stirring solution of K$_3$H[HBW$_{11}$O$_{39}$(As(O)Ph)$_2$] (**KAsBW$_{11}$**, 100 mg, 0.032 mmol, 1.0 eq.) in MeCN (5 mL) and the resulting mixture was stirred for 1 h at room temperature. Centrifugation (8000 rpm, 5 min) of the resulting suspension, decantation, and concentration *in vacuo* gave the crude alkyl ammonium salt as a white solid. The solid was redissolved in a minimum MeCN (~5 mL), aided by sonication, followed by centrifugation (8000 rpm, 5 min) and decantation to remove any remaining fine precipitate. Trituration with Et$_2$O (40 mL) resulted in a white suspension which was subjected to centrifugation (8000 rpm, 5 min), decantation, and vacuum drying of the resulting white powder. Dissolution in minimum MeCN (2 mL) followed by centrifugation (6000 rpm, 2 min), decantation and a final trituration (Et$_2$O, 40 mL), centrifugation, decantation, and vacuum drying sequence gave hybrid POM **AsBW$_{11}$** as a white solid (90.4 mg, 0.024 mmol, 75%).

^{1}H NMR (400 MHz, CD$_3$CN) δ 8.20 – 8.18 (m, 4H, ArC$\underline{H}$), 7.81 – 7.77 (m, 2H, ArC$\underline{H}$), 7.74 – 7.70 (m, 4H, ArC$\underline{H}$), 4.91 (s, 1H, BO$\underline{H}$), 3.12 – 3.08 (m, 24H, C$\underline{H}_2$), 1.61 (ddd, *J* = 16.2, 9.0, 6.1 Hz, 24H C$\underline{H}_2$), 1.37 (sext., *J* = 7.4 Hz, 24H, C$\underline{H}_2$), 0.97 (t, *J* = 7.3 Hz, 36H, C$\underline{H}_3$).
^{13}C NMR (101 MHz, CD$_3$CN) δ 135.1 (2 × Ar$\underline{C}$H), 132.4 (4 × Ar$\underline{C}$H), 130.7 (4 × Ar$\underline{C}$H), 130.1 (2 × Ar$\underline{C}$), 59.3 (d, *J* = 2.9 Hz, 12 × $\underline{C}$H$_2$), 24.3 (12 × $\underline{C}$H$_2$), 20.3 (12 × $\underline{C}$H$_2$), 13.8 (12 × $\underline{C}$H$_3$).
^{11}B NMR (128 MHz, CD$_3$CN) δ 2.13 (br s).
ATR-IR (neat, cm^{-1}): 3549, 2960, 2873, 1615, 1483, 1380, 1219, 1089, 1010, 965, 900, 791, 738, 714, 687, 527, 429.
Elemental Analysis calc (%) for [C$_{16}$H$_{36}$N]$_3$H[HBW$_{11}$O$_{39}$][AsOPh]$_2$[C$_4$H$_{10}$O] (3796.62): C, 20.25, H, 3.45, N, 1.11. Found: C, 20.18, H, 3.44, N, 0.81.

(^{n}Bu$_4$N)$_4$[HBW$_{11}$O$_{39}$(PhSiOSiPh)] (SiBW$_{11}$)

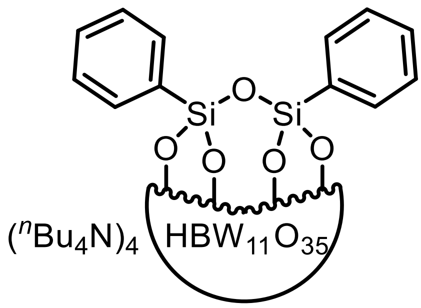

To a stirred solution of K$_8$[HBW$_{11}$O$_{39}$]•13H$_2$O (250 mg, 0.078 mmol, 1.0 eq.) in MeCN:H$_2$O (2:1, 7.5 mL), trimethoxyphenylsilane (44 µL, 0.23 mmol, 3.0 eq.) was added followed by the dropwise addition of 4M HCl (78 µL, 0.32 mmol, 4.0 eq.). The mixture was stirred at room temperature for 6 h before centrifugation (8000 rpm, 5 min) to remove the remaining solid. ^{n}Bu$_4$NBr (154.7 mg, 0.48 mmol, 6.0 eq.) in water (3 mL) was added to the resultant solution in one portion to give a white suspension. The suspension was centrifuged (8000 rpm, 5 min) and the solvent decanted off. The remaining white solid was redissolved in a minimum MeCN (2 mL), aided by sonication, before centrifugation (6000 rpm, 2 min) and decantation to remove any remaining fine precipitate. Trituration with Et$_2$O (40 mL) resulted in a white suspension which was subjected to centrifugation, decantation, and vacuum drying to give the hybrid POM **SiBW$_{11}$** as a white solid (173.4 mg, 0.045 mmol, 58%).

^{1}H NMR (400 MHz, CD$_3$CN) δ 7.90 – 7.87 (m, 4H, ArC$\underline{H}$), 7.49 – 7.43 (m, 6H, ArC$\underline{H}$), 4.85 (s, 1H, BO$\underline{H}$), 3.16 – 3.11 (m, 32H, C$\underline{H}_2$), 1.67 – 1.59 (m, 32H, C$\underline{H}_2$), 1.39 (sext., *J* = 7.4 Hz, 32H, C$\underline{H}_2$), 0.98 (t, *J* = 7.3 Hz, 48H, C$\underline{H}_3$).
^{13}C NMR (101 MHz, CD$_3$CN) *δ* 134.6 (2 x Ar$\underline{C}$H), 132.6 (2 x Ar$\underline{C}$), 130.5 (4 x Ar$\underline{C}$H), 128.0 (4 x Ar$\underline{C}$H), 58.3 (t, *J* = 2.6 Hz, 16 × $\underline{C}$H$_2$), 23.4 (16 × $\underline{C}$H$_2$), 20.3 (16 × $\underline{C}$H$_2$), 13.9 (16 × $\underline{C}$H$_3$).
^{11}B NMR (128 MHz, CD$_3$CN) *δ* 2.08 (br s).
ATR-IR (neat, cm^{-1}): 2961, 2874,1483, 1381, 1239, 1132, 1038, 1005, 946, 878, 822, 767, 713, 594, 559, 530.
Elemental Analysis calc (%) for [C$_{16}$H$_{36}$N]$_4$[HBW$_{11}$O$_{39}$][Si$_2$O(Ph)$_2$] (3854.28): C, 23.68, H, 4.05, N, 1.45. Found: C, 23.23, H, 4.00, N, 1.23.

$(^nBu_4N)_3H[SiW_{11}O_{39}(P(O)Ph)_2]$ (PSiW$_{11}$)

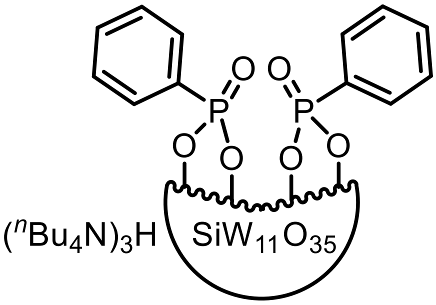

Hybrid POM **PSiW$_{11}$** was prepared according to procedure described by Hill and co-workers.[3] The spectral data were in accordance with those reported in the literature.

^{1}H NMR (400 MHz, CD_3CN) δ 8.13 – 8.07 (m, 4H, ArC<u>H</u>), 7.63 – 7.59 (m, 2H, ArC<u>H</u>), 7.57 – 7.53 (m, 4H, ArC<u>H</u>), 3.13 – 3.08 (m, 24H, C<u>H</u>$_2$), 1.66 – 1.58 (m, 24H, C<u>H</u>$_2$), 1.38 (sext., *J* = 7.4 Hz, 24H, C<u>H</u>$_2$), 0.98 (t, *J* = 7.3 Hz, 36H, C<u>H</u>$_3$).

^{13}C NMR (101 MHz, CD_3CN) δ 132.9 (d, $J^{C\text{-}P}$ = 3.1 Hz, 2 × Ar<u>CH</u>), 132.7 (d, $J^{C\text{-}P}$ = 10.9 Hz, 4 × Ar<u>C</u>H), 130.2 (2 x <u>C</u>), 130.1 (d, $J^{C\text{-}P}$ = 197.6 Hz, 2 × Ar<u>C</u>), 129.2 (d, $J^{C\text{-}P}$ = 15.9 Hz, 4 × Ar<u>C</u>H), 59.3 (t, *J* = 2.9 Hz, 12 × <u>C</u>H$_2$), 24.3 (12 x <u>C</u>H$_2$), 20.3 (12 × <u>C</u>H$_2$)), 13.8 (12 x <u>C</u>H$_3$).

^{31}P{^{1}H} NMR (162 MHz, CD_3CN) δ 17.53 – 17.36 (m, 2 × Ph-<u>P</u>(O)).

Elemental Analysis calc (%) for $[C_{16}H_{36}N]_3H[SiW_{11}O_{39}][POPh]_2$ (3650.85): C, 19.74, H, 3.29, N, 1.15. Found C, 20.10, H, 3.32, N, 0.61.

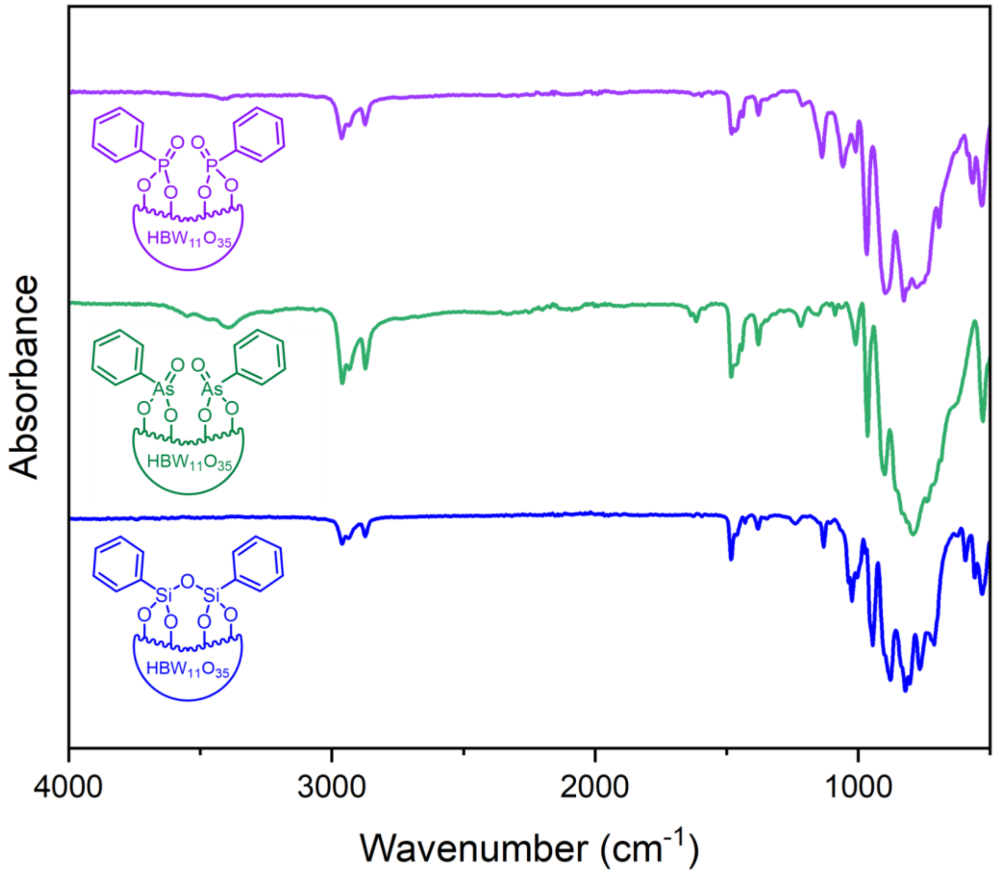

Figure S3. Stacked ATR-IR spectra of hybrid borotungstates **PBW_{11}**, **$AsBW_{11}$**, and **$SiBW_{11}$**.

2.2 Cyclic voltammetry studies

Cyclic Voltammetry. All cyclic voltammograms (CVs) were recorded using 1 mM of analyte in anhydrous DMF with 0.1 M $^{n}Bu_4NPF_6$ supporting electrolyte at 100 mVs^{-1} using a glassy carbon working electrode (d = 3 mm and A = 0.071 cm^2), Pt wire counter electrode and a $AgNO_3$|Ag non-aqueous reference electrode. Please see **General Experimental 1.1** for further details.

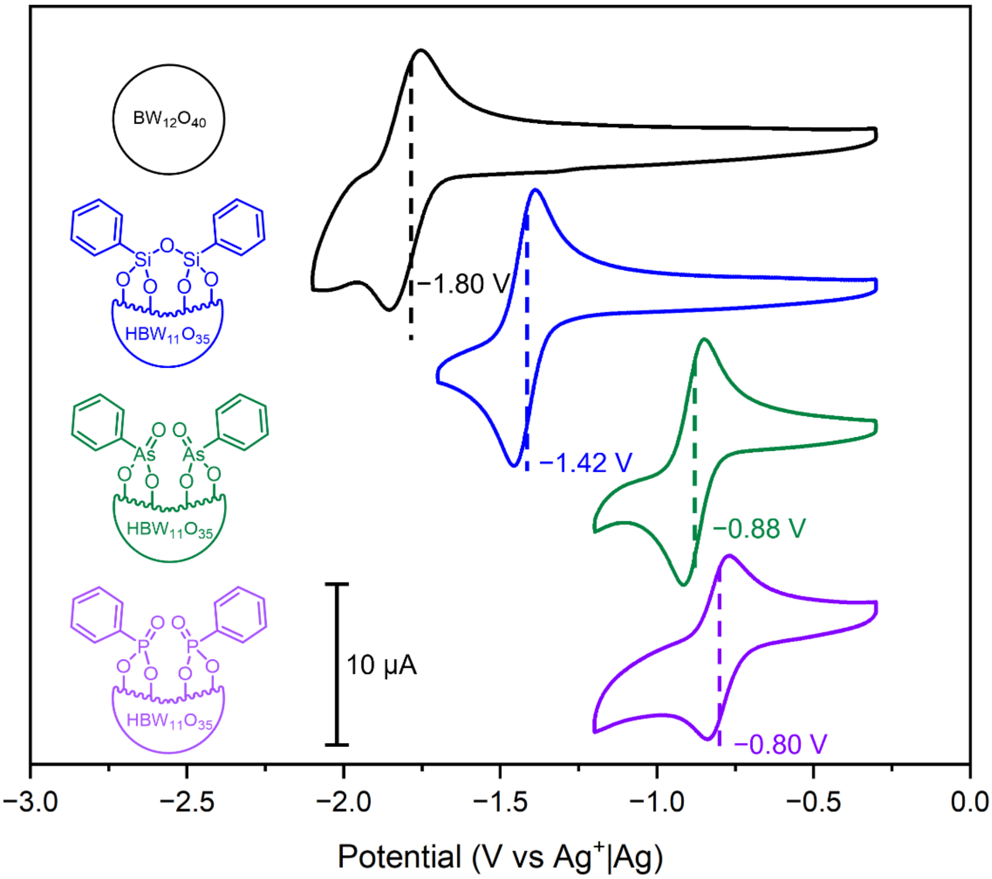

Figure S4. Stacked CVs of borotungstates **BW_{12}**, **$SiBW_{11}$**, **$AsBW_{11}$**, and **PBW_{11}** with the standard potentials indicated for the first redox process for each POM.

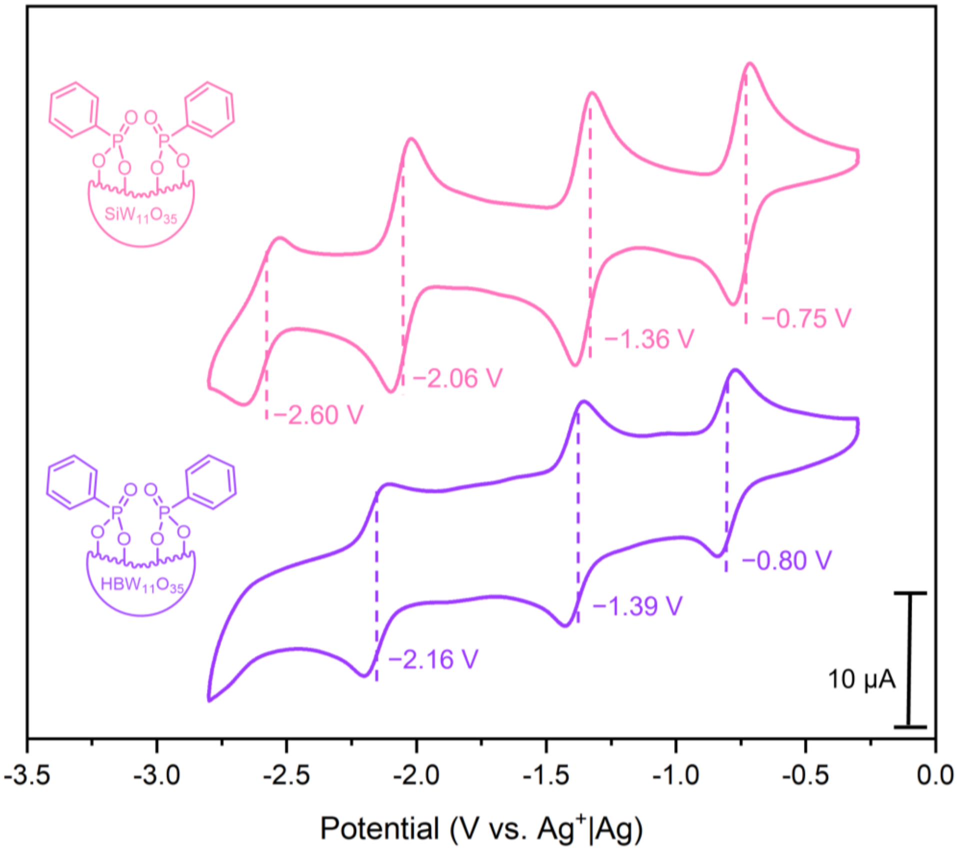

Figure S5. Stacked CVs of hybrid POMs **PSiW11** and **PBW11** showing multiple quasi-reversible redox events for both silico- and boro-tungstate phenylphosphonate hybrid POMs.

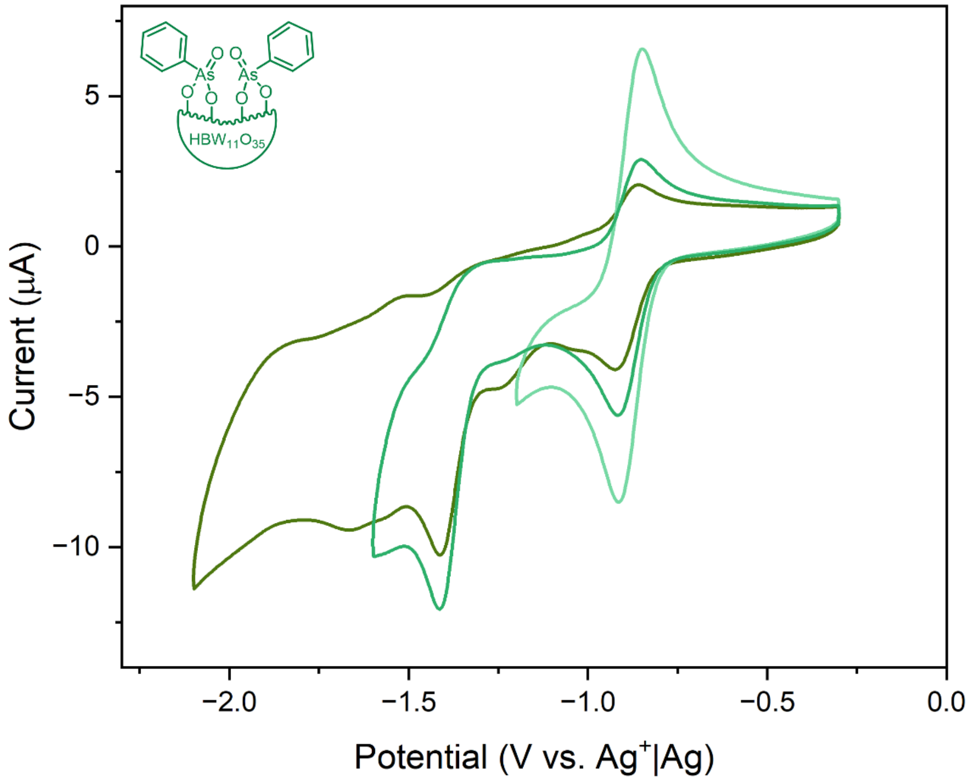

Figure S6. Overlayed CVs of POM **AsBW11** across increasingly negative potentials highlighting the instability of **AsBW11** towards multiple redox process.

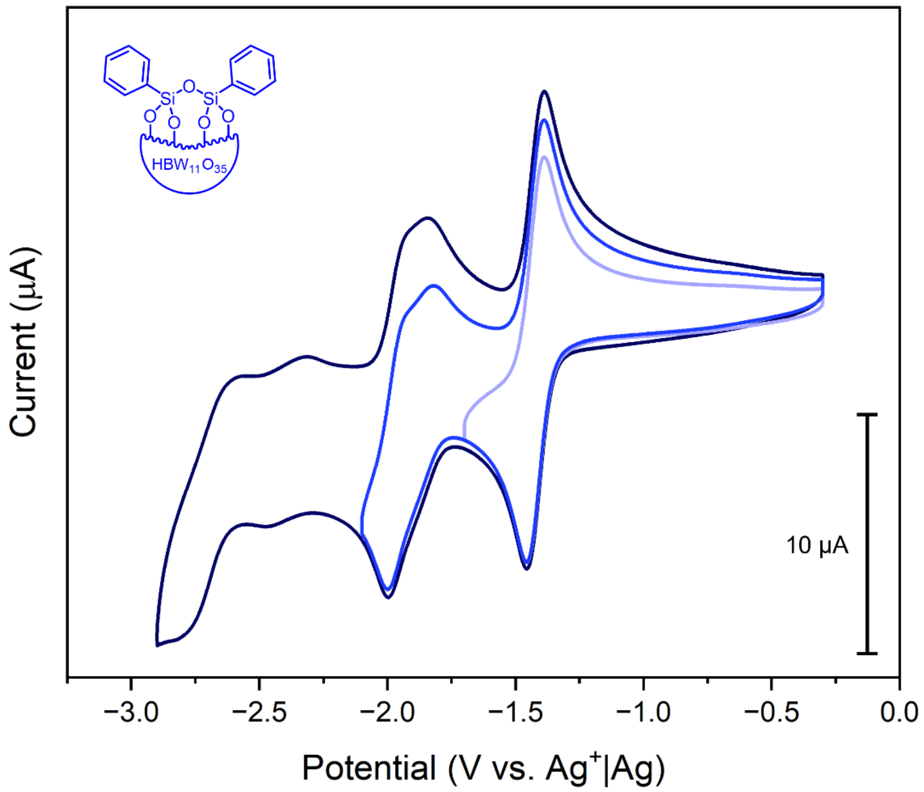

Figure S7. Overlayed CVs of POM **SiBW11** across increasingly negative potentials highlighting the instability of **SiBW11** towards multiple redox process.

2.3 UV-Vis Spectroscopic studies

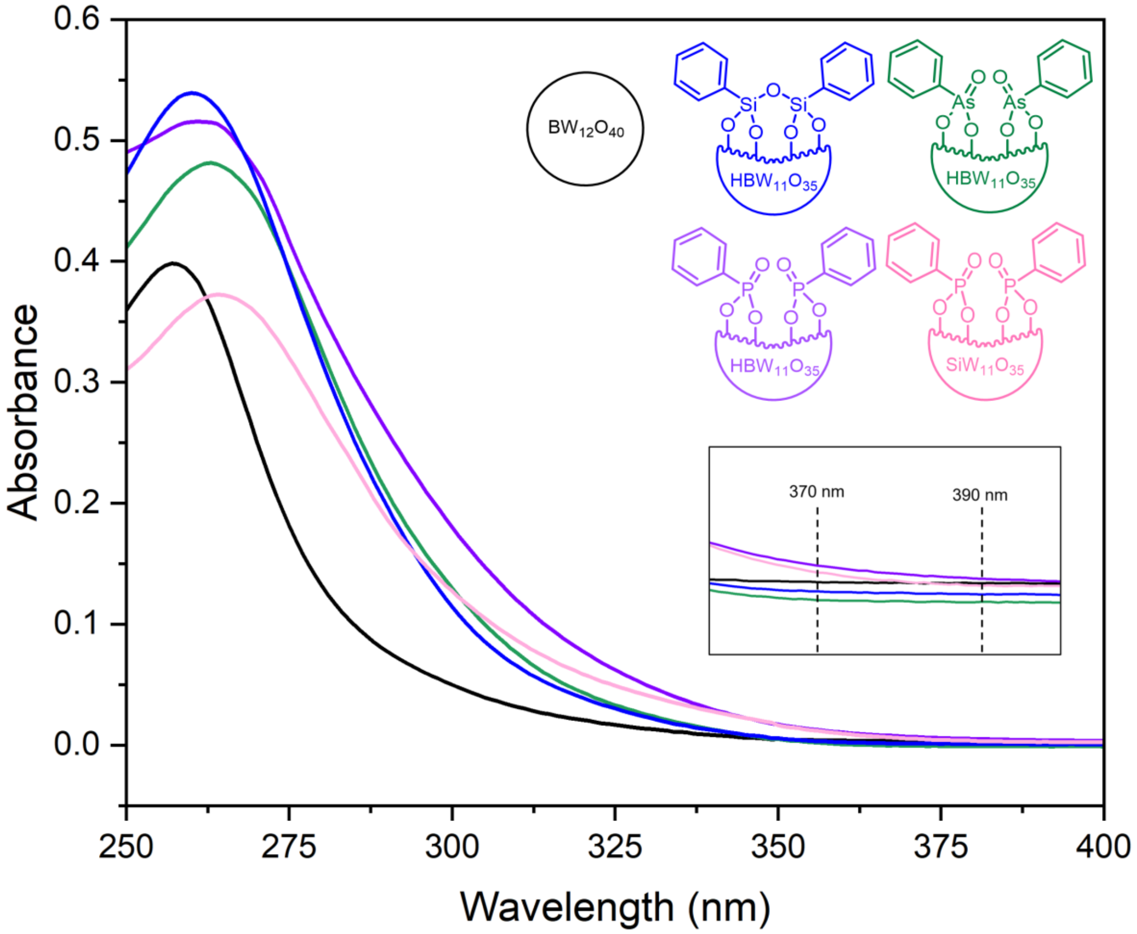

Figure S8. UV-vis absorption spectrum of compounds **BW_{12}, PBW_{11}**, **$AsBW_{11}$**, **$SiBW_{11}$**, and **$PSiW_{11}$** in MeCN at 12.5 µM.

3 Hydrolytic Stability Studies

Method: A sample of polyoxometalate (0.006 mmol) was dissolved in 9:1 (v/v) DMSO-d_6:D_2O (0.8 mL) and NMR spectra taken periodically over the course of weeks. These solutions were kept in their NMR tubes and stored in the dark under ambient conditions between measurements. No special precautions were taken to avoid exposure to the atmosphere.

To date, no hydrolytic studies have been conducted on organic-inorganic hybrid borotungstates. Considering the oxidative cross-coupling reaction of amines contains hydrolysis mediators, such as basic amines, proton donors, and water, the hydrolytic stability of hybrid POMs **PBW_{11}**, **$AsBW_{11}$**, and **$SiBW_{11}$** is an important factor when considering their use as catalysts. Previous hydrolysis studies of phenylphosphonate-hybridised Keggin POMs of general formula $[(PhP(O))_2X^{n+}W_{11}O_{39}]^{(8-n)-}$ (X = P^{5+}, Si^{4+}) have shown these clusters to be relatively unstable.[3] For the phosphotungstate Keggin POM (**Scheme S1**, **a**), partial cleavage of the phosphoryl group occurred when dissolved in wet DMSO. The silicotungstate Keggin POM (**Scheme S1**, **b**) was shown to slowly (days) undergo complete hydrolytic cleavage of the phosphoryl group from the POM cluster under similar conditions to give $PhPO_3^{2-}$ and the lacunary precursor complex $SiW_{11}O_{39}^{8-}$, no intermediate partially hydrolysed species were observed.

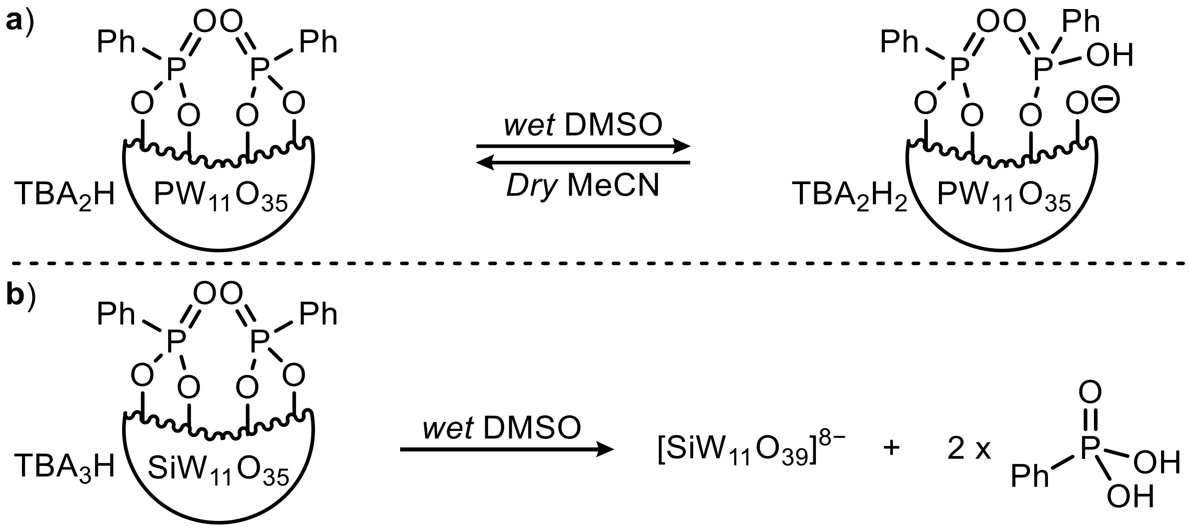

Scheme S1. Hydrolysis pathways of phenylphosphonate-hybridised Keggin POMs.[3]

The stability of organophosphonate hybrid **PBW_{11}** is similar to that of the isoionic silicotungstate **$PSiW_{11}$**, with a slow rate of hydrolysis (over days) and no observable partially hydrolysed intermediate indicated by ^{31}P NMR. The only observed peaks are attributed to starting material (20.42 ppm) and free phosphonate (12.97 ppm) (**Figure S9**). While new

peaks in the aromatic region of the ^{1}H NMR spectrum (between 7.4-8 ppm) were observed (**Figure S10**), the B-O-H resonance was retained over the course of 7 days.

Organoarsonate hybrid **AsBW$_{11}$**, the poorest performing photocatalyst in the series, was found to be immediately hydrolysed when solubilised in the D_2O doped DMSO-d$_6$. This is supported by the disappearance of the B-O-H peak at 4.81 ppm in the ^{1}H NMR spectrum (**Figure S12**) and a shift in the peaks corresponding to the aromatic protons at time point 0 (effectively minutes from addition). Furthermore, a sharp singlet peak in the ^{11}B NMR spectrum at 1.76 ppm was also observed (**Figure S13**), suggesting that following cleavage of the organoarsonate, the metastable lacunary **BW$_{11}$** reacts further to give the favoured plenary Keggin species **BW$_{12}$**.[12] The propensity of **AsBW$_{11}$** towards hydrolysis may account for its poor catalytic performance.

Conversely, the organosilyl POM **SiBW$_{11}$** was found to have excellent hydrolytic stability, even over a month, with no apparent change in the aromatic region of the ^{1}H NMR spectrum and consistent ^{11}B NMR spectroscopic data (**Figure S14 & S15**). Notably, over the period of a week the relative integration of the B-O-H peak at 4.72 ppm in the ^{1}H NMR spectra decreased. Proton-deuterium exchange (B–OH/D) is proposed to have occurred while retaining the hybrid POM structure, as indicated by the ^{1}H and ^{11}B NMR spectroscopic data.

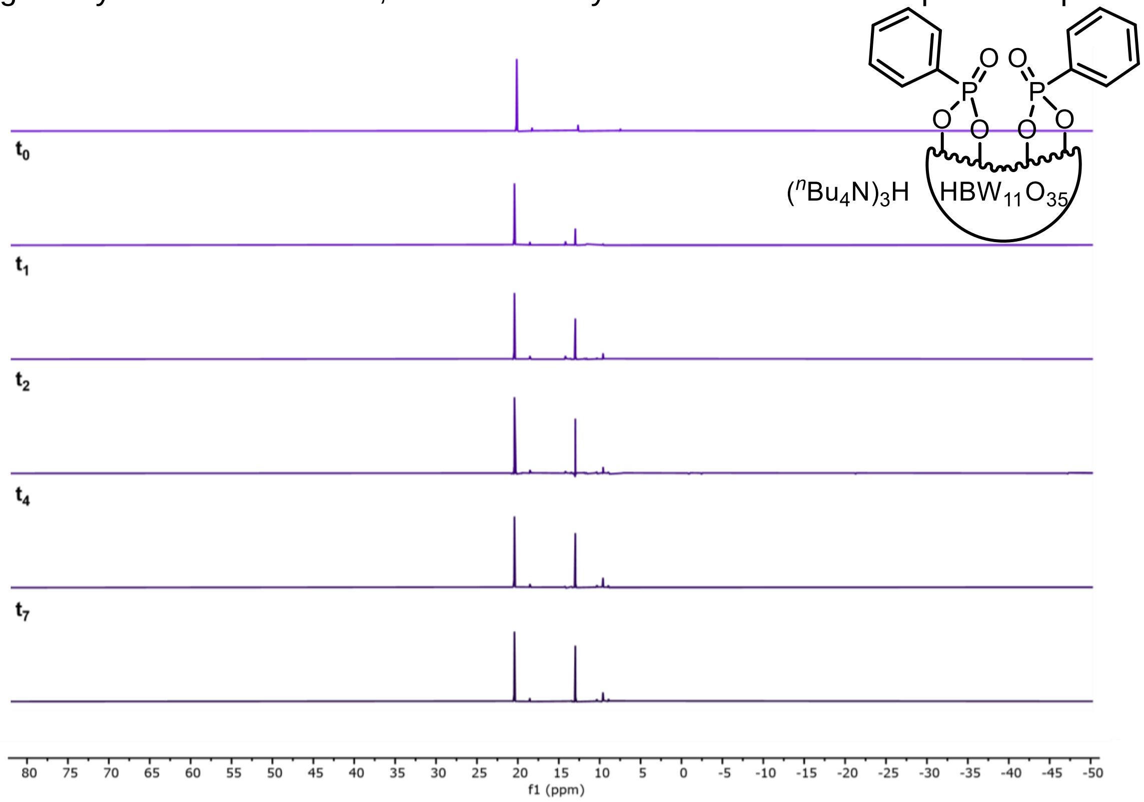

Figure S9. Solution of **PBW$_{11}$** in 9:1 (v/v) DMSO-d$_6$:D_2O monitored by ^{31}P NMR (162 MHz) over t$_{days}$.

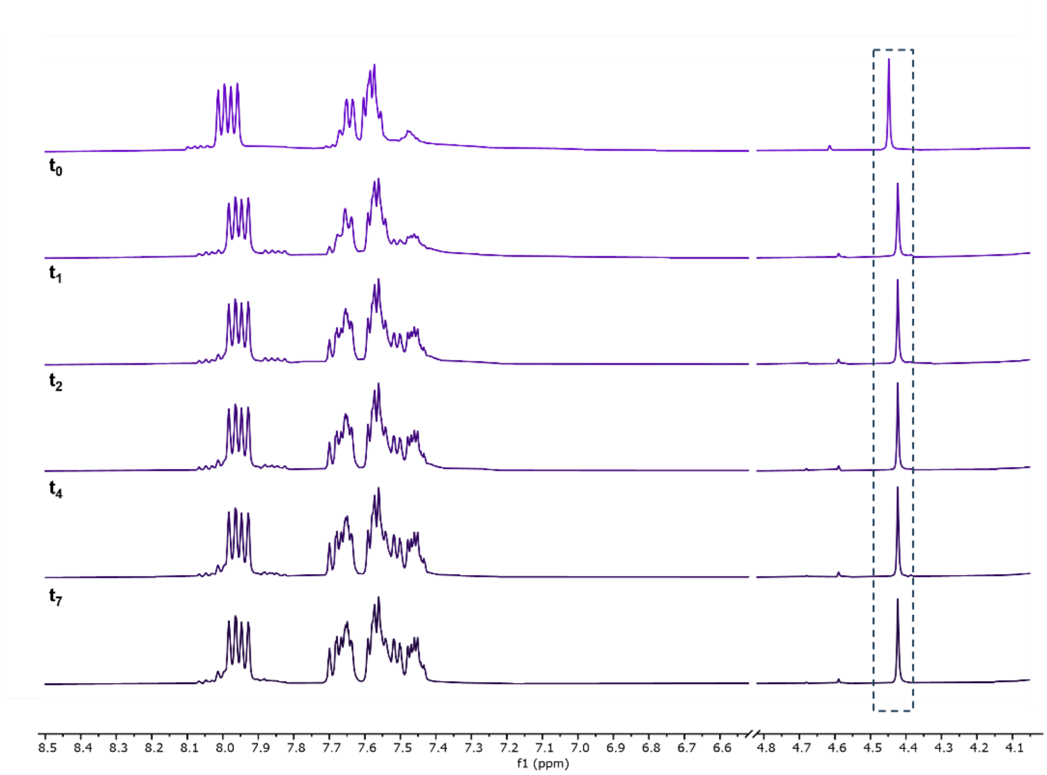

Figure S10. Solution of **PBW_{11}** in 9:1 (v/v) DMSO-d_6:D_2O monitored by ^{1}H NMR (400 MHz) over t_{days}. Spectra window restrained to highlight areas of interest.

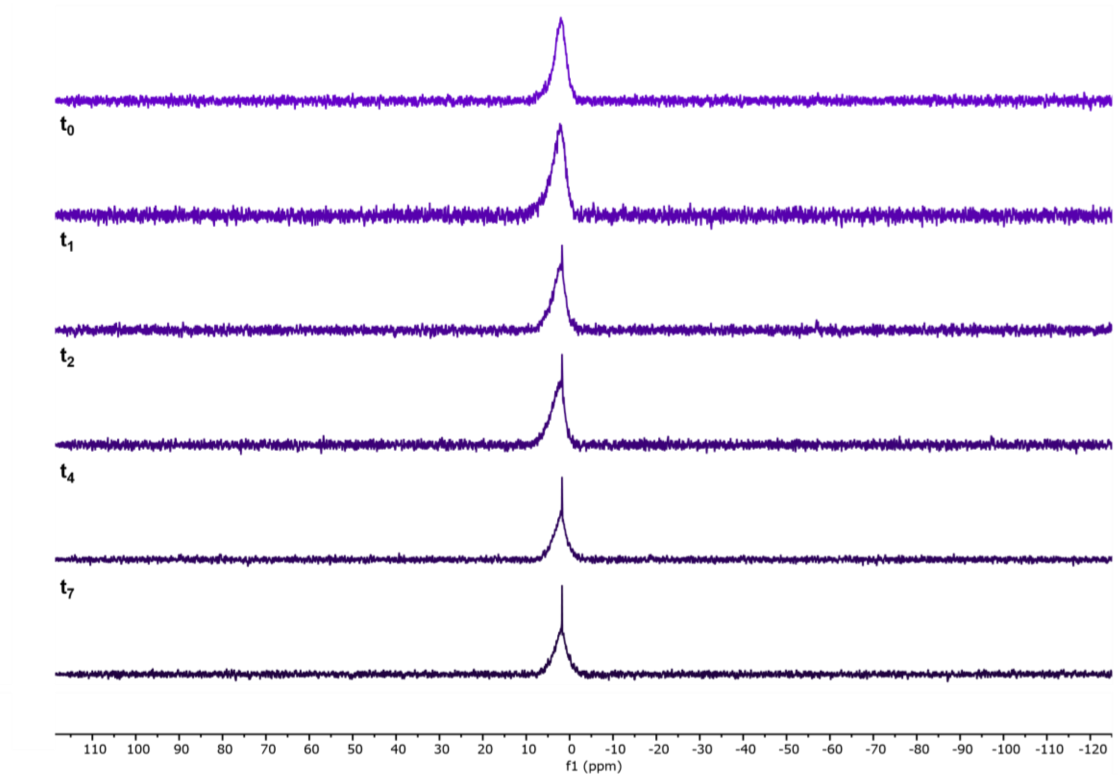

Figure S11. Solution of **PBW_{11}** in 9:1 (v/v) DMSO-d_6:D_2O monitored by ^{11}B NMR (129 MHz) over t_{days}.

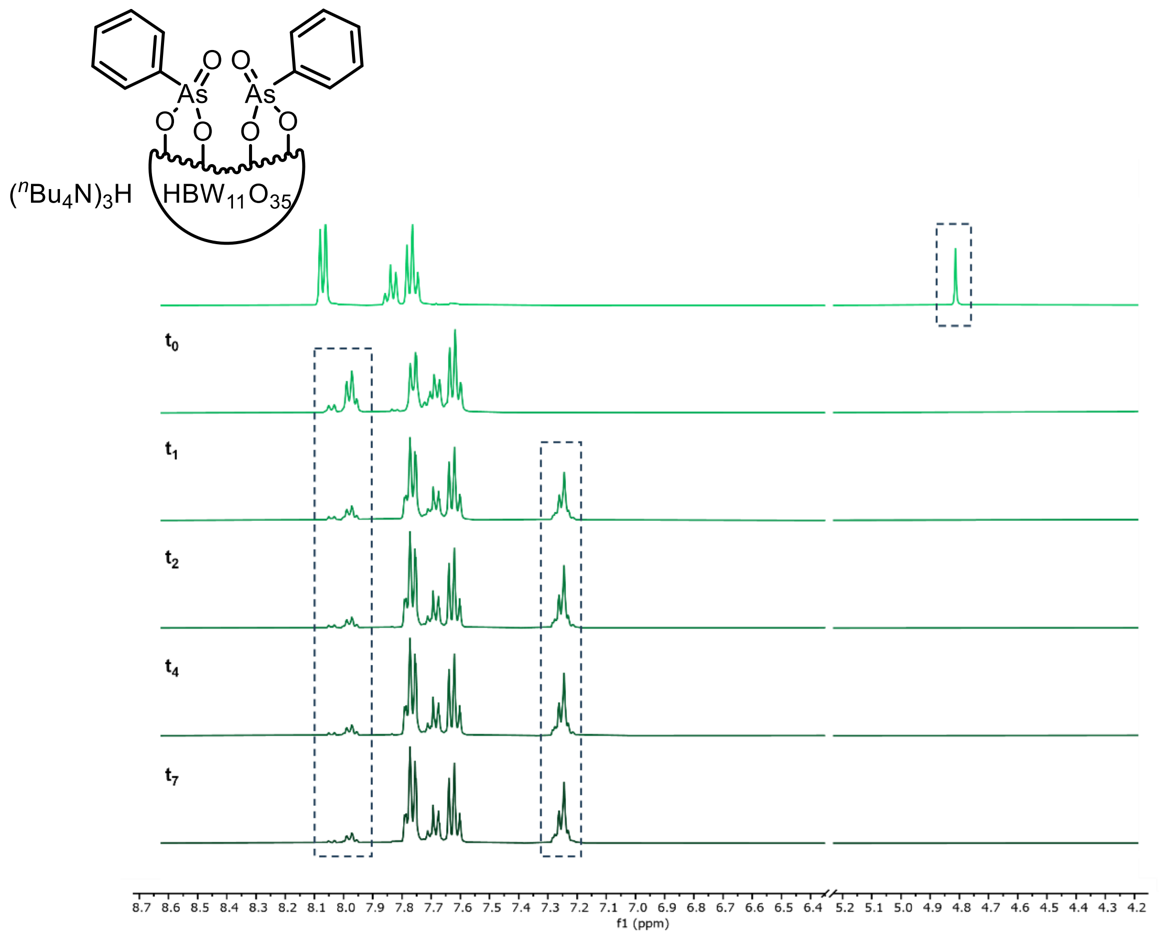

Figure S12. Solution of **AsBW$_{11}$** in 9:1 (v/v) DMSO-d$_6$:D$_2$O monitored by ^{1}H NMR (400 MHz) over t$_{days}$. Spectra window restrained to highlight areas of interest. The characteristic B-O-H peak and new/shift in the peaks corresponding to aromatic protons are highlighted.

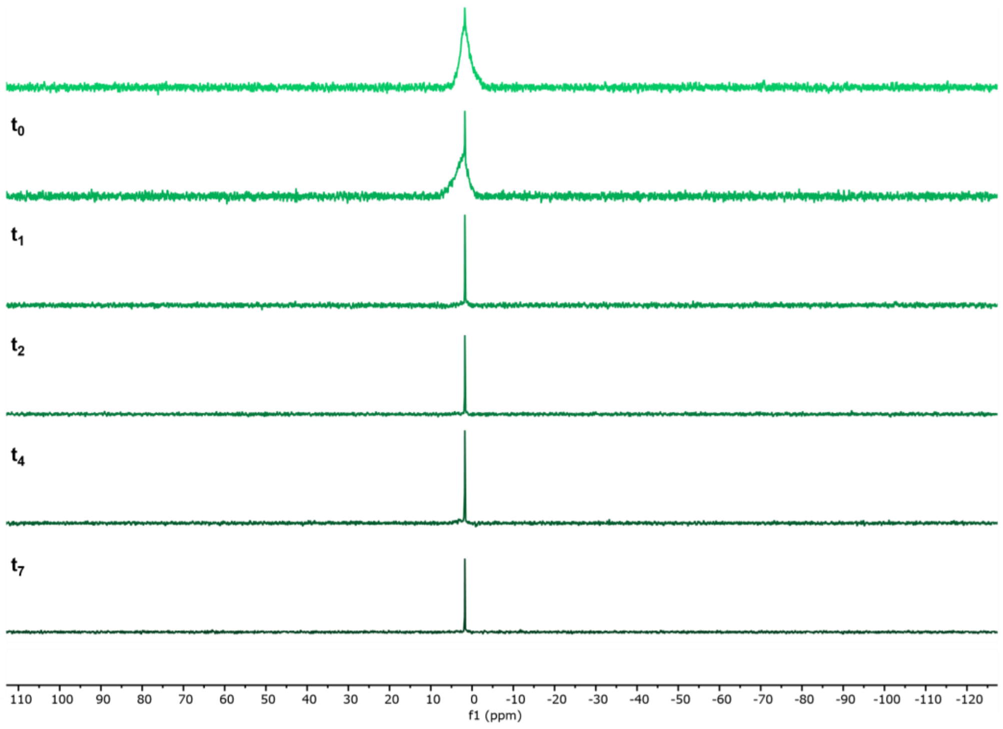

Figure S13. Solution of **AsBW$_{11}$** in 9:1 (v/v) DMSO-d$_6$:D$_2$O monitored by ^{11}B NMR (129 MHz) over t$_{days}$. Spectra window restrained to highlight areas of interest.

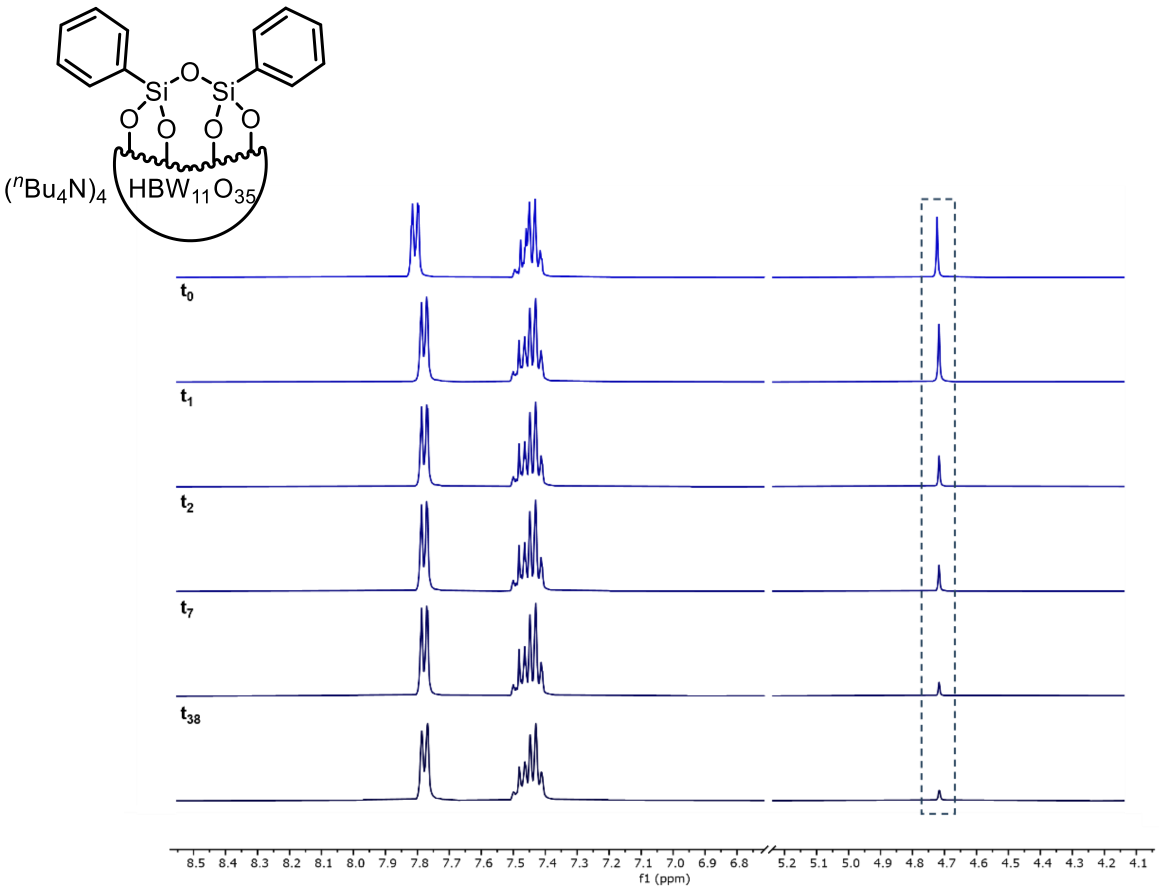

Figure S14. Solution of **$SiBW_{11}$** in 9:1 (v/v) DMSO-d_6:D_2O monitored by ^{1}H NMR (400 MHz) over t_{days}. Spectra window restrained to highlight areas of interest.

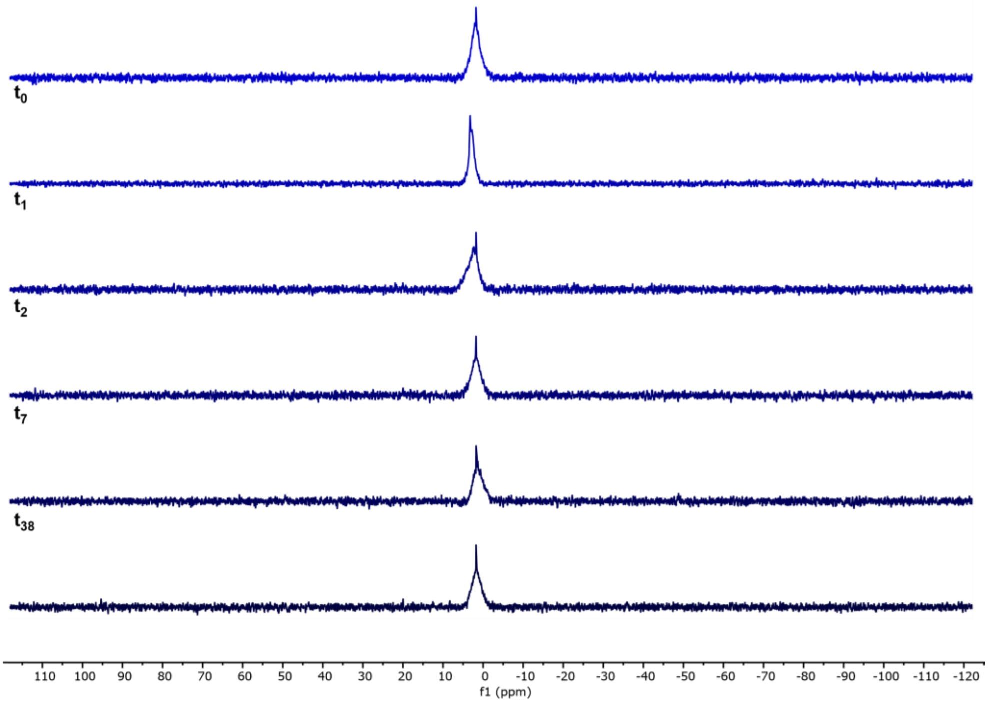

Figure S15. Solution of **$SiBW_{11}$** in 9:1 (v/v) DMSO-d_6:D_2O monitored by ^{11}B NMR (129 MHz) over t_{days}. Spectra window restrained to highlight areas of interest.

4 Experimental Details for Imine Photo-oxidative Cross-Coupling

4.1 Photoreactor & Light Source

The EvoluChem PhotoRedOx Box™ (US Patent #10,906,022) and a Kessil 43W PR-160L-370nm or 40 W H160 Tuna Flora (blue setting) LED light were used in all photochemical reactions. Only the front four vial slots closest to the lamp were employed for reaction reproducibility. Reactions were stirred at 800 rpm. An in-built fan was used to maintain the air temperature inside the box at no more than 8°C above room temperature.

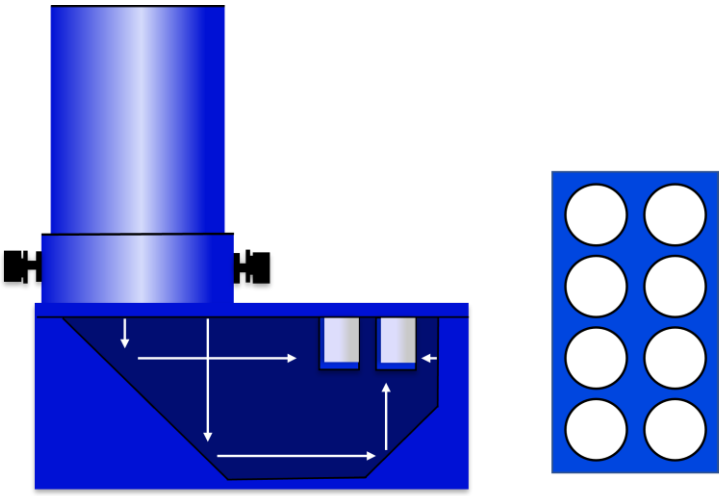

Figure S16. *Left*) Diagram representation of the EvoluChem PhotoRedOx Box^{TM}. *Right*) Aerial viewpoint of the vial slot positions.

4.2 General Procedures

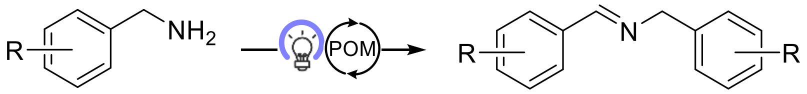

Anaerobic rection conditions (General procedure A): To an oven-dried microwave vial fitted with a magnetic stirrer, POM (4 mol%) was added and the vial sealed with a septum-lined cap. The vial was placed under vacuum and backfilled with argon (3 times). Degassed MeCN† (1 mL) was added followed by benzylamine (22 µL, 0.2 mmol) and the solution was stirred while irradiated. The resulting mixture was concentrated *in vacuo,* 1,3-benzodioxole:$CDCl_3$ stock solution (0.7 mL, 0.14 M) added, and the resulting suspension was subjected to centrifugation (6000 rpm, 2 min) to remove any fine precipitate. The resultant solution was analysed by NMR spectroscopy.

Oxygen containing reaction Conditions (General procedure B): To an oven-dried microwave vial fitted with a magnetic stirrer, POM (4 mol%) was added followed by MeCN† (1 mL) and benzylamine (22 µL, 0.2 mmol). The vial was sealed with a septum-lined cap, sparged with oxygen if using,‡ and then stirred while irradiated. The resulting mixture was concentrated *in vacuo,* 1,3-benzodioxole:$CDCl_3$ stock solution (0.7 mL, 0.14 M) added, and the resulting suspension was subjected to centrifugation (6000 rpm, 2 min) to remove any fine precipitate. The resultant solution was analysed by NMR spectroscopy.

Optimised reaction conditions (General procedure C): To an oven-dried microwave vial fitted with a magnetic stirrer, hybrid POM **PBW_{11}** (2 mol%) was added followed by MeCN† (0.5 mL) and amine (0.2 mmol). The vial was sealed with a septum-lined cap, sparged with oxygen for 1 min and then stirred while irradiated at 370 nm for 24 h. The resulting mixture was concentrated *in vacuo,* 1,3-benzodioxole:$CDCl_3$ stock solution (0.7 mL, 0.14 M) added, and the resulting suspension was subjected to centrifugation (6000 rpm, 2 min) to remove any fine precipitate. The resultant solution was analysed by NMR spectroscopy.

† Solvent undried and obtained from commercial vendors used without further purification.

‡ Reactions conducted under an oxygen atmosphere, solutions undergo sparging with oxygen for 1 min prior to irradiation at this stage.

4.3 Reaction Optimisation

Table S3. POM photocatalyzed oxidative dimerization of benzylamine (**1a**).

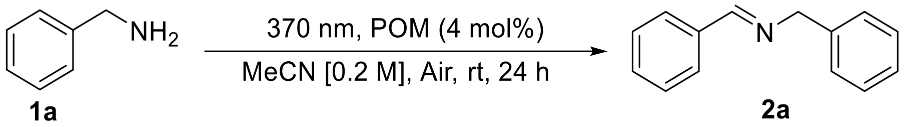

Entry	POM	NMR Yield (%)
1	No POM	0
2	**BW_{12}**	10
3	**BW_{11}**	20
4	**PBW_{11}**	36
5	**$AsBW_{11}$**	32
6	**$SiBW_{11}$**	37
7	**$SiBW_{11}$ (Dark)**	0
8	**$PSiW_{11}$**	27
9	**TBADT**	5

Reaction conditions: Benzylamine (**1a**, 0.2 mmol) and POM (4 mol%) in acetonitrile (1 mL) were irradiated at 370 nm for 24 h according to general procedure B unless otherwise stated. NMR yield reported using 1,3-benzodioxole as an internal standard.

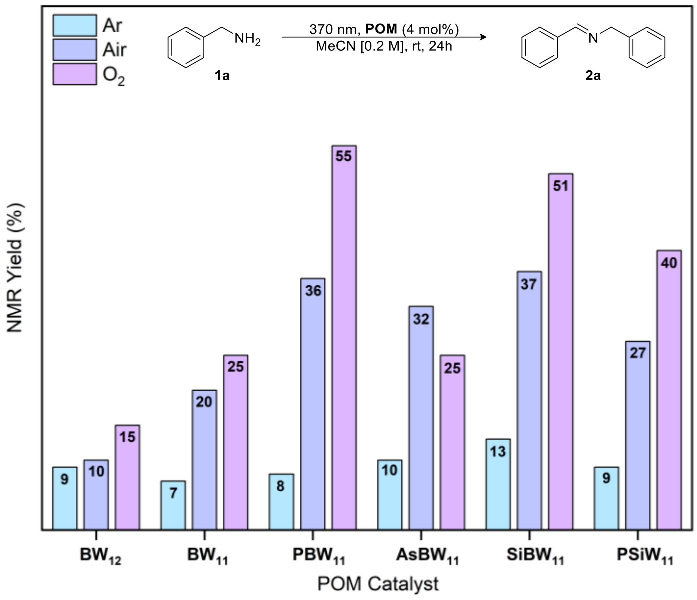

Figure S17. Bar graph displaying NMR yield in different reaction environments. Reactions were conducted using general procedures A (Ar) or B (Air | O_2) depending on required atmosphere. NMR yield reported using 1,3-benzodioxole as an internal standard.

Table S4. Time study for dimerization reaction of benzylamine (**1a**) catalysed by hybrid POM **PBW_{11}**.

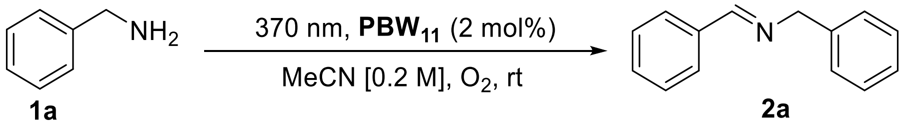

Entry	Time (h)	NMR Yield (%)
**1**	6	18
**2**	18	54
**3**	48	67
**4**	54	53
**5**	72	13
**6**	90	9

Reaction conditions: Benzylamine (**1a**, 0.2 mmol) and hybrid POM **PBW_{11}** (2 mol%) in acetonitrile (1 mL) were irradiated at 370 nm for the described time frame according to a modified general procedure C. NMR yield reported using 1,3-benzodioxole as an internal standard.

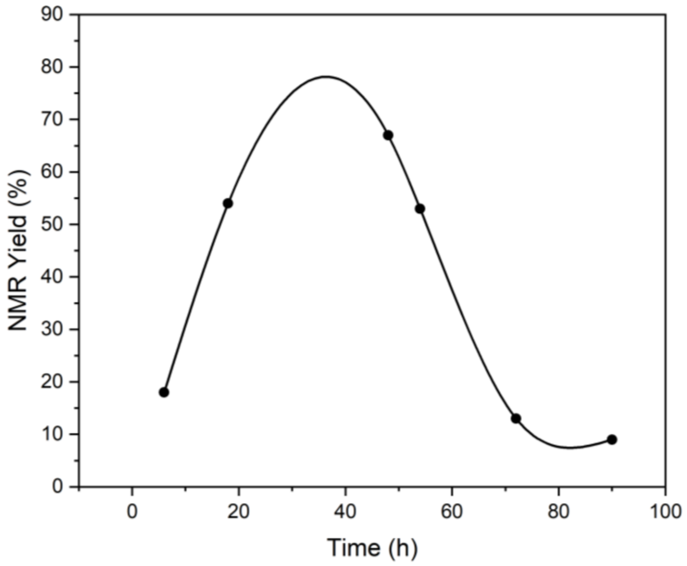

Figure S18. Graph displaying NMR yield over time. The trendline is fitted to a 4th order polynomial.

Table S5. Reaction optimisation for phenylphosphonate hybrid borotungstate catalyst.

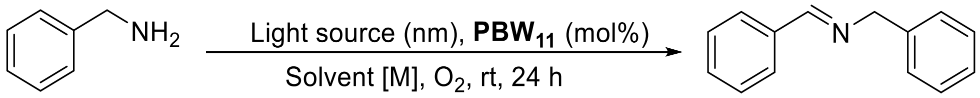

Entry	Light Source (nm)	Catalyst Loading (mol %)	Solvent	Conc. [M]	NMR Yield (%)	Deviation from Model (%)
**1**	**370**	**2**	**MeCN**	**0.2**	**52**	**Model**
2	370	4	MeCN	0.2	55	6
3	370	0.5	MeCN	0.2	38	-27
4	370	6	MeCN	0.2	55	6
5	370	2	DMF	0.2	49	-6
6	370	2	Acetone	0.2	0	-100
7	370	2	Pivalonitrile	0.2	42	-19
8	370	2	MeCN	0.05	56	8
**9**	**370**	**2**	**MeCN**	**0.4**	**57**	**10**
10	370	2	MeCN	0.8	49	-6
11	390	2	MeCN	0.4	4	-92
12	-	2	MeCN	0.4	0	-100
13	370	0	MeCN	0.2	0	-100

Reaction conditions: Benzyl amine (**1a**, 0.2 mmol) and hybrid POM **PBW$_{11}$** in the described solvent were irradiated for 24h at room temperature under an atmosphere of O_2, a modified general procedure C was used. NMR yield reported using 1,3-benzodioxole as an internal standard.

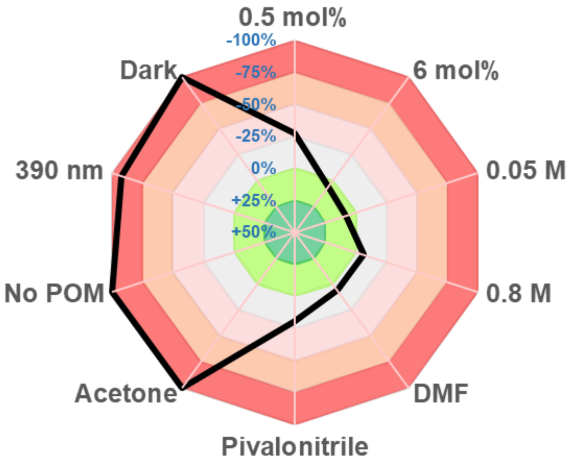

Figure S19. Radar graph summarising deviation in yield from model reaction presented in Table S5. Radar graph adapted from original report from Glorius and co-workers.[13]

Table S6. Summary of POM catalysts for the oxidative dimerization of amines.

Year	POM Cluster	Reaction conditions	Yield
**This work**	$(^nBu_4N)_3H[HBW_{11}O_{39}(P(O)Ph)_2]$	POM (2 mol%), MeCN, rt<br>O_2 (1 atm), 370 nm, 24 h	44-62%[†]
**2014[a]**	$(^nBu_4N)_6[\{Ce(H_2O)\}_2\{Ce(CH_3CN)\}_2(\mu_4\text{-}O)(\gamma\text{-}SiW_{10}O_{36})_2]$	POM (1 mol%), MeCN, 30 °C<br>O_2 (1 atm), >400 nm, 24-54 h	76-96%[‡]
**2018[b]**	$Na_4[NiMo_6O_{18}(OH)_6]$	POM (1 mol%), MeCN, rt<br>O_2 (1 atm), >400 nm, 12 h	63-99%[‡]
**2019[c]**	$(NH_4)_{41}H_7[K_3(H_2O)_3(P_2W_{15}Ta_3O_{62})_6(Mo_2O_4CH_3CO_2)_3(MoO_3)_2]\cdot 85H_2O$	POM (1 mol%), MeCN, 25 °C<br>air (1 atm), solar sim. 20-24 h	53-94%[‡]
**2019[d]**	$\{[Zn(PYI)_3][H_3PW_{11}O_{39}Zn(PYI)]\}\cdot 3H_2O$<br>PYI = pyrrolidine-2-ylimidazole	POM (0.03 mol%), MeCN, rt<br>10 W white LED, air, 36 h	85-95%[†]
**2021[e]**	$\{Cu_4(DPNDI)4(\text{-}CH_3CN)_2[SiW_{12}O_{40}]\}4H_2O$<br>DPNDI = N,N'-bis(4-pyridylmethyl)naphthalene diimide	POM (10 mg/mmol amine), MeCN, 25°C<br>O_2 (1 atm), 10 W white LED,16 h	76-96%[‡]
**2022[f]**	$Na_{13}H_5[Ru_4(H_2O)_2(Cl)_2(WO_2)_4(AsW_9O_{33})_4]\cdot 43H_2O$	POM (0.1 mol %), neat, rt<br>10 W white LED, O_2 (1.0 atm), 24 h	81-95%
**2022[g]**	$K_6H[\{Ru_2Cl(H_2O)(CH_3COO)_2\}\ \{WO(H_2O)\}_2(PW_9O_{34})_2]\cdot 14H_2O$	POM (0.1 mol %), neat, rt<br>10 W white LED, O_2 (1.0 atm), 24 h	87-99%[‡]
**2022[h]**	$Cs_2NaH_5[(Ru(OH))_2O(W_5O_{18})_2]\cdot 18H_2O$<br>$Na_{5.5}H_{6.5}[(W_5O_{18})(Ru_2W_8O_{31})]\cdot 28H_2O$	POM (0.2 mol %), neat, rt<br>10 W white LED, O_2 (2 atm), 24 h	73-99%[‡]
**2022[i]**	POM-based MOF: $[Ru(bpy)_2(H_2dcbpy)]Cl_2$ (H_2dcbpy = 2,2′-bipyridine-5,5′-dicarboxylic acid)<br>& $[PMo_{11}MoO_{40}]^{4-}$	POM (0.1 mol %), MeCN, rt O_2 (1 atm)<br>10 W white LED 30-120 min	73-99%[‡]
**2023[j]**	$H_2\{[Na(H_2O)_4]_2[Rh_2(OAc)_4]_2(SiW_{12}O_{40})\}\cdot 11H_2O$	POM (0.2 mol %), neat, rt<br>10 W white LED O_2 (2 atm), 12 h	72-98%[‡]

[†]NMR yield.; [‡]GCMS yield. [a]Suzuki, K. *et al.*, *Angew. Chem. Int. Ed.*, **2014**, 53, 5356.; [b]H, Yu. et al., *ChemCatChem*., **2018**, 10, 4274.; [c]Li, S. et al., *ACS Appl. Mater. Interfaces*., **2019**, 11, 46, 43287. [d]Shi, Z. et al., *Inorg. Chem.*, **2019**, 58, 12529.;[e]Li, J., et al., *J Mater Sci*, **2021**, 56, 6676.; [f]Li, G. et al., *Inorg. Chem.*, **2022**, 61, 9935. [g]Chen, W. et al., *Inorg. Chem.*, **2022**, 61, 2076. [h]Zhao, Y. et al., *Inorg. Chem.*, **2022**, 61, 12097.; [i] Liu, Y. et al., *ACS Appl. Mater. Interfaces*., **2022** 14, 27882. [j]Liu, Y. et al., Inorg. Chem. **2023**, 62, 12954.

4.4 Substrate Scope

N-Benzylidenebenzylamine (2a)

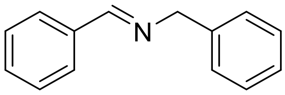

General procedure C was followed using benzylamine (**1a**, 22 µL, 0.2 mmol) to produce *N*-benzylidenebenzylamine (**2a**) in 57% NMR yield, while in the absence of hybrid POM catalyst, no imine **2a** was detected by ^{1}H NMR spectroscopy. The spectral data were in accordance with those reported in the literature.[14]

^{1}H NMR (400 MHz, $CDCl_3$) *δ* 8.41 (br s, 1H, CH), 7.82 – 7.78 (m, 2H, ArCH), 7.43 (dd, *J* = 5.2, 1.9 Hz, 3H, ArCH), 7.36 (d, *J* = 4.4 Hz, 4H, ArCH), 7.30 - 7.28 (m, 1H, ArCH), 4.85 (d, *J* = 1.5 Hz, 2H, CH_2).

HRMS (ESI) Calc. for $C_{14}H_{14}N$ $[M+H]^+$ 196.1121, Found 196.1116.

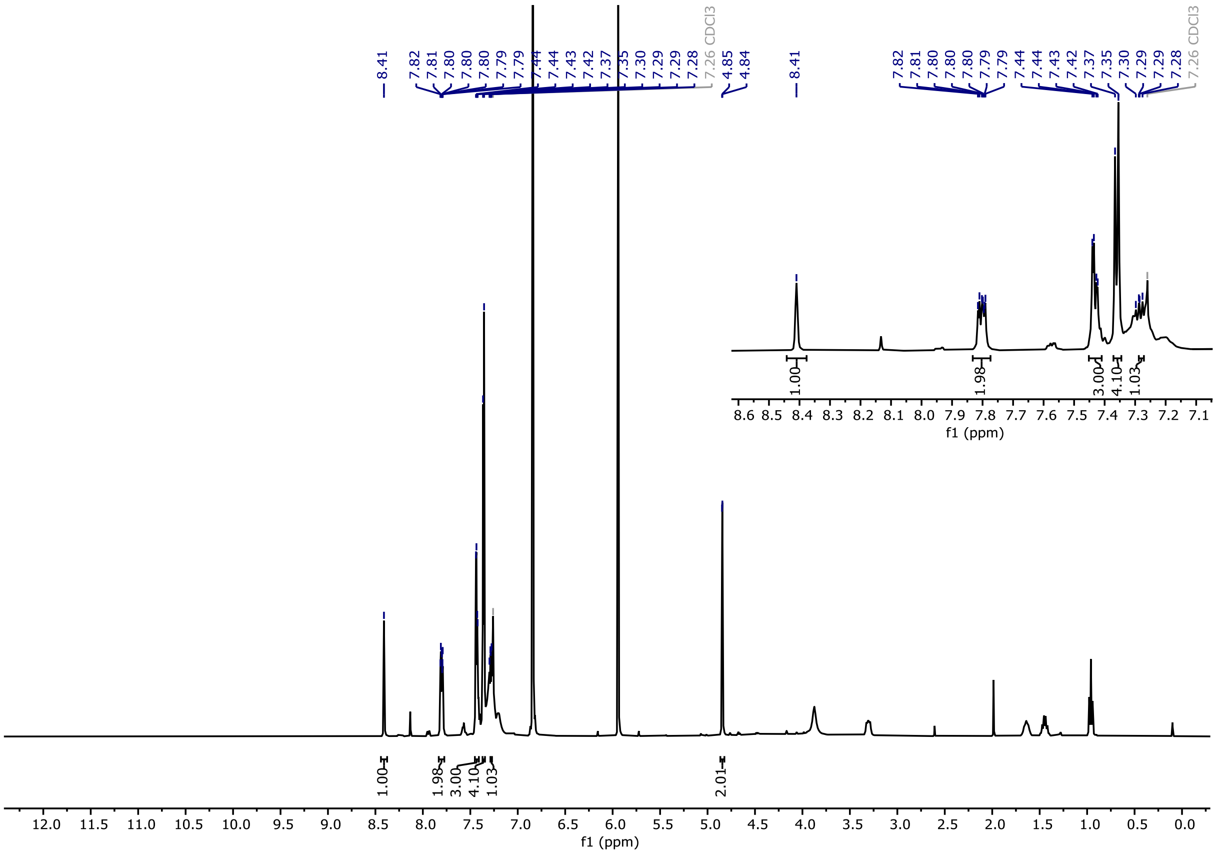

Figure S20. ^{1}H NMR (400 MHz, $CDCl_3$) of **2a**.

1-Cyclohexyl-*N*-(cyclohexylmethyl)methanimine (2b)

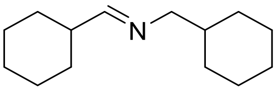

General procedure C was followed using cyclohexylmethanamine (**1b**, 26 µL, 0.2 mmol) to produce 1-cyclohexyl-*N*-(cyclohexylmethyl)methanimine (**2b**) in 8% NMR yield, while in the absence of hybrid POM catalyst imine **2b** was prepared in 2% NMR yield as determined by ^{1}H NMR spectroscopy. The spectral data were in accordance with those reported in the literature.[18]

^{1}H NMR (400 MHz, $CDCl_3$) δ 7.41 (d, J = 5.3 Hz, 1H, C<u>H</u>), 3.18 (d, J = 6.5 Hz, 2H, C<u>H</u>$_2$), 2.21 – 2.11 (m, 1H, C<u>H</u>$_2$), 1.82 – 1.60 (m, 12H, C<u>H</u>$_2$), 1.28 – 1.10 (m, 9H, C<u>H</u>$_2$), 0.94 – 0.80 (m, 2H, C<u>H</u>$_2$).

HRMS (ESI) Calc. for $C_{14}H_{26}N$ $[M+H]^+$ 208.2065, Found 208.2060.

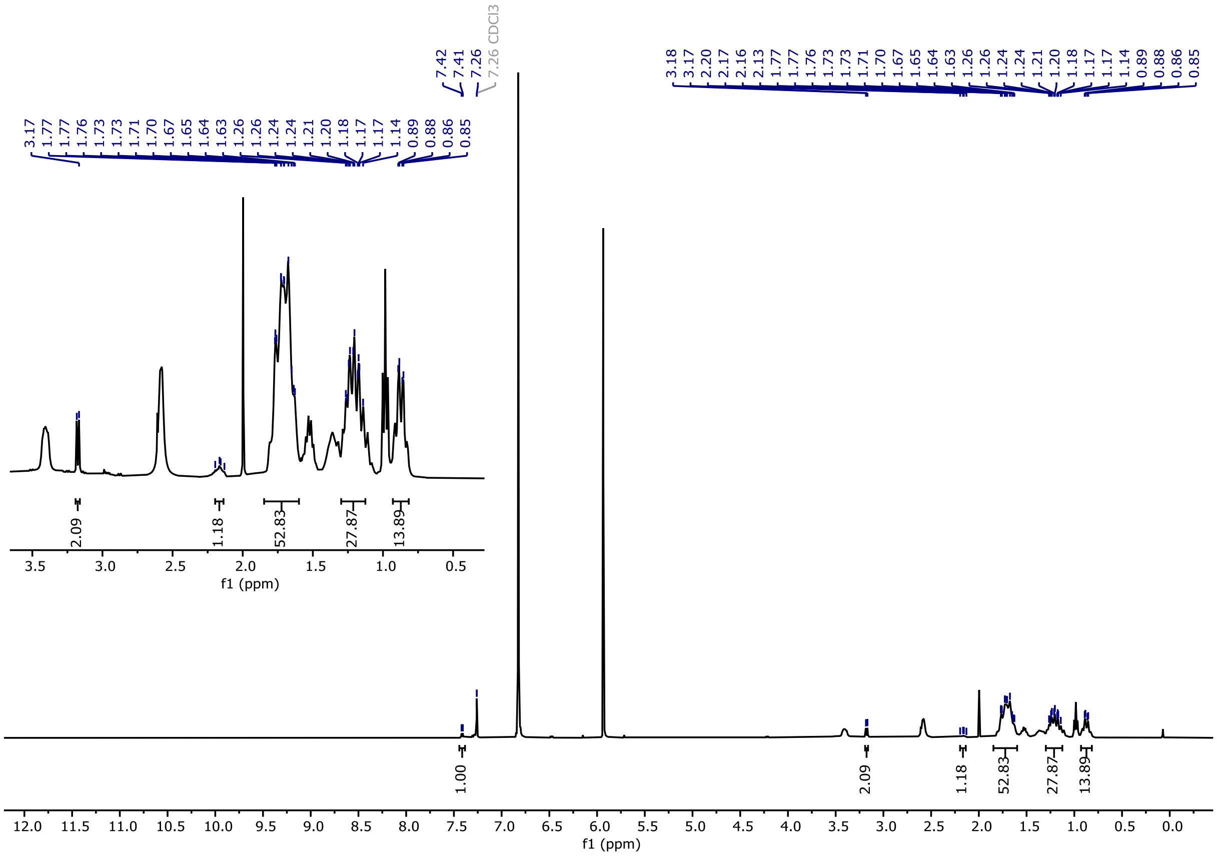

Figure S21. ^{1}H NMR (400 MHz, $CDCl_3$) of **2b**. Multiplets in the range of 3.18 – 0.85 ppm is over integrated due to presence of minor by-products and residual starting material.

***N*-(4-(*tert*-Butyl)benzyl)-1-(4-(*tert*-butyl)phenyl)methanimine (2c)**

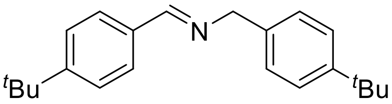

General procedure C was followed using (4-(*tert*-butyl)phenyl)methanamine (**1c**, 35 µL, 0.2 mmol) to produce *N*-(4-(*tert*-butyl)benzyl)-1-(4-(*tert*-butyl)phenyl)methanimine (**2c**) in 44% NMR yield, while in the absence of hybrid POM catalyst imine **2c** was prepared in 10% NMR yield as determined by ^{1}H NMR spectroscopy. The spectral data were in accordance with those reported in the literature.[15]

^{1}H NMR (400 MHz, $CDCl_3$) δ 8.38 (s, 1H, C<u>H</u>), 7.74 (d, J = 8.4 Hz, 2H, ArC<u>H</u>), 7.45 (d, J = 8.4 Hz, 2H, ArC<u>H</u>), 7.37 (d, J = 8.4 Hz, 2H, ArC<u>H</u>), 7.28 (d, J = 8.3 Hz, 2H, ArC<u>H</u>), 4.80 (s, 2H, C<u>H</u>$_2$), 1.35 (s, 9H, C<u>H</u>$_3$), 1.33 (s, 9H, C<u>H</u>$_3$).

HRMS (ESI) Calc. for $C_{22}H_{30}N$ $[M+H]^+$ 308.2378, Found 308.2369.

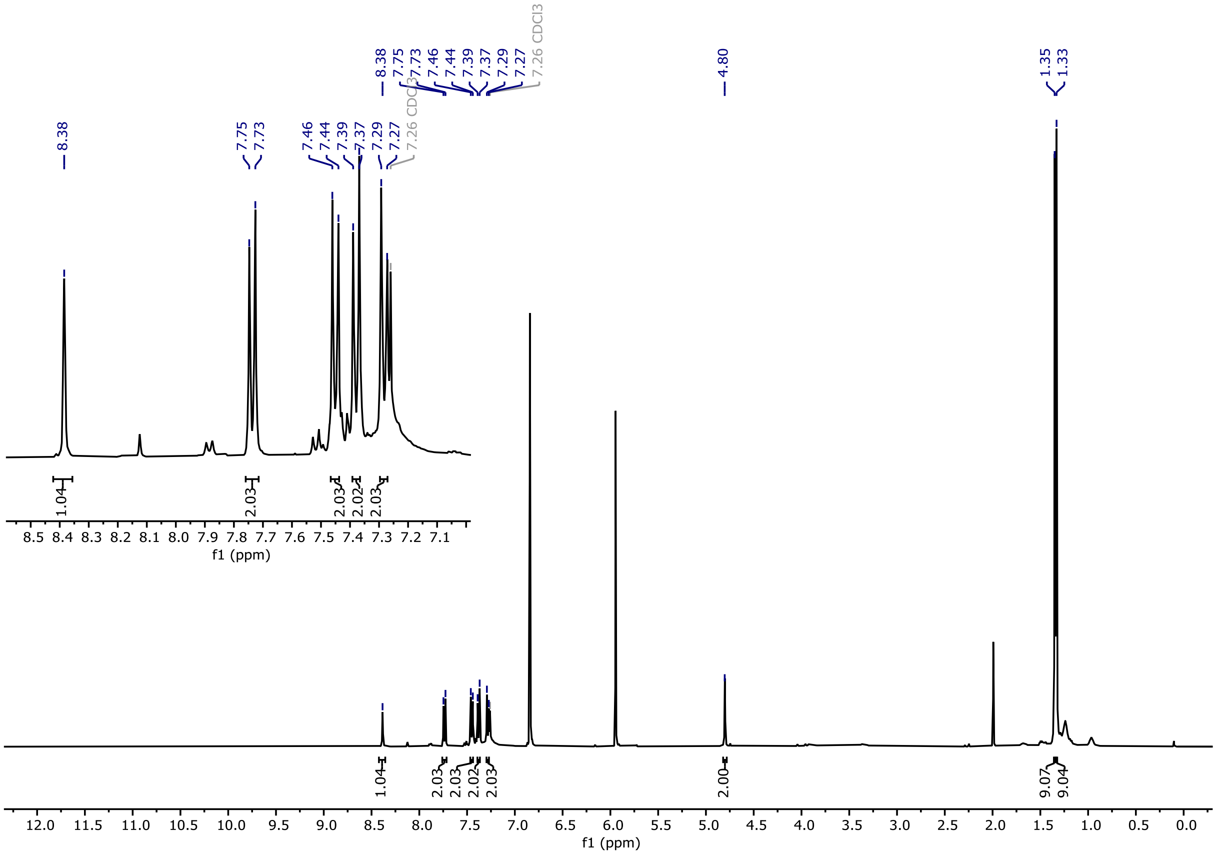

Figure S22. ^{1}H NMR (400 MHz, $CDCl_3$) of **2c**.

N-(4-Methoxybenzyl)-1-(4-methoxyphenyl)methanimine (2d)

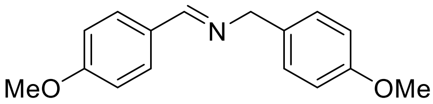

General procedure C was followed using (4-methoxyphenyl)methanamine (**1d**, 26 µL, 0.2 mmol) to produce *N*-(4-methoxybenzyl)-1-(4-methoxyphenyl)methanimine (**2d**) in 46% NMR yield, while in the absence of hybrid POM catalyst imine **2d** was prepared in 40% NMR yield as determined by ^{1}H NMR spectroscopy. The spectral data were in accordance with those reported in the literature.[14]

^{1}H NMR (400 MHz, CDCl$_3$) δ 8.29 (br s, 1H, CH), 7.70 (d, J = 8.8 Hz, 2H, ArCH), 7.24 (d, J = 8.6 Hz, 2H, ArCH), 6.90 (d, J = 8.6 Hz, 2H, ArCH), 6.87 (d, J = 8.6 Hz, 2H, ArCH), 4.71 (s, 2H, CH$_2$), 3.82 (s, 3H, CH$_3$), 3.78 (s, 3H, CH$_3$).

HRMS (ESI) Calc. for $C_{16}H_{18}NO_2$ [M+H]$^+$ 256.1337, Found 256.1334.

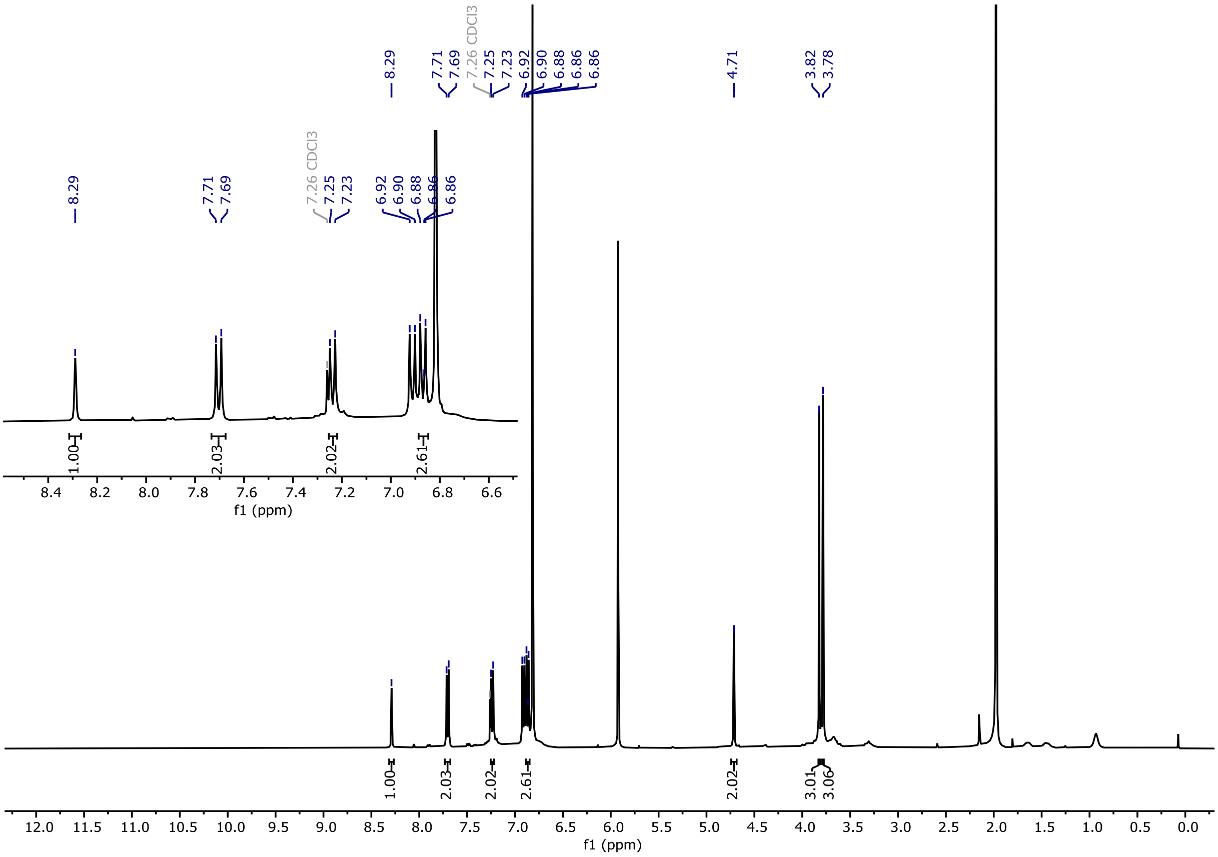

Figure S23. ^{1}H NMR (400 MHz, CDCl$_3$) of **2d**.

N-(4-Fluorobenzyl)-1-(4-fluorophenyl)methanimine (2e)

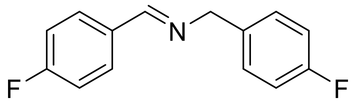

General procedure C was followed using (4-fluorophenyl)methanamine (**1e**, 25 µL, 0.2 mmol) to produce *N*-(4-fluorobenzyl)-1-(4-fluorophenyl)methanimine (**2e**) in 50% NMR yield, while in the absence of hybrid POM catalyst imine **2e** was prepared in 4% NMR yield as determined by ^{1}H NMR spectroscopy. The spectral data were in accordance with those reported in the literature.[16]

^{1}H NMR (400 MHz, $CDCl_3$) δ 8.35 (br s, 1H, CH), 7.79 – 7.76 (m, 2H, ArCH), 7.31 – 7.27 (m, 2H, ArCH), 7.10 (t, J = 8.6 Hz, 2H, ArCH), 7.06 – 7.00 (m, 2H, ArCH), 4.77 (s, 2H, CH_2).

^{19}F NMR (376 MHz, $CDCl_3$) δ −109.16 (tt, J^{H-F} = 8.5, 5.5 Hz), −115.94 (ddd, J^{H-F} = 14.2, 8.9, 5.4 Hz).

HRMS (ESI) Calc. for $C_{14}H_{12}F_2N$ $[M+H]^+$ 232.0938, Found 232.0932.

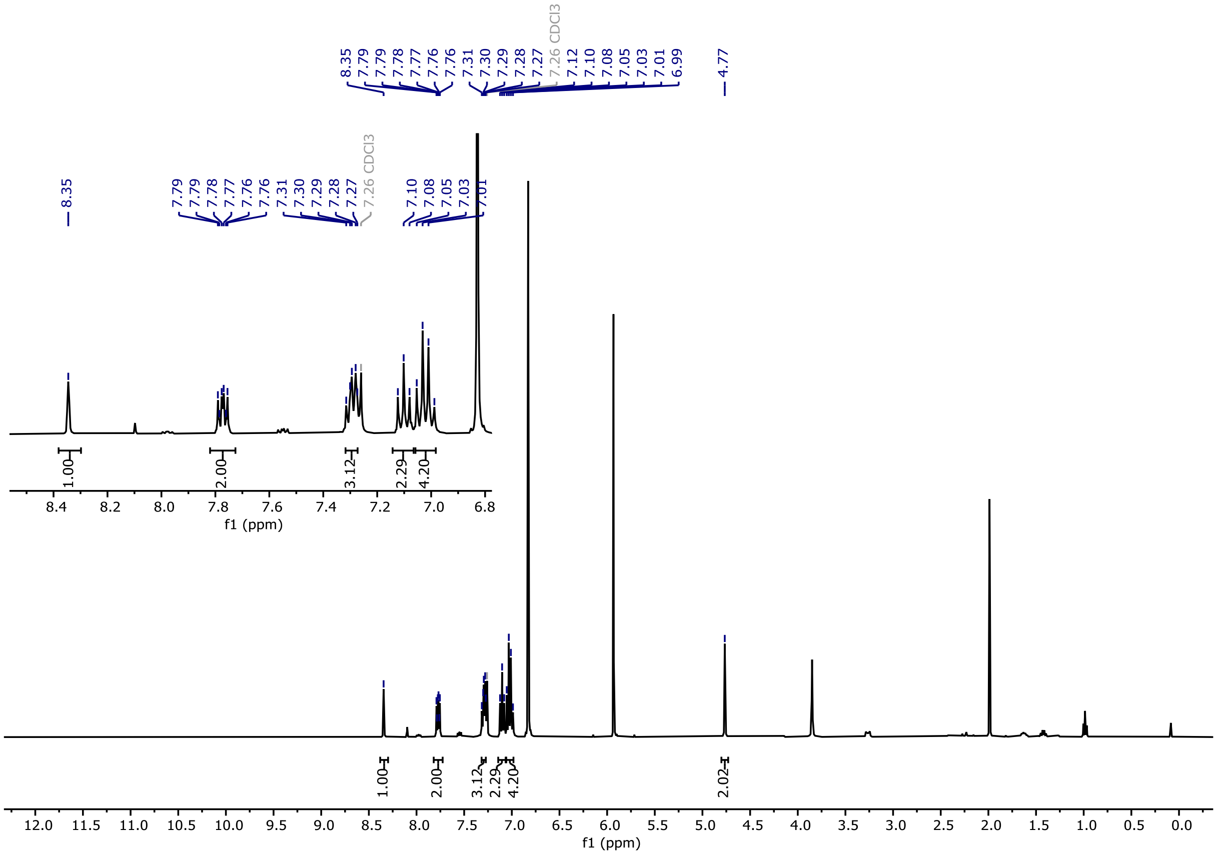

Figure S24. ^{1}H NMR (400 MHz, $CDCl_3$) of **2e**. Multiplets between 7.31 – 7.27 and 7.06 – 7.00 ppm are over integrated due to presence of minor by-products and residual starting material.

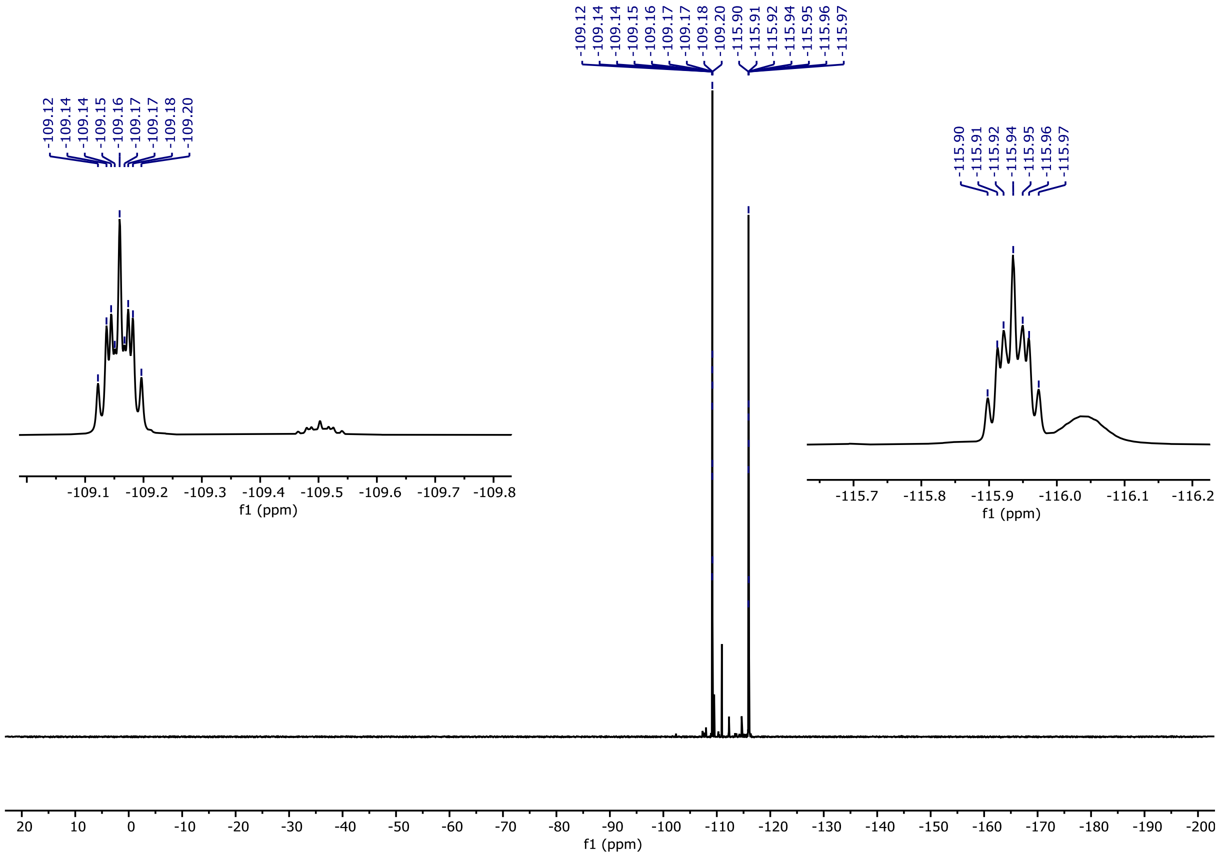

Figure S25. ^{19}F NMR (376 MHz, $CDCl_3$) of **2e**.

N-(3-(Trifluoromethyl)benzyl)-1-(3-(trifluoromethyl)phenyl)methanimine (2f)

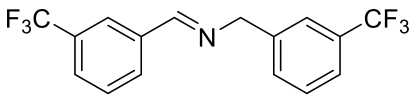

General procedure C was followed using (3-(trifluoromethyl)phenyl)methanamine (**1f**, 35 µL, 0.2 mmol) to produce *N*-(3-(trifluoromethyl)benzyl)-1-(3-(trifluoromethyl)phenyl) methanimine (**2f**) in 50% NMR yield, while in the absence of hybrid POM catalyst imine **2f** was prepared in 16% NMR yield as determined by ^{1}H NMR spectroscopy. The spectral data were in accordance with those reported in the literature.[17]

^{1}H NMR (400 MHz, $CDCl_3$) δ 8.47 (br s, 1H, C<u>H</u>), 8.07 (s, 1H, ArC<u>H</u>), 7.98 (d, *J* = 7.7 Hz, 1H, ArC<u>H</u>), 7.70 (d, *J* = 7.9 Hz, 1H, ArC<u>H</u>), 7.62 (s, 1H, ArC<u>H</u>), 7.58 – 7.53 (m, 3H, ArC<u>H</u>), 7.49 (d, *J* = 7.7 Hz, 1H, ArC<u>H</u>), 4.89 (d, *J* = 1.4 Hz, 2H, C<u>H</u>$_2$).

^{19}F NMR (376 MHz, $CDCl_3$) δ −62.56 (s), −62.76 (s).

HRMS (ESI) Calc. for $C_{16}H_{12}F_6N$ $[M+H]^+$ 322.0868, Found 322.0865.

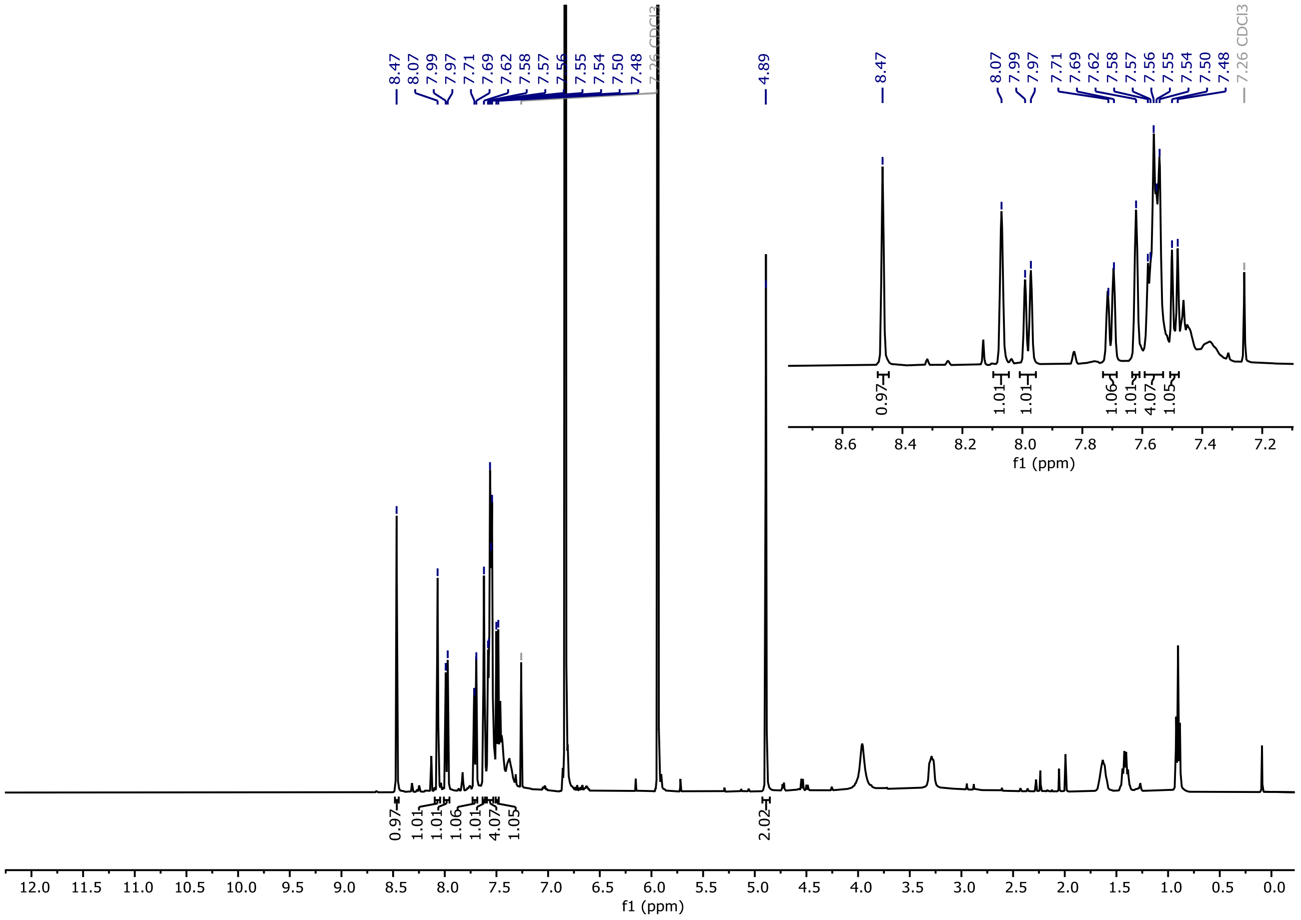

Figure S26. ^{1}H NMR (400 MHz, $CDCl_3$) of **2f**. Multiplet 7.58 – 7.53 ppm is over integrated due to presence of minor by-products and residual starting material.

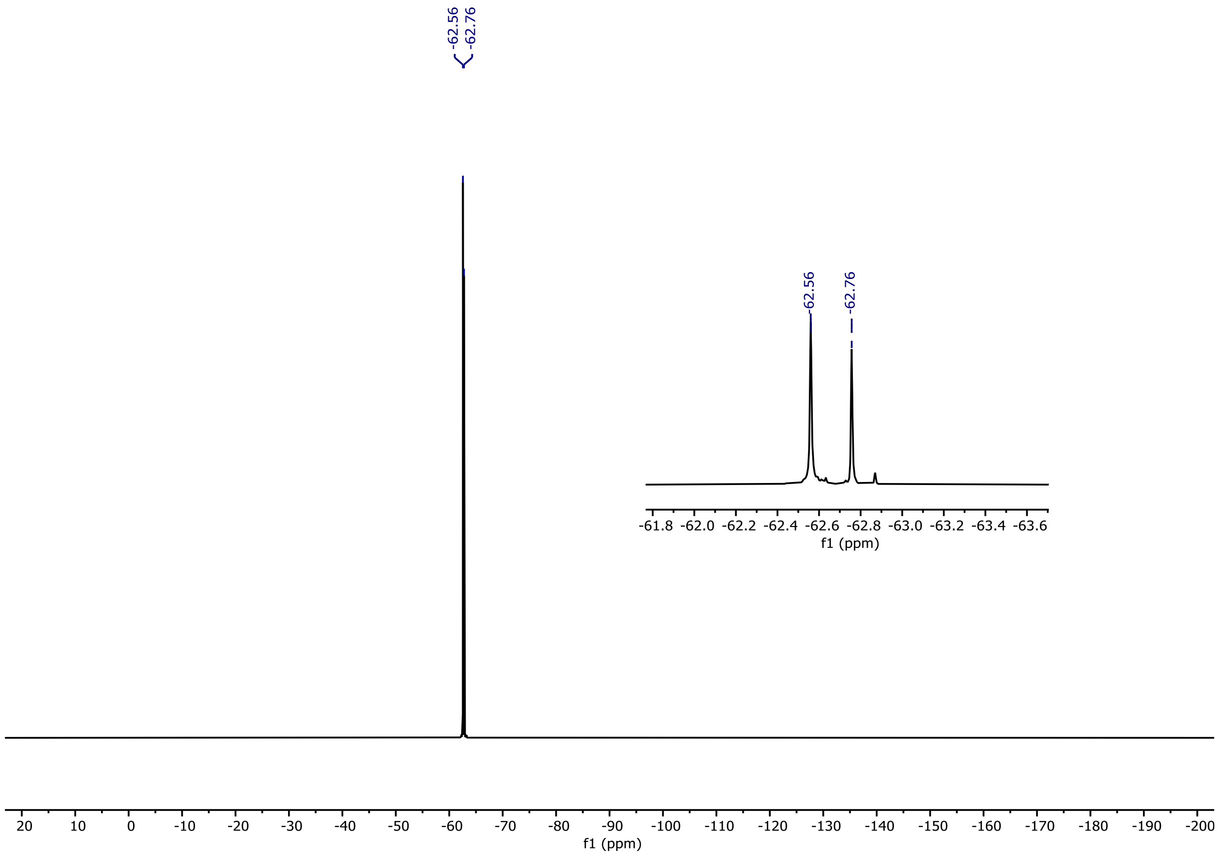

Figure S27. ^{19}F NMR (376 MHz, $CDCl_3$) of **2f**.

_N_-(3-Methylbenzyl)-1-(_m_-tolyl)methanimine (2g)

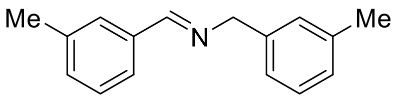

General procedure C was followed using 3-methylbenzylamine (**1g**, 24 µL, 0.2 mmol) to produce *N*-(3-methylbenzyl)-1-(*m*-tolyl)methanimine (**2g**) in 62% NMR yield, while in the absence of hybrid POM catalyst imine **2g** was prepared in 13% NMR yield as determined by ^{1}H NMR spectroscopy. The spectral data were in accordance with those reported in the literature.[14]

^{1}H NMR (400 MHz, $CDCl_3$) δ 8.37 (br s, 1H, C<u>H</u>), 7.66 (s, 1H, ArC<u>H</u>), 7.55 (d, *J* = 7.5 Hz, 1H, ArC<u>H</u>), 7.32 (t, *J* = 7.6 Hz, 1H, ArC<u>H</u>), 7.26 – 7.22 (m, 2H, ArC<u>H</u>), 7.16 – 7.12 (m, 2H, ArC<u>H</u>), 7.08 (d, *J* = 7.6 Hz, 1H, ArC<u>H</u>), 4.79 (s, 2H, C<u>H</u>$_2$), 2.39 (s, 3H, C<u>H</u>$_3$), 2.36 (s, 3H, C<u>H</u>$_3$).

HRMS (ESI) Calc. for $C_{16}H_{18}N$ $[M+H]^+$ 224.1434, Found 224.1433.

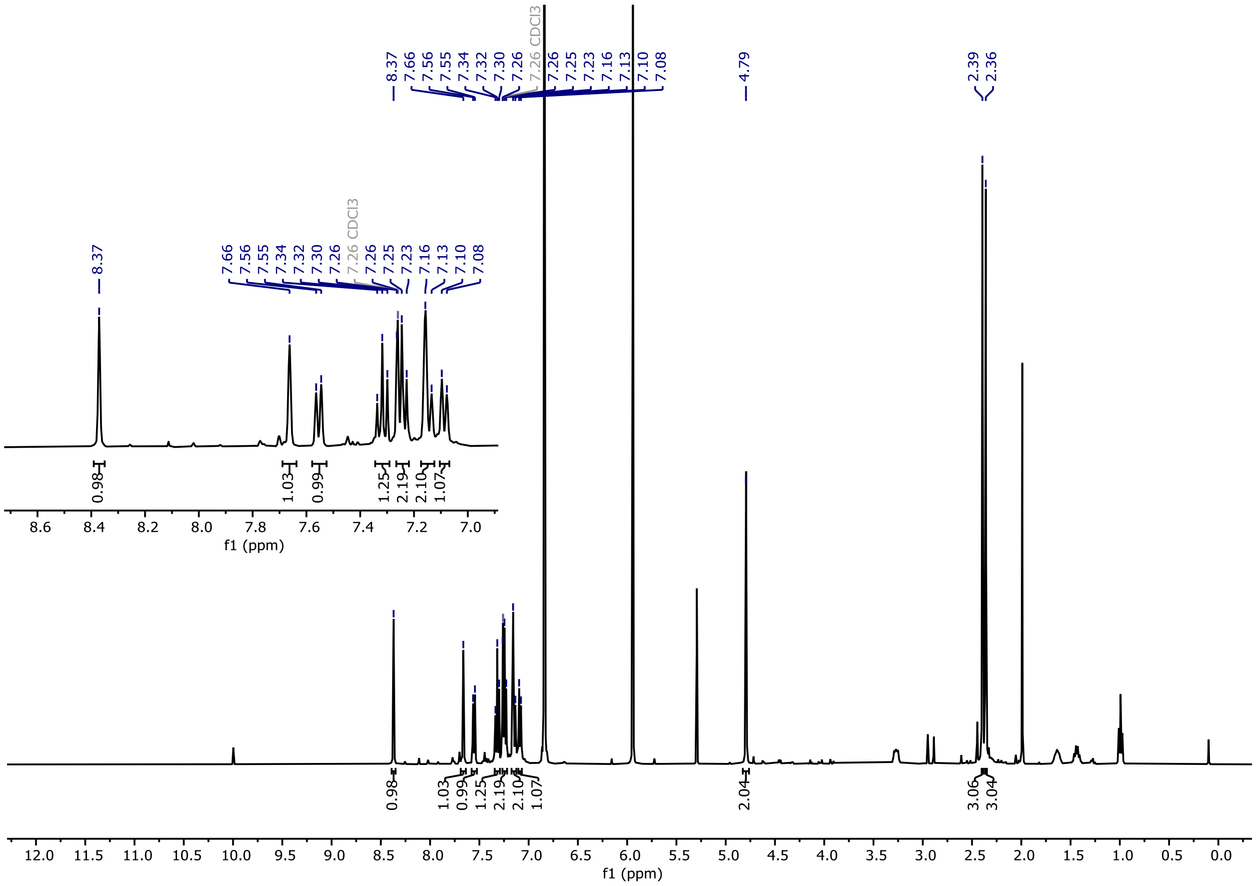

Figure S28. ^{1}H NMR (400 MHz, $CDCl_3$) of **2g**.

1-([1,1'-Biphenyl]-3-yl)-*N*-([1,1'-biphenyl]-3-ylmethyl)methanimine (2h)

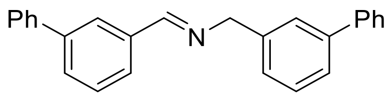

General procedure C was followed using 3-phenylbenzylamine (**1h**, 37 µL, 0.2 mmol) to produce 1-([1,1'-biphenyl]-3-yl)-*N*-([1,1'-biphenyl]-3-ylmethyl)methanimine (**2h**) in 50% NMR yield, while in the absence of hybrid POM catalyst imine **2h** was prepared in 34% NMR yield as determined by ^{1}H NMR spectroscopy.

^{1}H NMR (400 MHz, $CDCl_3$) δ 8.52 (s, 1H, C<u>H</u>), 8.07 (br t, J = 1.8 Hz, 1H, ArC<u>H</u>), 7.79 (br d, J = 7.7 Hz, 1H, ArC<u>H</u>), 7.70 – 7.33 (m, 16H), 4.94 (s, 2H, C<u>H</u>$_2$).

HRMS (ESI) Calc. for $C_{26}H_{22}N$ $[M+H]^+$ 348.1747, Found 348.1752.

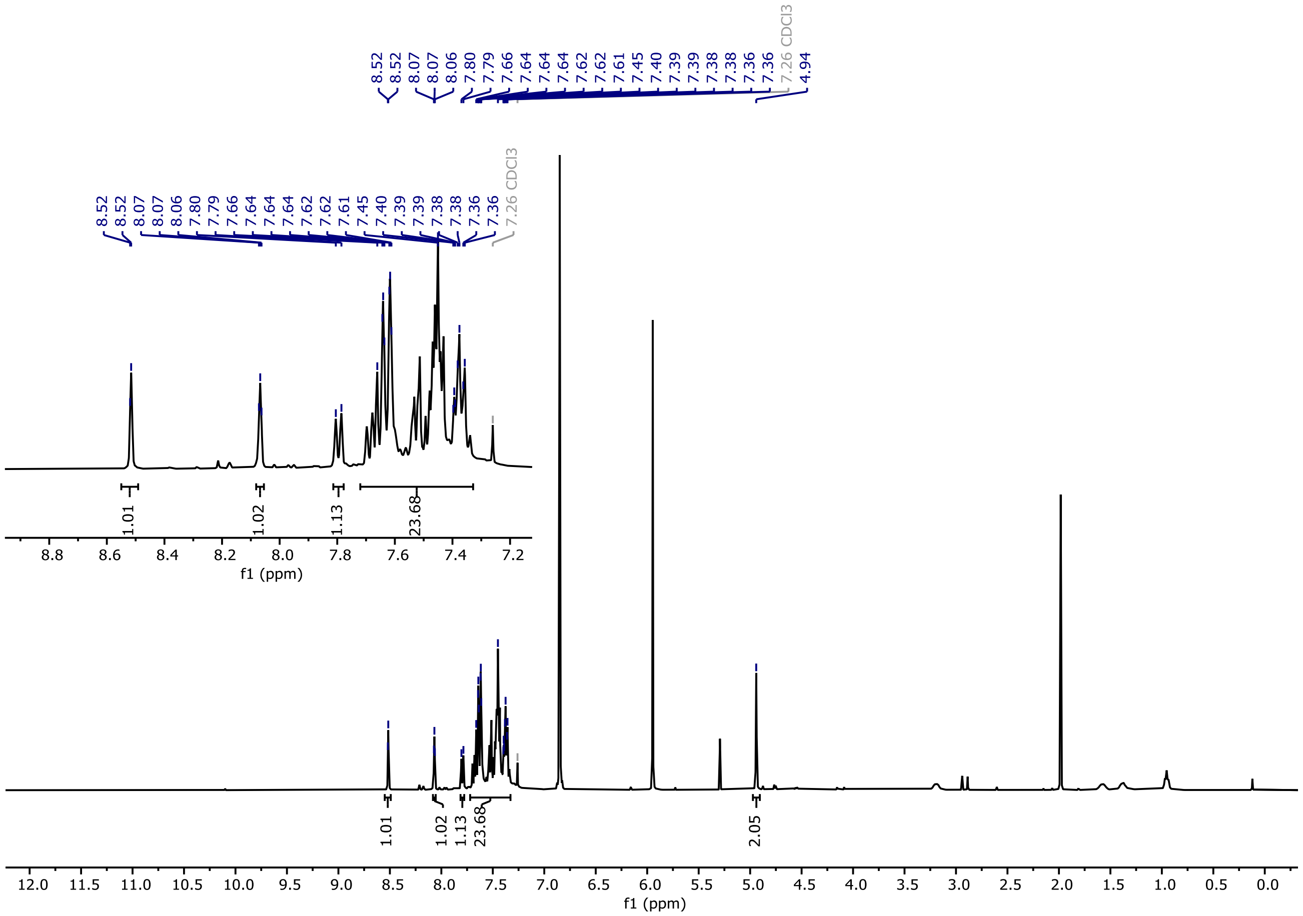

Figure S29. ^{1}H NMR (400 MHz, $CDCl_3$) of **2h**. Multiplet 7.70 – 7.33 ppm is over integrated due to presence of minor by-products and residual starting material.

N-(2-Chlorobenzyl)-1-(2-chlorophenyl)methanimine (2i)

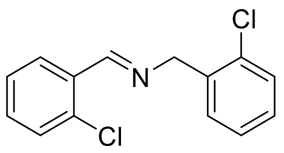

General procedure C was followed using (2-chlorophenyl)methanamine (**1i**, 24 µL, 0.2 mmol) to produce *N*-(2-chlorobenzyl)-1-(2-chlorophenyl)methanimine (**2i**) in 44% NMR yield, while in the absence of hybrid POM catalyst imine **2i** was prepared in 6% NMR yield as determined by ^{1}H NMR spectroscopy. The spectral data were in accordance with those reported in the literature.[14]

^{1}H NMR (400 MHz, $CDCl_3$) *δ* 8.88 (s, 1H, C<u>H</u>), 8.13 (dd, *J* = 7.7, 1.8 Hz, 1H, ArC<u>H</u>), 7.44 - 7.20 (m, 7H, ArC<u>H</u>), 4.95 (s, 2H, C<u>H</u>$_2$).

HRMS (ESI) Calc. for $C_{14}H_{11}Cl_2N$ $[M+H]^+$ 264.0347, Found 264.0335.

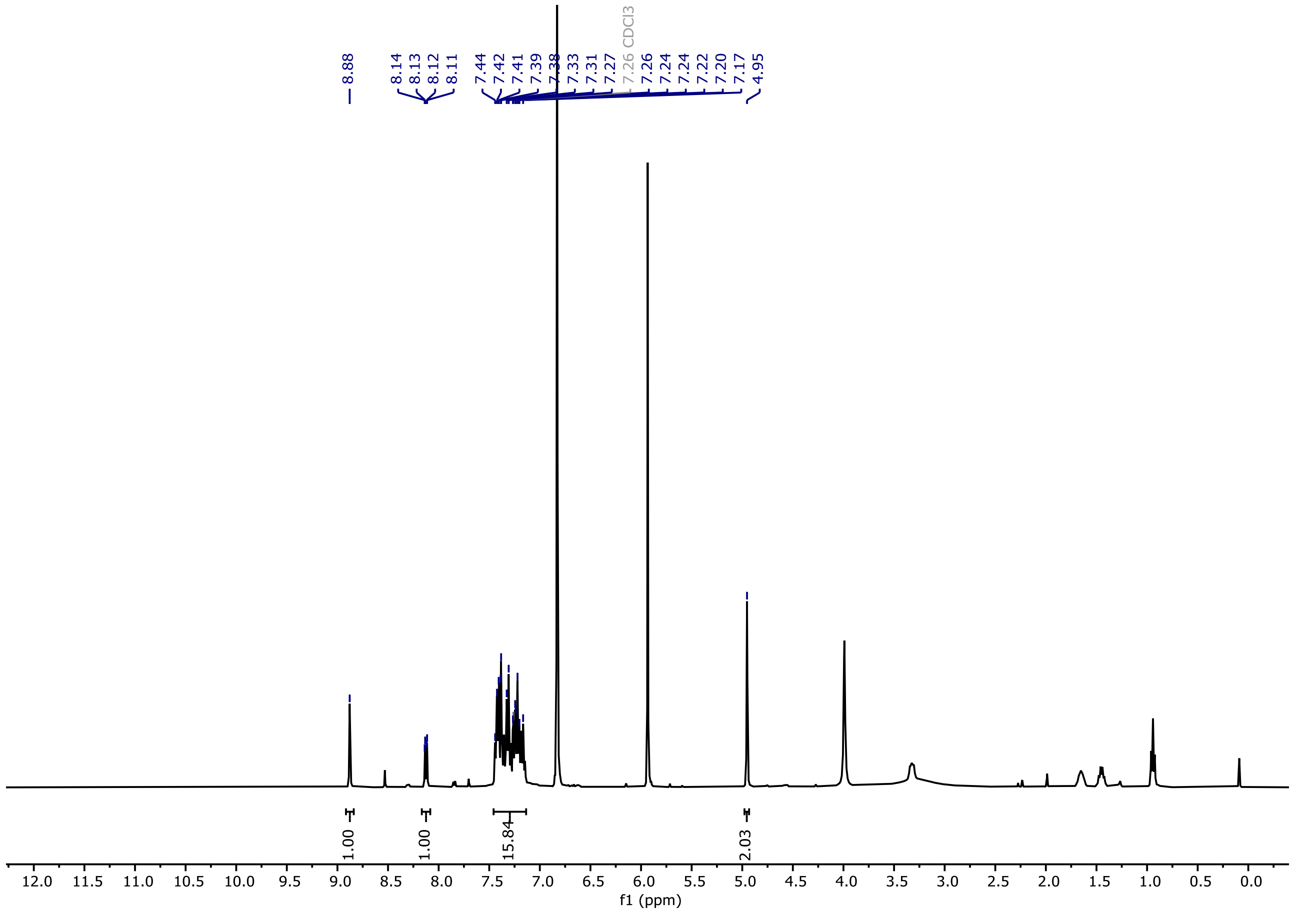

Figure S30. ^{1}H NMR (400 MHz, $CDCl_3$) of **2i**. Multiplet 7.44 – 7.20 ppm is over integrated due to presence of minor by-products and residual starting material, which also masks the true multiplicity of region.

N-(2-Methylbenzyl)-1-(*o*-tolyl)methanimine (2j)

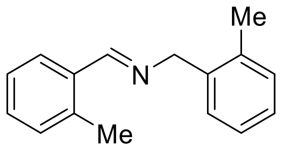

General procedure C was followed using *N*-methyl-1-(o-tolyl)methanamine (**1j**, 24 µL, 0.2 mmol) to produce *N*-(2-methylbenzyl)-1-(*o*-tolyl)methanimine (**2j**) in 50% NMR yield, while in the absence of hybrid POM catalyst imine **2j** was prepared in 8% NMR yield as determined by ^{1}H NMR spectroscopy. The spectral data were in accordance with those reported in the literature.[14]

^{1}H NMR (400 MHz, $CDCl_3$) δ 8.69 (br s, 1H, CH), 7.94 (dd, J = 7.6, 1.3 Hz, 1H, ArCH) 7.33 – 7.29 (m, 2H, ArCH), 7.27 – 7.24 (m, 1H, ArCH), 7.21 – 7.18 (m, 4H, ArCH), 4.85 (s, 2H, CH_2), 2.52 (s, 3H, CH_3), 2.41 (s, 3H, CH_3).

HRMS (ESI) Calc. for $C_{16}H_{17}N$ $[M+H]^+$ 224.1434, Found 224.1437.

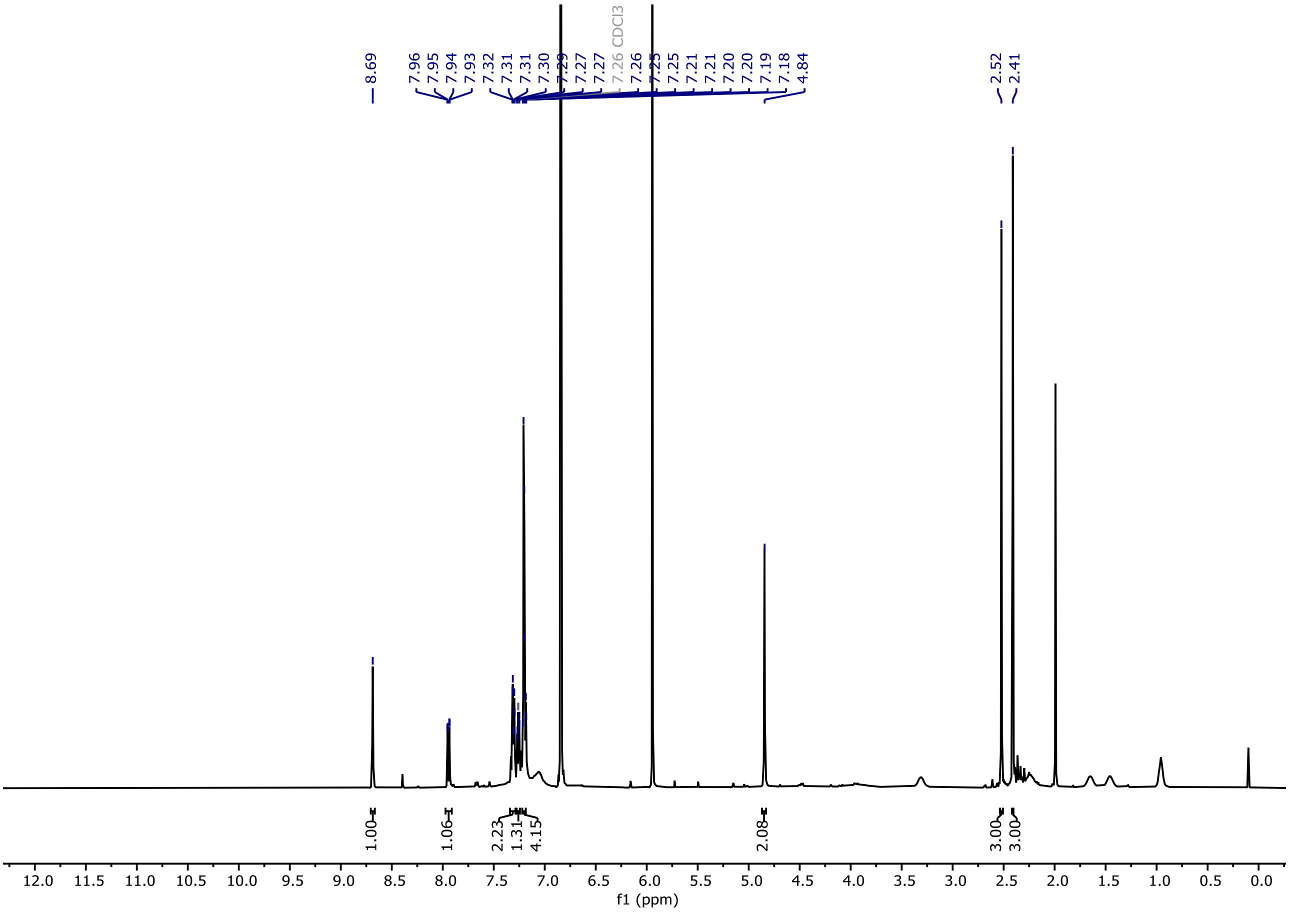

Figure S31. ^{1}H NMR (400 MHz, $CDCl_3$) of **2j**.

N-(3,5-Bis(trifluoromethyl)benzyl)-1-(3,5-bis(trifluoromethyl)phenyl)methanimine (2k)

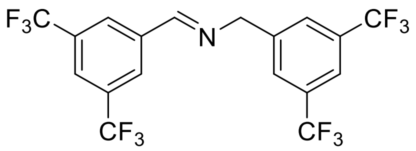

General procedure C was followed using (3,5-bis(trifluoromethyl)phenyl)methanamine (**1k**, 35 µL, 0.2 mmol) to produce *N*-(3,5-bis(trifluoromethyl)benzyl)-1-(3,5-bis(trifluoromethyl) phenyl)methanimine (**2k**) in 46% NMR yield, while in the absence of hybrid POM catalyst imine **2k** was prepared in 2% NMR yield as determined by ^{1}H NMR spectroscopy. The spectral data were in accordance with those reported in the literature.[14]

^{1}H NMR (400 MHz, $CDCl_3$) δ 8.54 (t, *J* = 1.5 Hz, 1H, C<u>H</u>), 8.26 (br s, 2H, ArC<u>H</u>), 7.97 (s, 1H, ArC<u>H</u>), 7.84 – 7.80 (m, 2H, ArC<u>H</u>), 7.74 (s, 1H, ArC<u>H</u>), 4.97 (s, 2H, C<u>H</u>$_2$).

^{19}F NMR (376 MHz, $CDCl_3$) δ −62.88 (s), −63.03 (s).

HRMS (ESI) Calc. for $C_{18}H_{10}F_{12}N$ [M+H]$^+$ 468.0622, Found 468.0615.

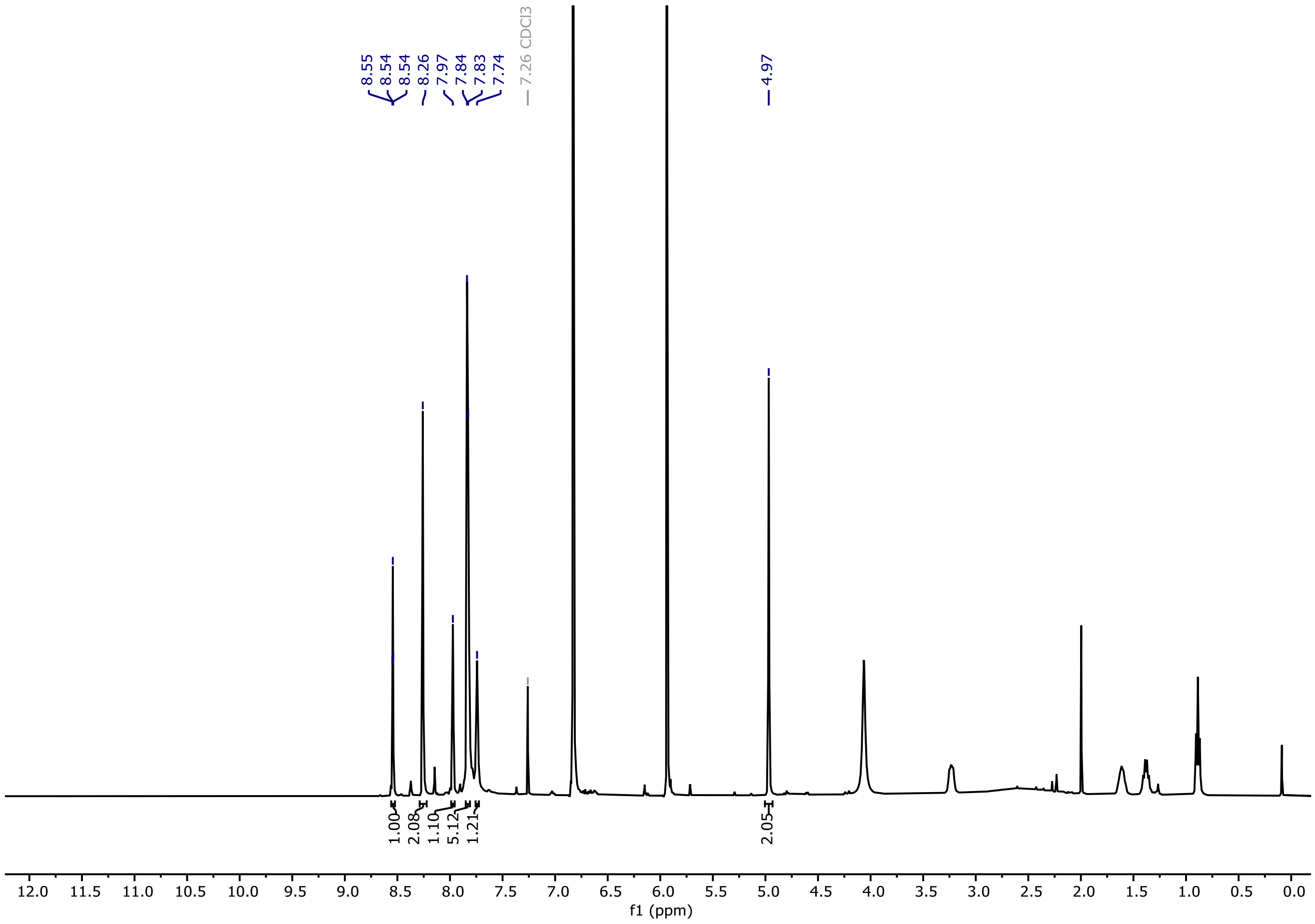

Figure S32. ^{1}H NMR (400 MHz, $CDCl_3$) of **2k**. Multiplet 7.84 – 7.80 ppm is over integrated due to presence of minor by-products and residual starting material.

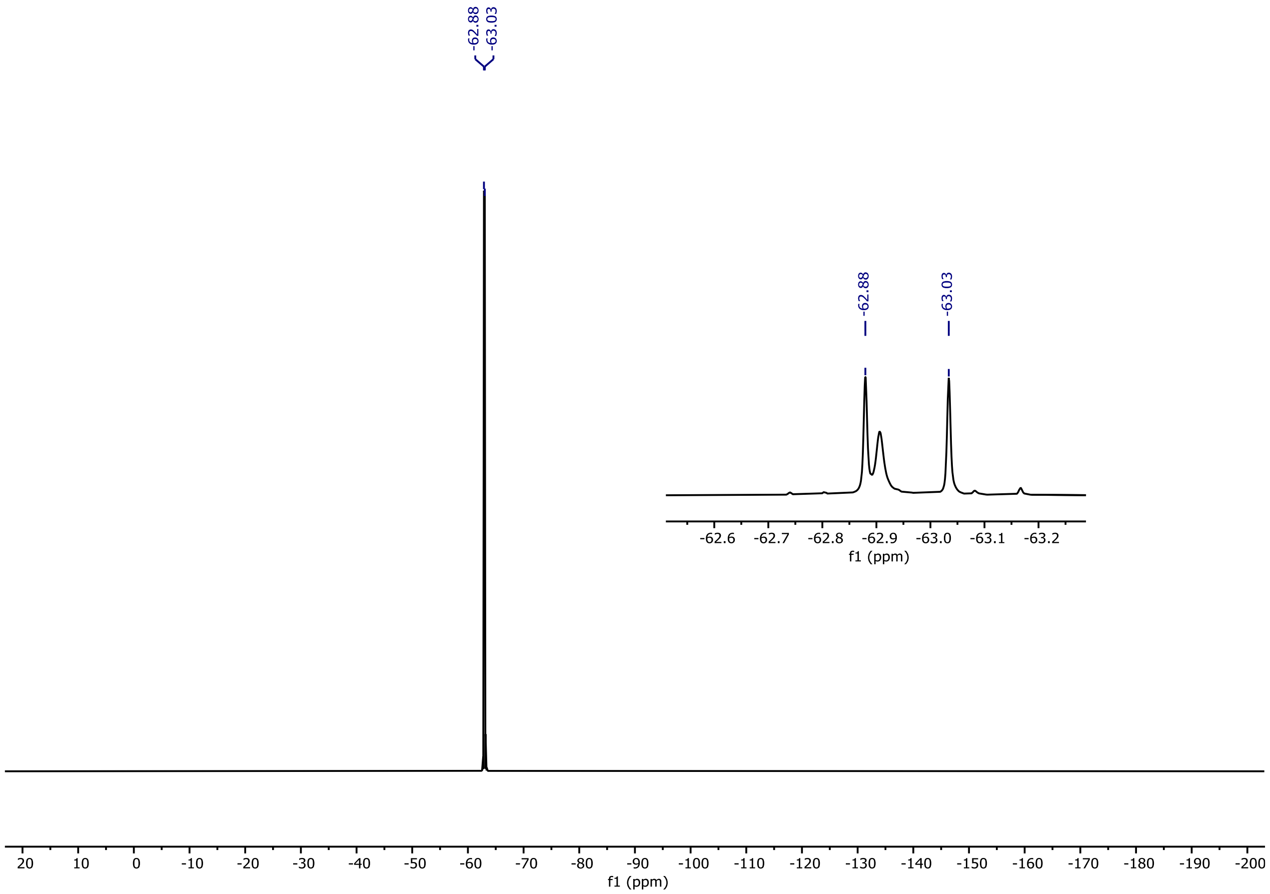

Figure S33. ^{19}F NMR (376 MHz, $CDCl_3$) of **2k**.

1-(Thiophen-2-yl)-*N*-(thiophen-2-ylmethyl)methanimine (2l)

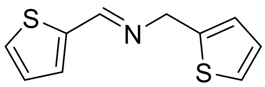

General procedure C was followed using thiophen-2-ylmethanamine (**1l**, 23 µL, 0.2 mmol) to produce 1-(thiophen-2-yl)-*N*-(thiophen-2-ylmethyl)methanimine (**2l**) in 58% NMR yield, while in the absence of hybrid POM catalyst imine **2l** was prepared in 40% NMR yield as determined by ^{1}H NMR spectroscopy. The spectral data were in accordance with those reported in the literature.[14]

^{1}H NMR (400 MHz, $CDCl_3$) δ 8.42 (s, 1H, C<u>H</u>), 7.42 (d, J = 5.0 Hz, 1H, ArC<u>H</u>), 7.33 (d, J = 3.6 Hz, 1H, ArC<u>H</u>), 7.24 (d, J = 4.8 Hz 1H, ArC<u>H</u>), 7.08 (t, J = 4.3 Hz, 1H, ArC<u>H</u>), 7.02 – 6.94 (m, 2H), 4.95 (s, 2H, C<u>H</u>$_2$).

HRMS (ESI) Calc. for $C_{10}H_{10}NS_2$ $[M+H]^+$ 208.0249, Found 208.0243.

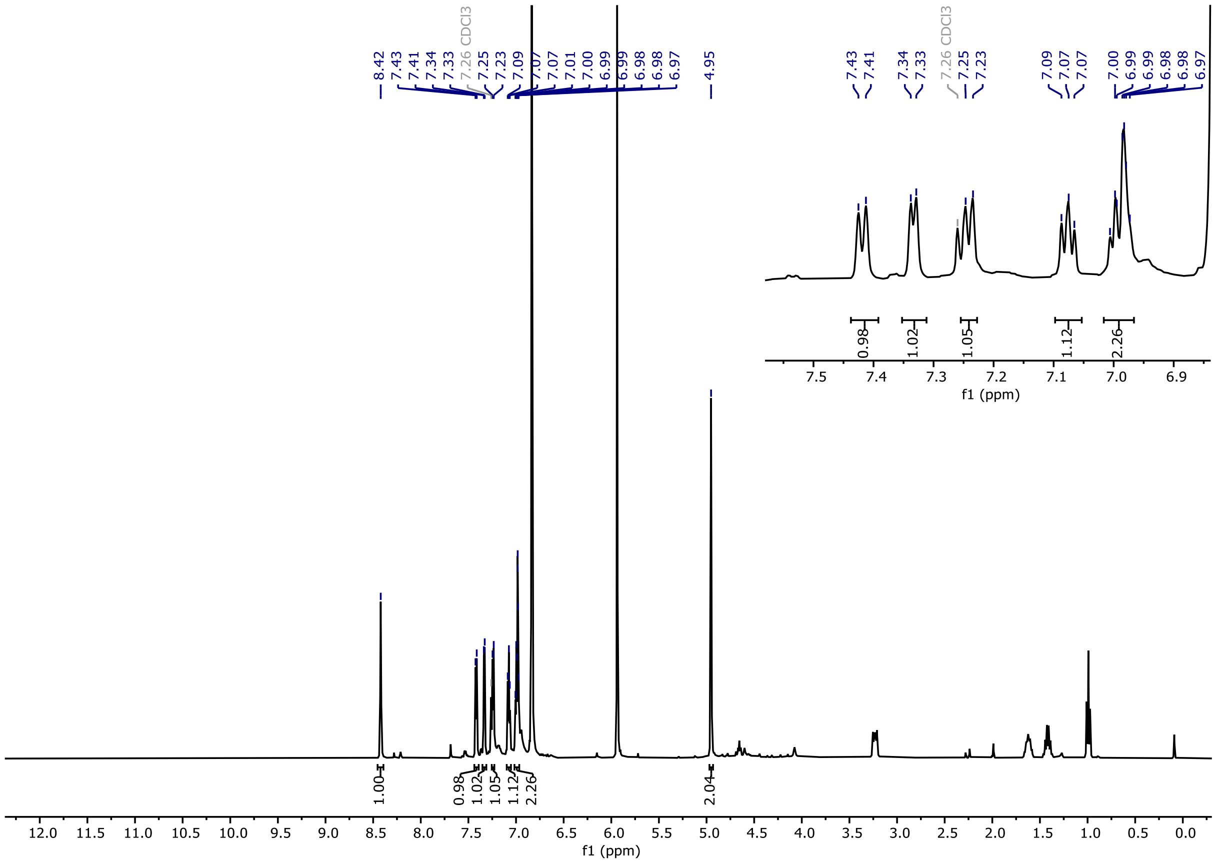

Figure S34. ^{1}H NMR (400 MHz, $CDCl_3$) of **2l**.

5 References

1 C. Rocchiccioli-Deltcheff, M. Fournier, R. Franck and R. Thouvenot, *Inorg. Chem.*, **1983**, 22, 207–216.

2 A. Tézé, M. Michelon and G. Hervé, *Inorg. Chem.*, **1997**, 36, 505–509.

3 G. S. Kim, K. S. Hagen and C. L. Hill, *Inorg. Chem.*, **1992**, 31, 5316–5324.

4 D. R. Allan, H. Nowell, S. A. Barnett, M. R. Warren, A. Wilcox, J. Christensen, L. K. Saunders, A. Peach, M. T. Hooper, L. Zaja, S. Patel, L. Cahill, R. Marshall, S. Trimnell, A. J. Foster, T. Bates, S. Lay, M. A. Williams, P. V. Hathaway, G. Winter, M. Gerstel and R. W. Wooley, *Crystals*, **2017**, 7, 336.

5 J. Cosier and A. M. Glazer, *J. Appl. Crystallogr.*, **1986**, 19, 105–107.

6 Rigaku Oxford Diffraction, 2018, CrysAlisPro Software system, version 1.171.40.45a, Rigaku Corporation, Oxford, UK.

7 G. Winter, D. G. Waterman, J. M. Parkhurst, A. S. Brewster, R. J. Gildea, M. Gerstel, L. Fuentes-Montero, M. Vollmar, T. Michels-Clark, I. D. Young, N. K. Sauter and G. Evans, *Acta Crystallographica Section D*, **2018**, 74, 85–97.

8 Journal, 2018, CCP4 7.0.062 AIMLESS, version 060.067.062 027/005/018.

9 O. V Dolomanov, L. J. Bourhis, R. J. Gildea, J. A. K. Howard and H. Puschmann, *J. Appl. Crystallogr.*, **2009**, 42, 339–341.

10 G. M. Sheldrick, *Acta. Crystallogr. A.*, **2015**, 71, 3–8.

11 "CheckCIF," can be found under http://checkcif.iucr.org.

12 R. I. Maksimovskaya and G. M. Maksimov, *Inorg. Chem.*, **2011**, 50, 4725–4731.

13 L. Pitzer, F. Schäfers and F. Glorius, *Angew. Chem., Int. Ed.*, **2019**, 58, 8572–8576.

14 Y. Goriya, H. Y. Kim and K. Oh, *Org. Lett.*, **2016**, 18, 5174–5177.

15 J. Zhao, H. Sun, Y. Lu, J. Li, Z. Yu, H. Zhu, C. Ma, Q. Meng and X. Peng, *Green Chem.*, **2022**, 24, 8503–8511.

16 P. Galletti, F. Funiciello, R. Soldati and D. Giacomini, *Adv. Synth. Catal.*, **2015**, 357, 1840–1848.

17 I. Kumar, R. Kumar, S. S. Gupta and U. Sharma, *J. Org. Chem.*, **2021**, 86, 6449–6457.

18 P. Zhang, C. Yu, Y. Yin, J. Droste, S. Klabunde, M. R. Hansen, Y. Mai, *Chem. Eur. J.* **2020**, 26, 16497.

6 NMR Spectra

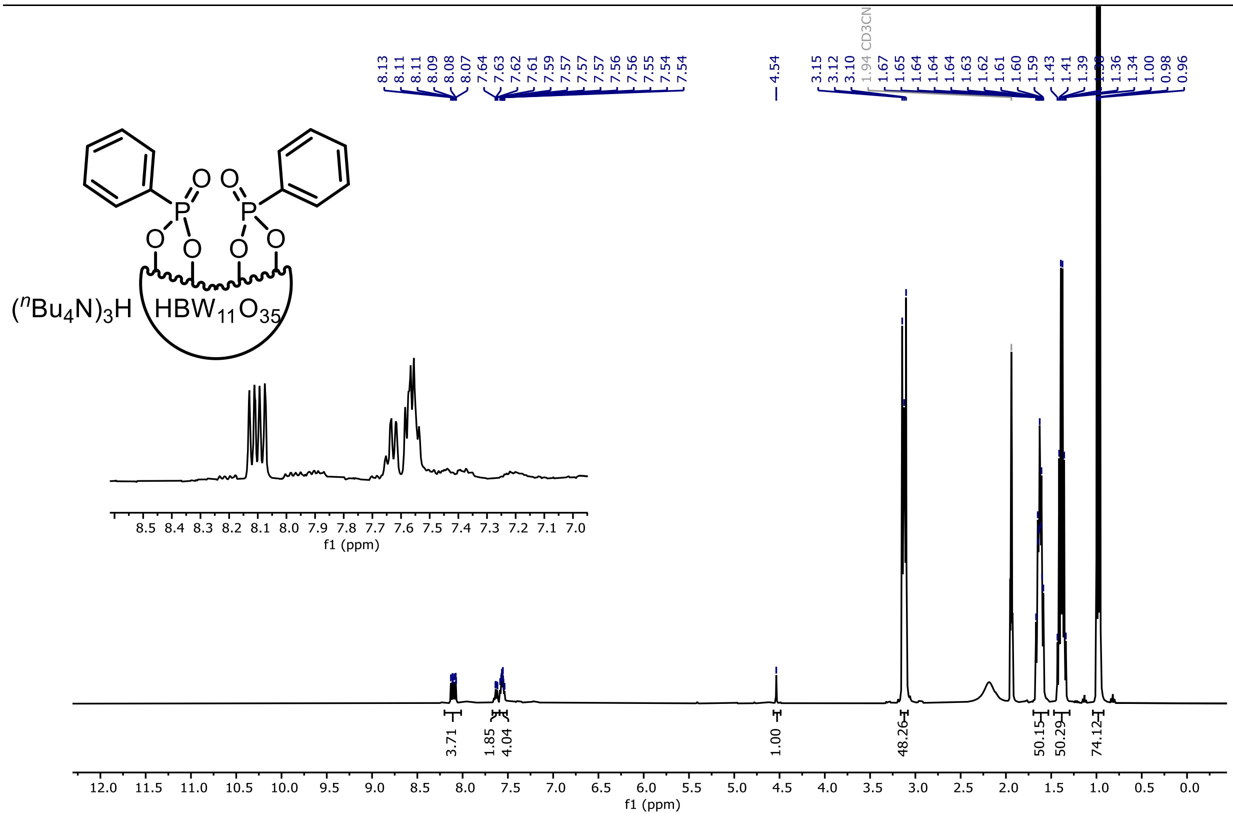

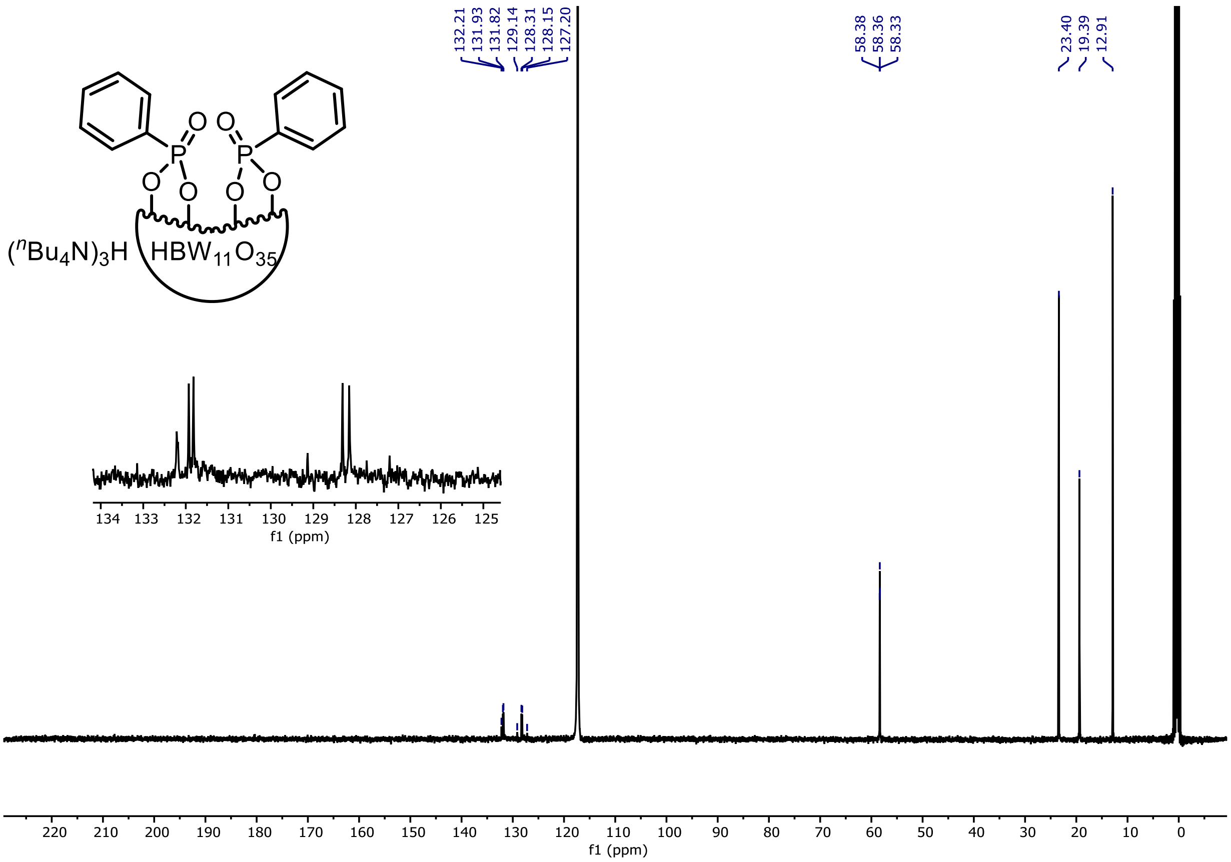

Figure S35. ^{1}H NMR (400 MHz, CD_3CN) and ^{13}C NMR (101 MHz, CD_3CN) of (^{n}Bu$_4$N)$_3$H[HBW$_{11}$O$_{39}$(P(O)Ph)$_2$] (**PBW$_{11}$**).

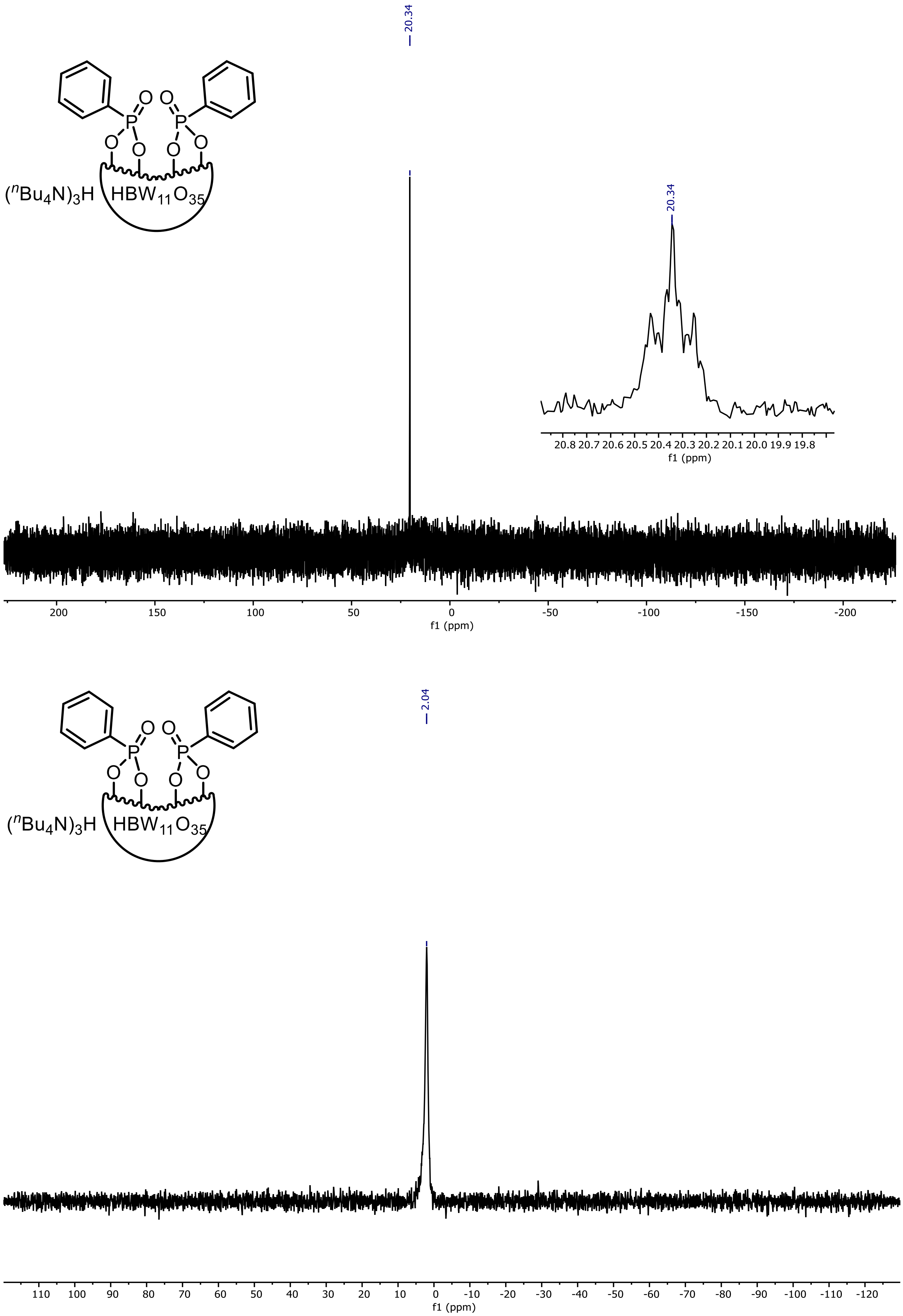

Figure S36. ^{31}P NMR (162 MHz, CD_3CN) and ^{11}B NMR (128 MHz, CD_3CN) of (^{n}Bu$_4$N)$_3$H[HBW$_{11}$O$_{39}$(P(O)Ph)$_2$] (**PBW$_{11}$**).

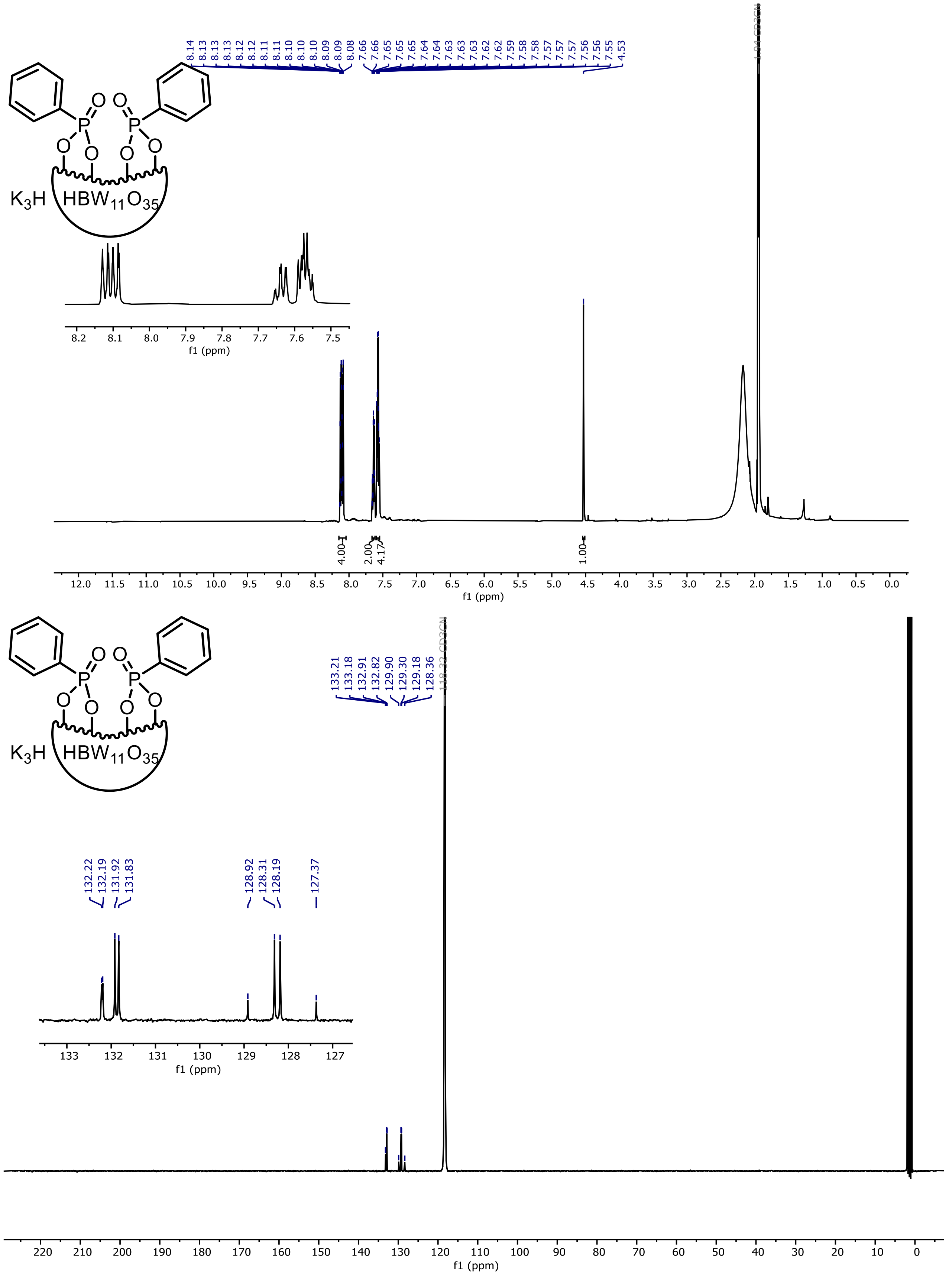

Figure S37. ^{1}H NMR (500 MHz, CD_3CN) and ^{13}C NMR (126 MHz, CD_3CN) of $K_3H[HBW_{11}O_{39}(P(O)Ph)_2]$ (**$KPBW_{11}$**).

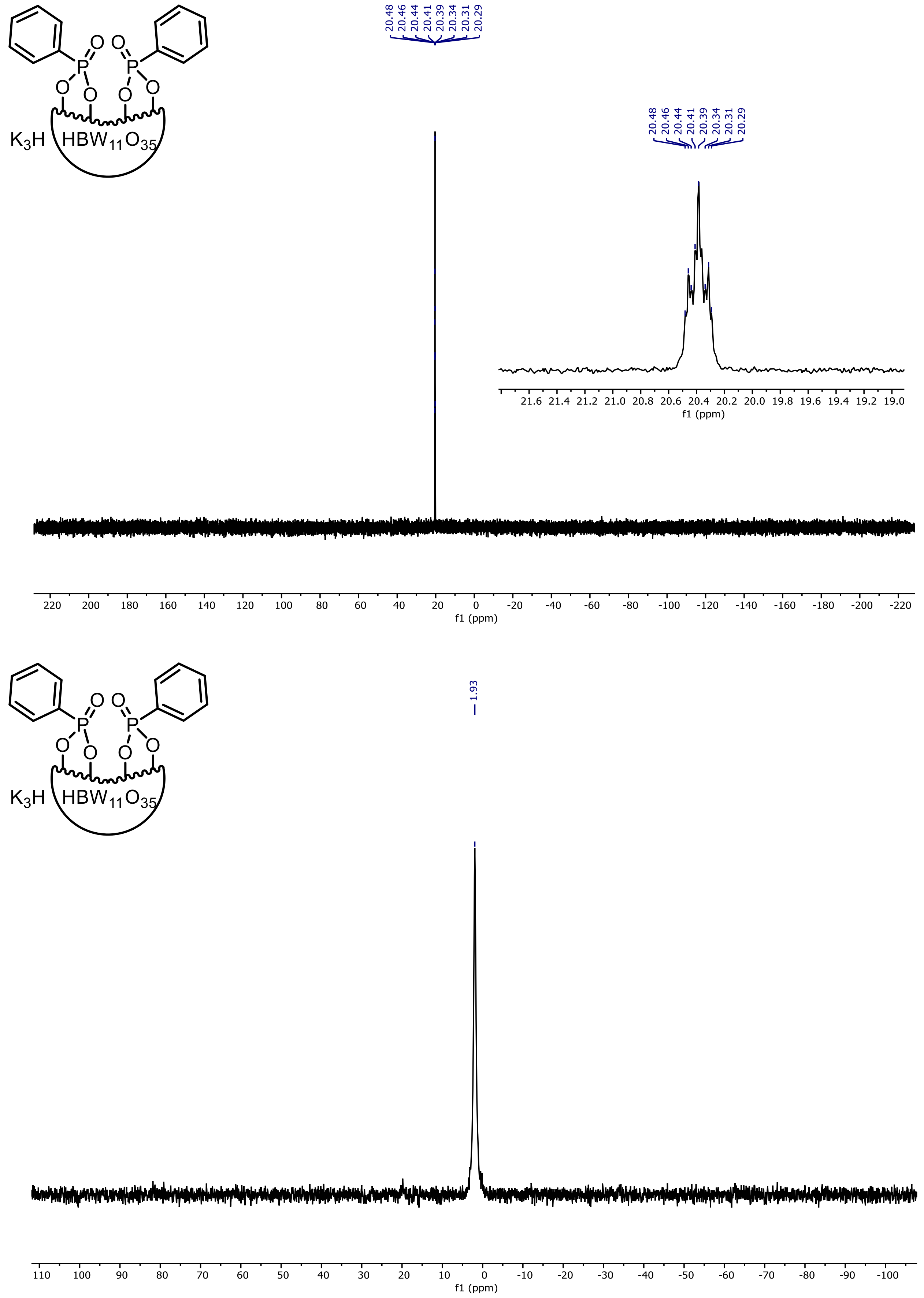

Figure S38. ^{31}P NMR (202 MHz, CD_3CN) and ^{11}B NMR (128 MHz, CD_3CN) of $K_3H[HBW_{11}O_{39}(P(O)Ph)_2]$ (**$KPBW_{11}$**).

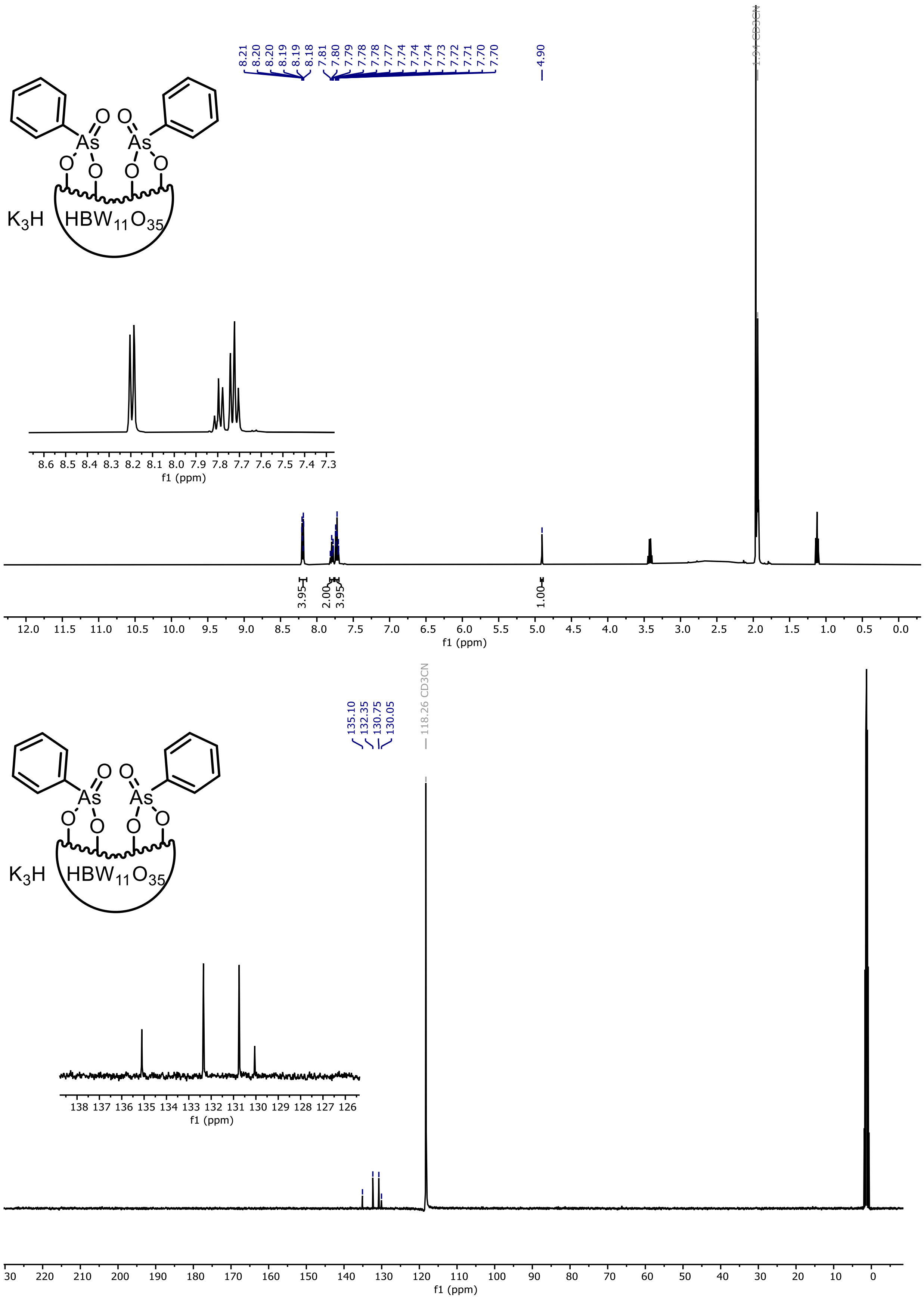

Figure S39. ^{1}H NMR (400 MHz, CD_3CN) and ^{13}C NMR (101 MHz, CD_3CN) of $K_3H[HBW_{11}O_{39}(As(O)Ph)_2]$ (**KAsBW$_{11}$**).

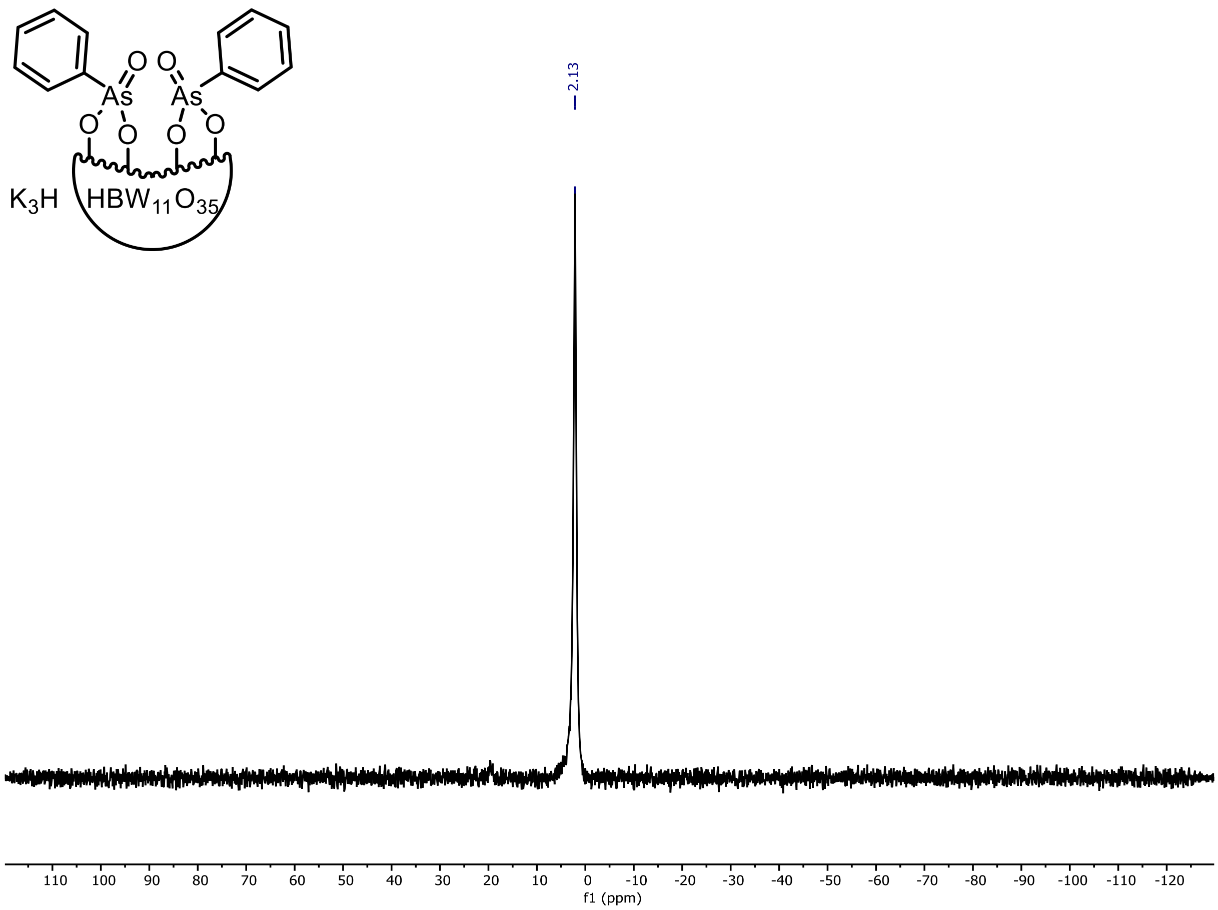

Figure S40. ^{11}B NMR (128 MHz, CD_3CN) of $(^{n}Bu_4N)_3H[HBW_{11}O_{39}(As(O)Ph)_2]$ (**KAsBW$_{11}$**).

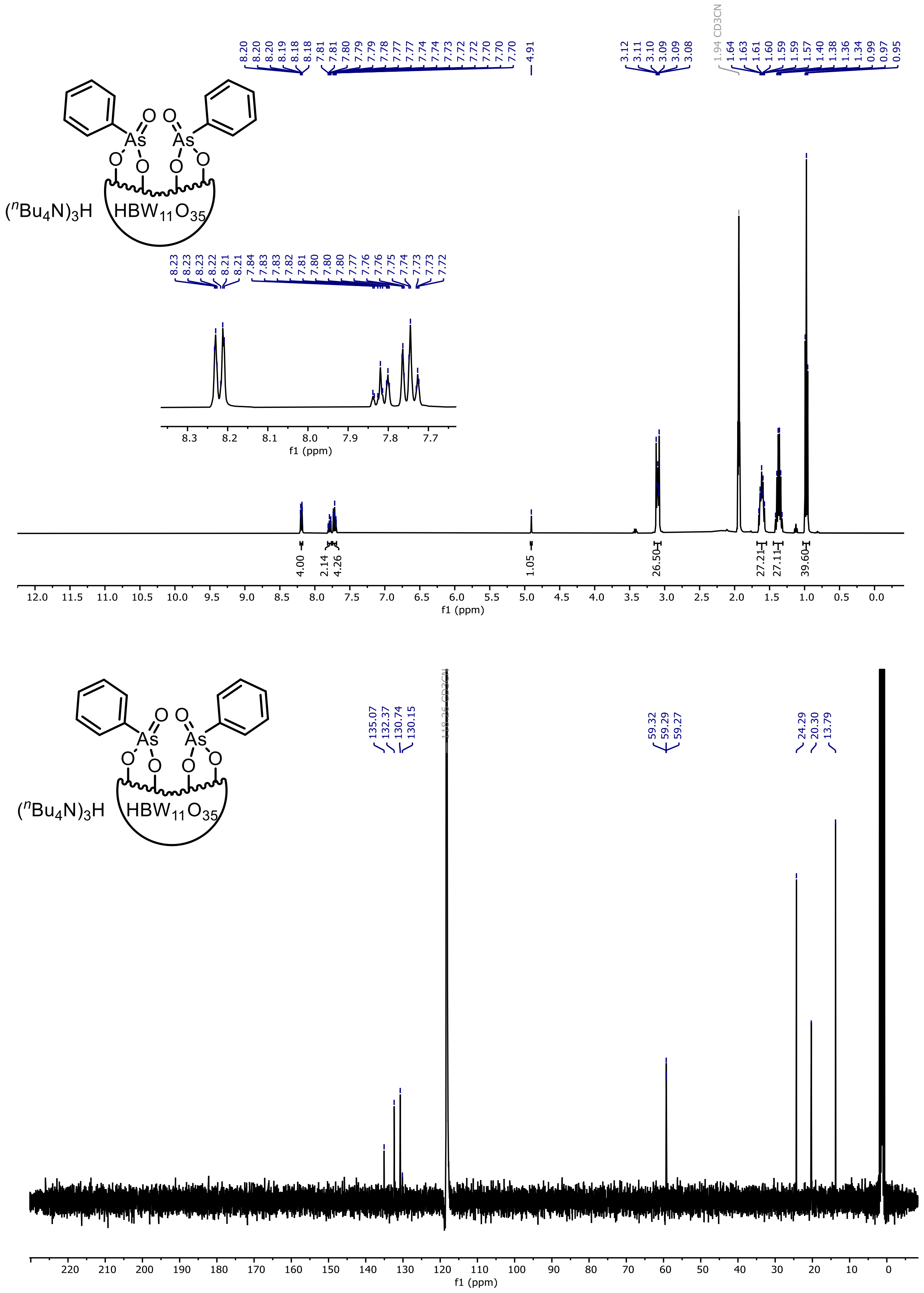

Figure S41. ^{1}H NMR (400 MHz, CD_3CN) and ^{13}C NMR (101 MHz, CD_3CN) of ($^{n}Bu_4N)_3H[HBW_{11}O_{39}(As(O)Ph)_2]$ (**$AsBW_{11}$**).

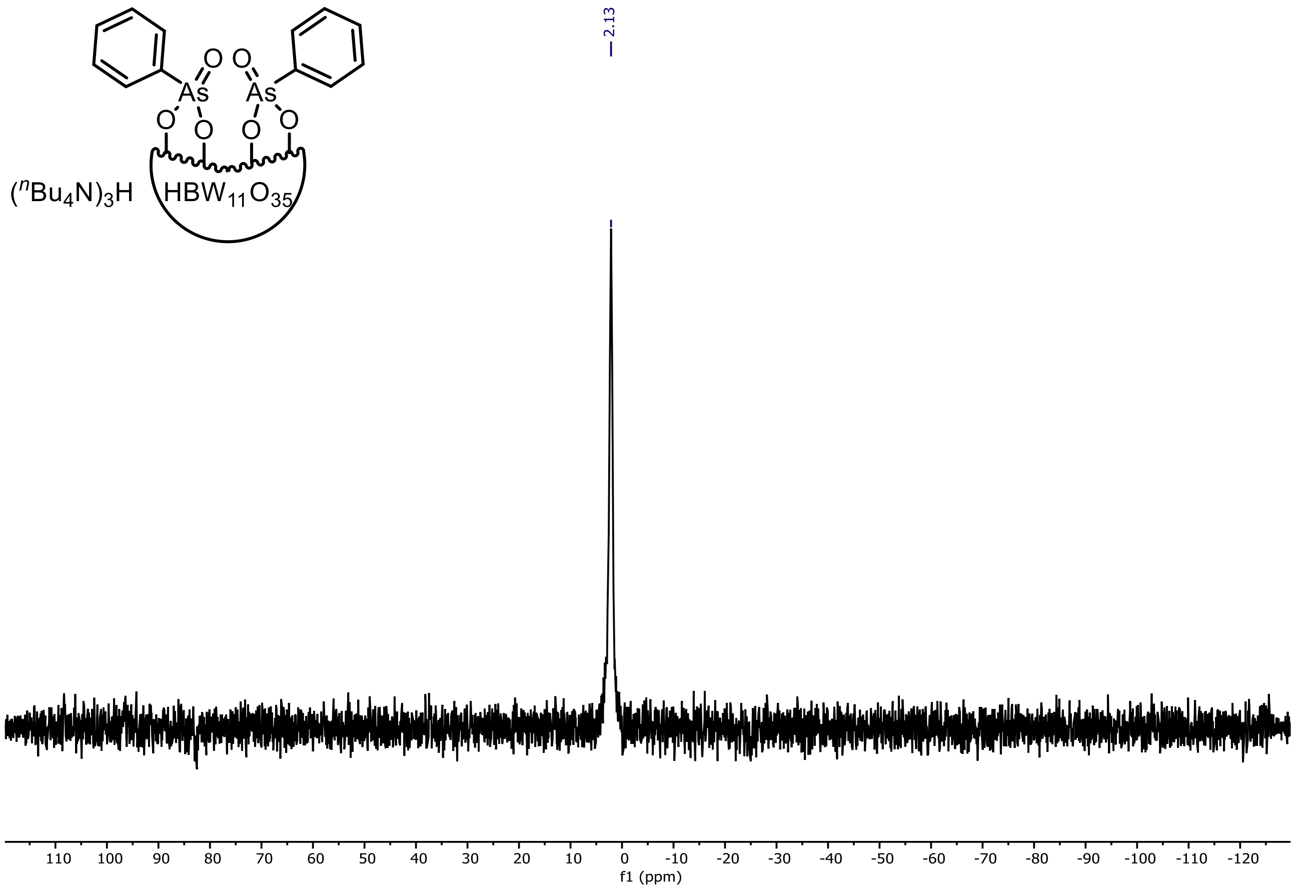

Figure S42. ^{11}B NMR (128 MHz, CD_3CN) of (^{n}Bu$_4$N)$_3$H[HBW$_{11}$O$_{39}$(As(O)Ph)$_2$] (**AsBW$_{11}$**).

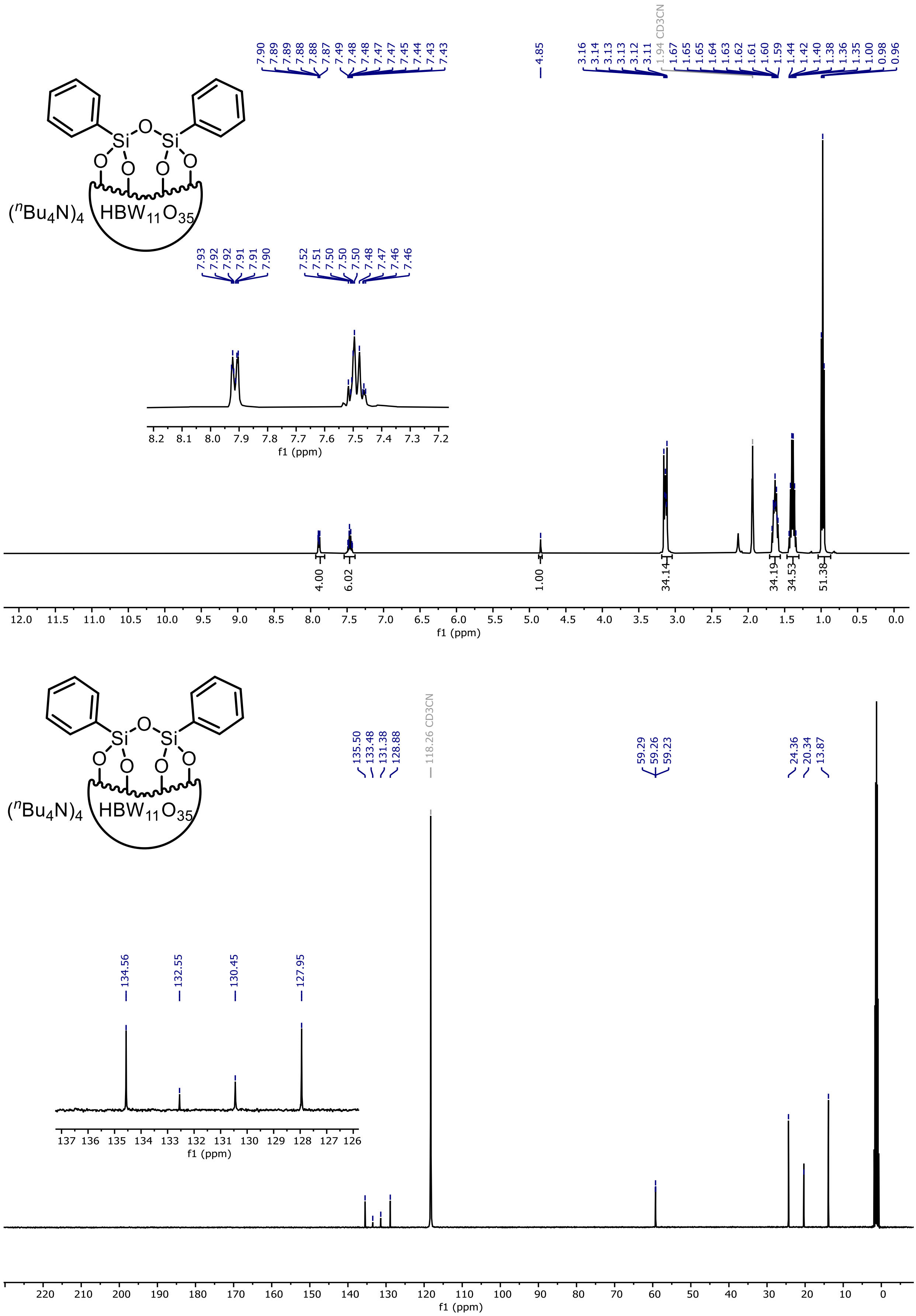

Figure S43. ^{1}H NMR (400 MHz, CD_3CN) and ^{13}C NMR (101 MHz, CD_3CN) of (^{n}Bu$_4$N)$_4$[HBW$_{11}$O$_{39}$(PhSiOSiPh)] (**SiBW$_{11}$**).

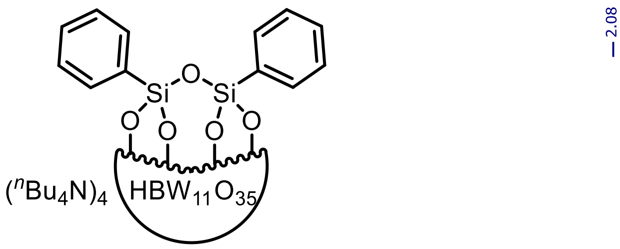

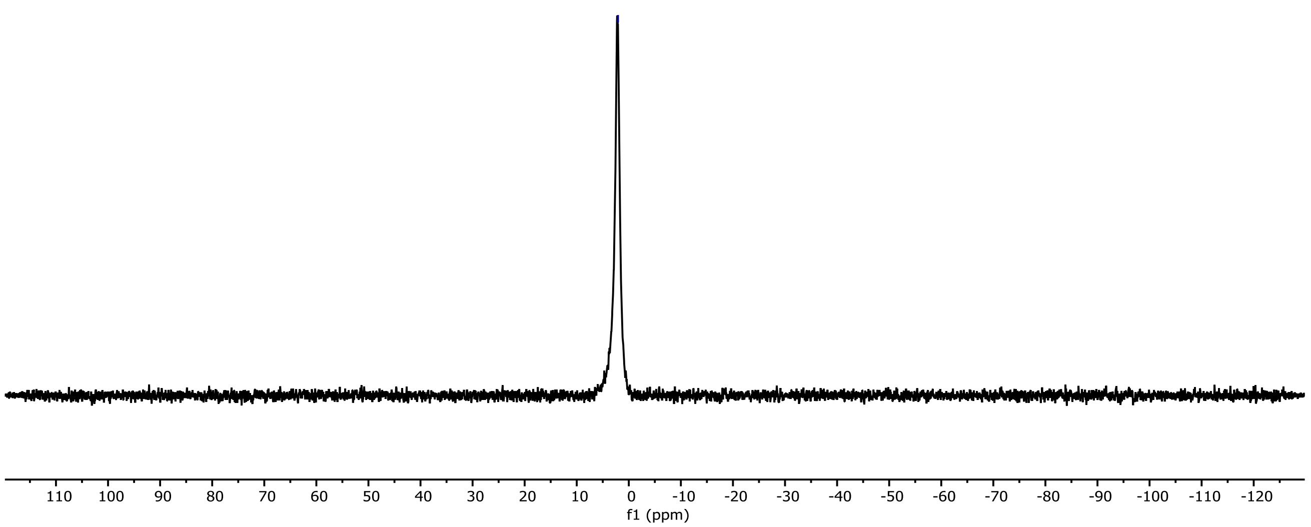

Figure S44. ^{11}B NMR (128 MHz, CD_3CN) of (^{n}Bu$_4$N)$_4$[HBW$_{11}$O$_{39}$(PhSiOSiPh)] (**SiBW$_{11}$**).

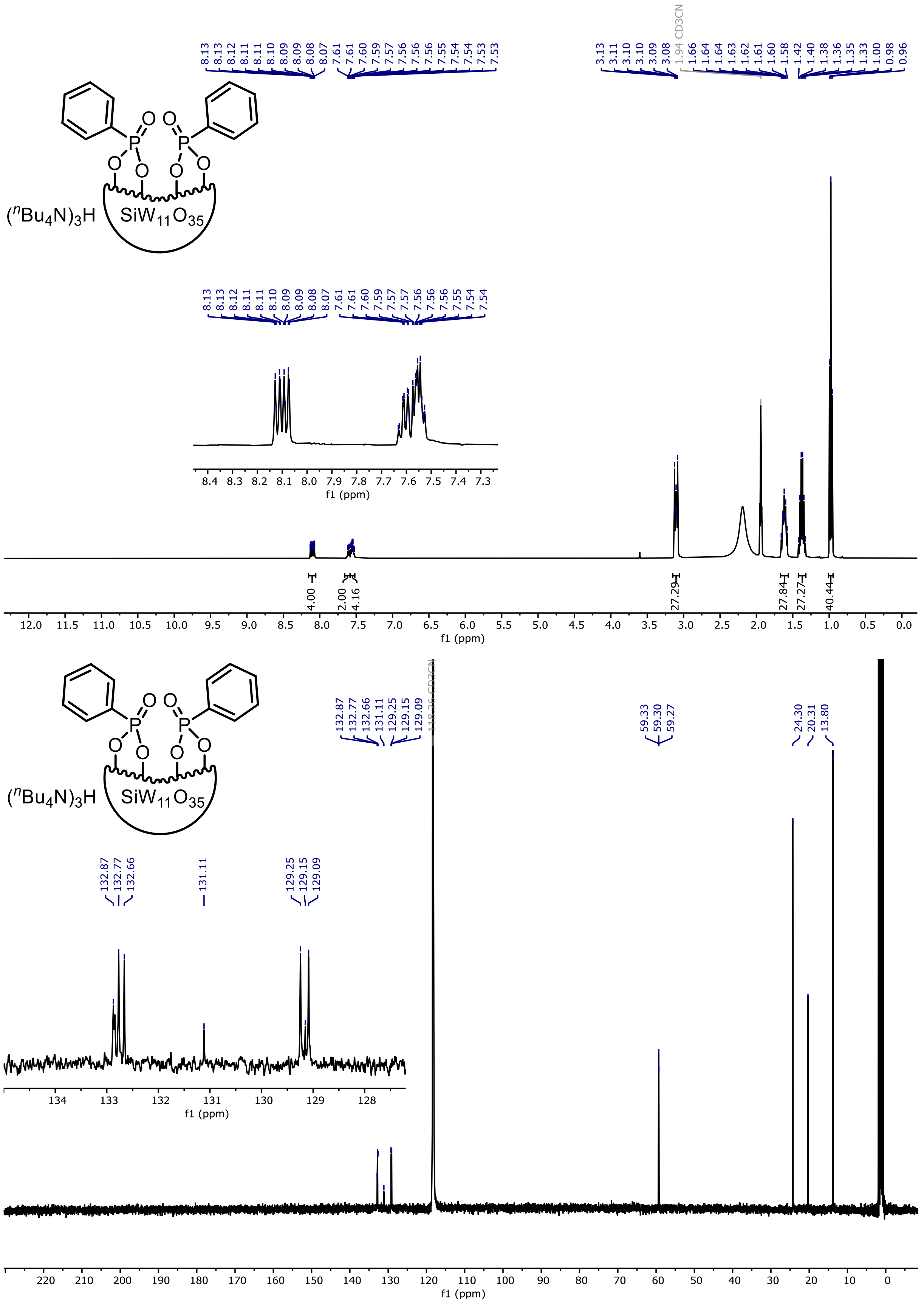

Figure S45. ^{1}H NMR (400 MHz, CD_3CN) and ^{13}C NMR (101 MHz, CD_3CN) of $(^{n}Bu_4N)_3H[SiW_{11}O_{39}(P(O)Ph)_2]$ (**$PSiW_{11}$**).

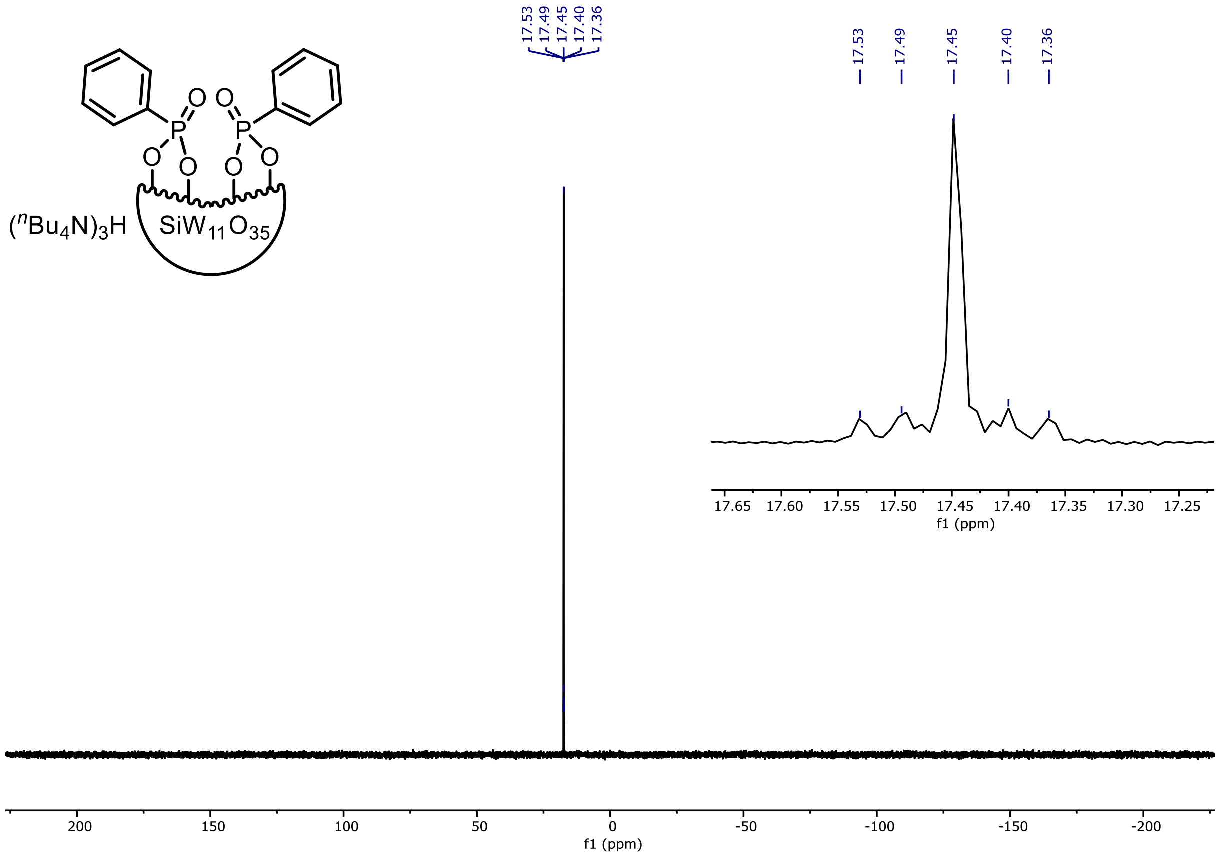

Figure S46. ^{31}P NMR (162 MHz, CD_3CN) of (^{n}Bu$_4$N)$_3$H[SiW$_{11}$O$_{39}$(P(O)Ph)$_2$] (**PSiW$_{11}$**).

7 Crystallography

$K_3H[HBW_{11}O_{39}(P(O)Ph)_2]$ (KPBW$_{11}$)

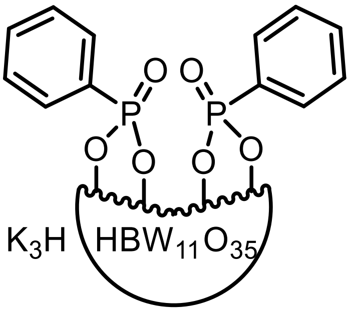

Crystals suitable for SC-XRD were prepared by vapour diffusion with nitromethane and *tert*-butyl methyl ether as the antisolvent. **CCDC identification Number: 2358933**

The structure refinement had a moderate data to parameter ratio (15.2:1), hence where chemically appropriate, restraints were applied to the anisotropic displacement parameters and geometries of the atoms (SIMU, RIGU, ISOR, SADI, SAME, FLAT, DFIX). These limitations of the data and model have resulted in lower precision in the certainty of bond distances in the structure, though atoms identities, connectivity and geometries can still confidently be inferred.

The geometries of phenyl rings C1A/B-C6A/B were restrained to be similar and symmetric (SAME). The geometries of all nitromethane solvent residues were restrained to be similar, symmetric and planar (SAME, FLAT).

Rigid bond and similarity restraints were applied to the anisotropic displacement parameters of all atoms in the structure. The anisotropic displacement parameters of atoms W1, O19, O3E, C4E and C4H were restrained to have more isotropic character (ISOR). In the case of the solvent atoms this restraint is necessary due to the high thermal motion of the atoms which might obscure underlying positional disorder for which a model could not be developed. In the case of the core polyoxometalate tungsten and oxygen atoms the need for isotropic ellipsoid restraints is likely caused by perturbations in the electron density map surrounding heavy atoms owing to deficiencies in the absorption correction process (see below).

A large number of residual peaks in the electron density map (heights 1.53-3.34 e Å^{-3}) are observed close to tungsten atoms in the polyoxometalate core (distances to tungsten atoms range 0.80-2.48 Å). For the most part these peaks are likely to be caused by deficiencies in the absorption correction process which was carried out by CrysAlisPro 1.171.43.63a empirically with a spherical correction. A face based absorption correction which would be

desirable for this heavy atom structure was not possible owing to the absence of crystal images for the synchrotron data set. A further consideration for the origin of the peaks is the presence of a minor disorder component in the polyoxometalate core (either positional disorder of the hybrid polyoxometalate species or presence of the plenary polyoxometalate): both types of disorder have been observed in similar structures. Disorder with a plenary polyoxometalate species can be ruled out given the absence of a peak for the tungsten atom that would exist in the lacunary site (approximately between the phosphorous atoms) which would be apparent in this situation. A sensible model could not be developed and refined for disorder of the main hybrid polyoxometalate core.

Hydroxy hydrogen atom H1C was refined with the O-H distance restrained to a target value of 0.95 Å (DFIX, esd 0.04 Å) and geometric similarity restraints on 1,3 distances to adjacent oxygen and tungsten atoms (SADI, esd 0.04 Å). Assignment and location of this proton are supported by spectroscopic data, the presence of proximate hydrogen bond acceptors, and reference to crystal structures of similar hybrid polyoxometalate structures (**$KAsBW_{11}$**). A hydrogen omit map was calculated for the structure (OMIT $H, OMIT 2 40 L.S. 0, PLAN −100) which indicated the presence of electron density peaks of 0.65 e $Å^{-3}$ close to hydrogen H1C and 0.46 e $Å^{-3}$ close to oxygen O1C. The presence of a comparable height electron density peak on the adjacent oxygen atom indicates that electron density map is too noisy to confirm the position of the hydroxy-hydrogen atom on the strength of the electron density map alone. All other hydrogen atoms in the structure were geometrically placed and refined with a riding model.

For further information, please see refer to data accessible free of charge from The Cambridge Crystallographic Data Centre via www.ccdc.cam.ac.uk/data_request/cif.

$K_3H[HBW_{11}O_{39}(As(O)Ph)_2]$ (KAsBW$_{11}$)

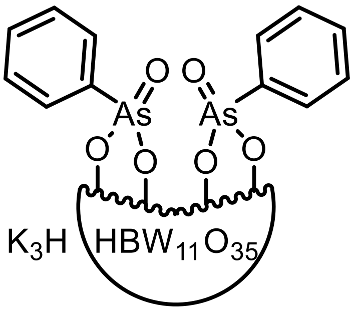

Crystals suitable for SC-XRD were prepared by vapour diffusion with nitromethane and *tert*-butyl methyl ether as the antisolvent. **CCDC identification Number: 2358934**

The crystal of a large hybrid polyoxometalate (POM) functionalised with two phenyl arsenate moieties diffracted moderately well despite containing large regions of somewhat diffuse solvent residues and potassium cations. Use of synchrotron radiation and a beamline with a large area detector allowed collection of highly redundant data (multiplicity 8.67) to a high resolution of 0.62 Å.

A minor disorder component of the plenary $BW_{12}O_{40}$ Keggin POM is modelled overlapping with the major component hybrid POM. The only unique atom modelled for this component is tungsten atom W12, modelled with an isotropic displacement parameter; the lighter boron and oxygen atoms associated with this minor disorder component are not visible in the electron density map. The occupancies of tungsten W12 from the plenary POM and the two phenyl arsonate moieties from the hybrid POM were refined and constrained to sum to unity resulting in an occupancy of 0.99(1) for the latter component.

Residual electron density peaks (range 1.75-3.77 e Å^{-3}) are located close to many of the tungsten atoms (distances to tungsten atoms range 0.55-0.68 Å). These peaks adjacent to heavy atoms in the structure are likely to be caused by deficiencies in the absorption correction process. There may also be a contribution to the peaks from unmodelled tungsten atoms from the minor disordered plenary POM component which, for the most part, is overlaid on the atoms of the hybrid POM.

Positional disorder is modelled for phenyl ring C1A/X-C6A/C6X. The occupancies of the two phenyl ring components were refined and constrained to sum to unity resulting in occupancies of 0.69(4) and 0.31(4). The geometries of the two ring components were restrained to be similar (SAME), have planar geometries (FLAT) and have identical positions and anisotropic displacement parameters for carbon atoms C1A/X where they bond to the arsenic atom (EXYZ, EADP).

Potassium cations K1A/B and K3A/B are respectively disordered over two positions. The occupancies of these disorder components are refined and constrained to sum to unity resulting in occupancies of 0.88(1) and 0.55(1) for the major component in each case. Low occupancy potassium site K1B is modelled with an isotropic displacement parameter. The occupancy of water solvent residue O1W was refined to a value of 0.81(2) and modelled with an isotropic displacement parameter. Unmodelled electron density peaks close to this site are likely to be caused by overlapping sites of positionally disordered water residues for which reliable models could not be developed and refined.

Rigid bond and similarity restraints were applied to the anisotropic displacement parameters of all disordered atoms and solvent residues (RIGU, SIMU). The anisotropic displacement parameters of nitromethane solvent residues F, G and H were restrained to have more isotropic character (ISOR).

The presence and placement of the hydroxyl hydrogen atom H1C on oxygen atom O1C is supported by both spectroscopic analysis of the compound in solution and examination of electron density maps. The position of hydrogen atom H1C is refined with the O-H distance restrained to a target value of 0.95 Å (DFIX). A hydrogen omit map calculated for all of the data (OMIT $H, L.S. 0, PLAN −200) indicated an electron density peak of 0.49 e Å^{-3} in the refined location of hydrogen atom H1C. The occupancy of hydrogen atom H1C is refined to have the same value as the hybrid POM disorder components 0.99(1). The hydrogen atoms of water residue H1W were not apparent in the electron density map and not included in the model, however they are included in the unit cell contents. All other hydrogen atoms in the structure were geometrically placed and refined with a riding model.

For further information, please see refer to data accessible free of charge from The Cambridge Crystallographic Data Centre via www.ccdc.cam.ac.uk/data_request/cif.

Table S7. Crystallographic details for compounds **$KPBW_{11}$** and **$KAsBW_{11}$**.

	**$KPBW_{11}$**	**$KAsBW_{11}$**
CCDC Deposit	2358933	2358934
Chemical formula	$C_{18}H_{29}BK_3N_6O_{53}P_2W_{11}\cdot4[H_2O]$	$0.99(C_{12}H_{11}As_2BO_{41}W_{11})\cdot 0.01(W_{12}O_{40}B)\cdot3(K)\cdot7(CH_3NO_2)H_2O$
M_r	3461.93	3555.46
Crystal system, space group	Monoclinic, $P2_1$	Monoclinic, $P2_1/n$
Temperature (K)	100	100
a, *b*, *c* (Å)	12.3035 (4), 23.9565 (5), 12.4053 (4)	12.186, 23.1617 (1), 23.1375 (1)
β(°)	118.757 (4)	97.73
V (Å^3)	3205.49 (19)	6471.15 (4)
Z (*Z`*)	2 (1)	4 (1)
Radiation type	Synchrotron, λ = 0.6889 Å	Synchrotron, λ = 0.6889 Å
μ (mm^{-1})	18.28	19.00
Crystal size (mm)	0.25 × 0.08 × 0.08 × 0.08 (radius)	0.13 × 0.05 × 0.03
Diffractometer	Fluid Film Devices	Fluid Film Devices
Absorption correction	Absorption correction For a sphere<br>CrysAlis PRO 1.171.43.63a (Rigaku Oxford Diffraction, 2023) Spherical absorption correction using equivalent radius and absorption coefficient. Empirical absorption correction using spherical harmonics, implemented in SCALE3 ABSPACK scaling algorithm.	Absorption correction Empirical (using intensity measurements)<br>CCP4 8.0.003: AIMLESS, version 0.7.9 : 10/06/22 Scaling & analysis of unmerged intensities, absorption correction using spherical harmonics
T_{min}, T_{max}	0.129, 0.167	0.935, 1.0
No. of measured, independent and observed [$I > 2\sigma(I)$] reflections	41822, 12893, 12500	282940, 32415, 27629
R_{int}	0.093	0.069
θ_{max} (°)	25.499	36.114
$(\sin \theta/\lambda)_{max}$ (Å^{-1})	0.625	0.856
$R[F^2 > 2\sigma(F^2)]$, $wR(F^2)$, S	0.050, 0.118, 1.04	0.035, 0.077, 1.08
No. of reflections	12893	32415
No. of parameters	850	957
No. of restraints	1897	933
$\Delta\rangle_{max}$, $\Delta\rangle_{min}$ (e Å^{-3})	3.33, -2.36	3.77, -2.82
Absolute structure	Flack x determined using 5237 quotients [(I+)-(I-)]/[(I+)+(I-)] (Parsons, Flack and Wagner, Acta Cryst. B69 (2013) 249-259).	-
Absolute structure parameter	0.074 (11)	-

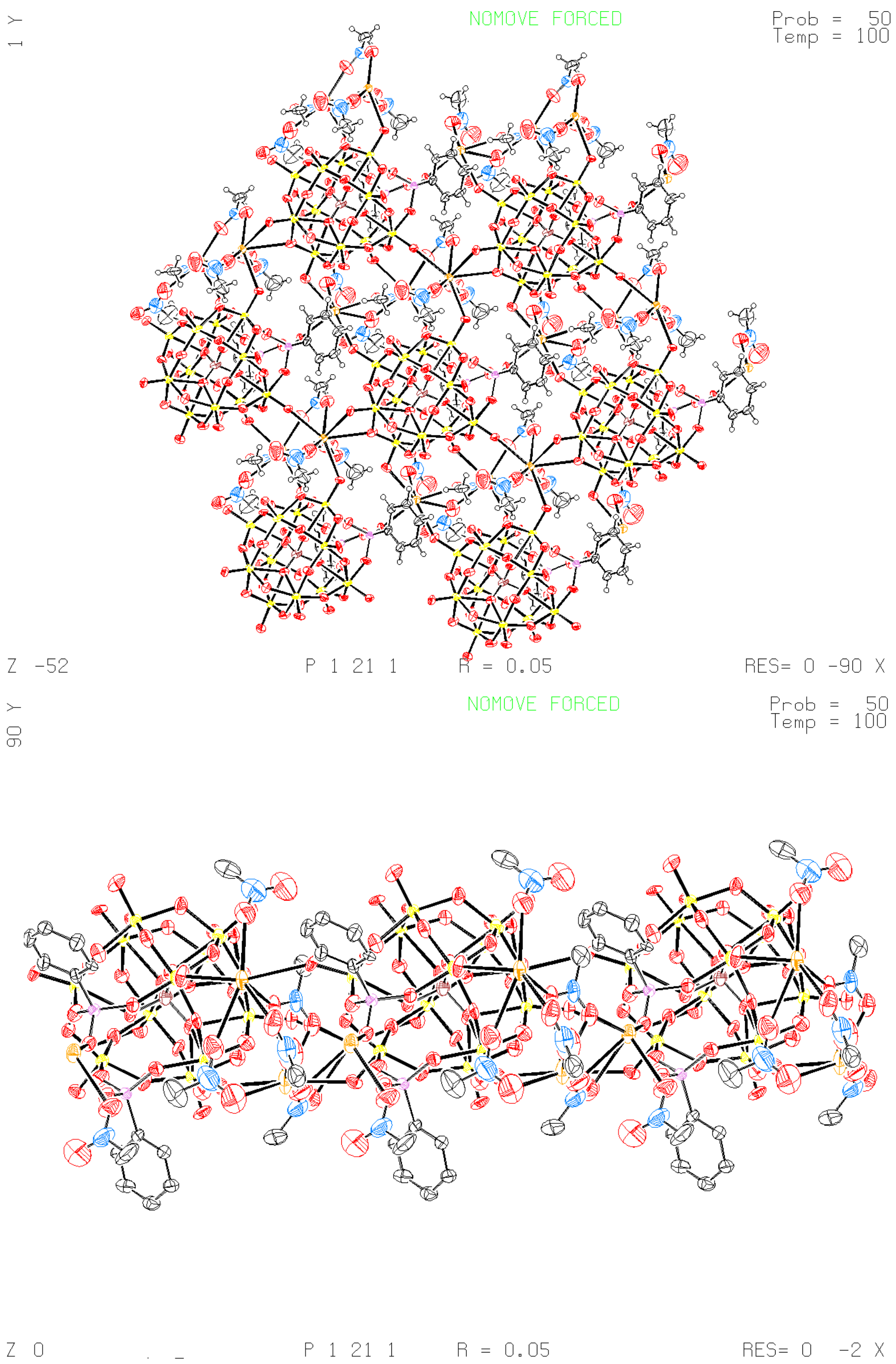

Figure S47. ORTEP diagrams of POM **KPBW$_{11}$**. *Bottom*) Hydrogen atoms omitted for clarity. Colour code: red = oxygen, yellow = tungsten, pink = phosphorus, brown = boron, black = carbon, blue = nitrogen, orange = potassium.

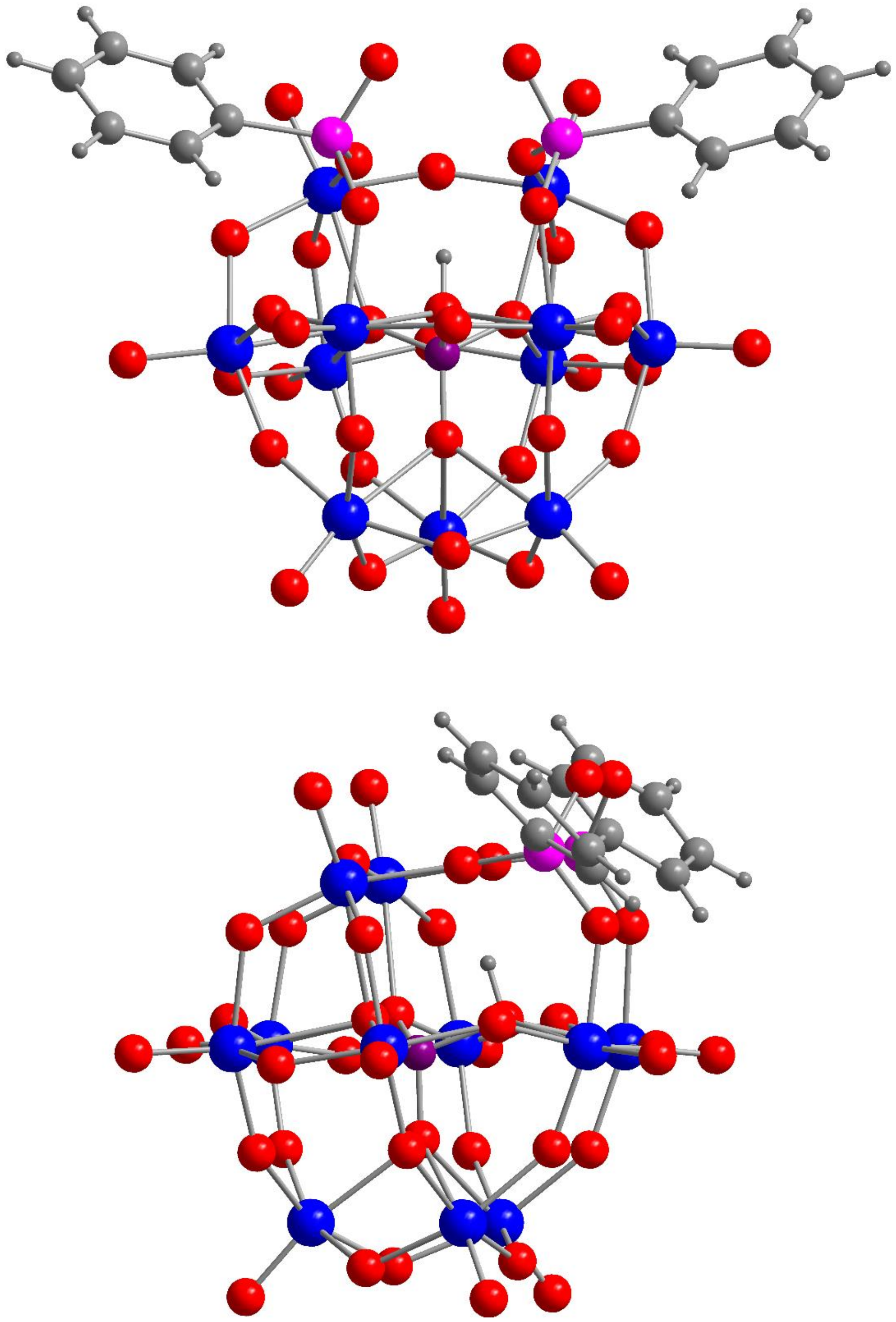

Figure S48. Z` unit of **KPBW$_{11}$** with cation and solvent omitted for clarity. Colour code: red = oxygen, dark blue = tungsten, pink = phosphorus, purple = boron, large grey spheres correspond to carbon atoms while smaller grey spheres are hydrogen atoms.

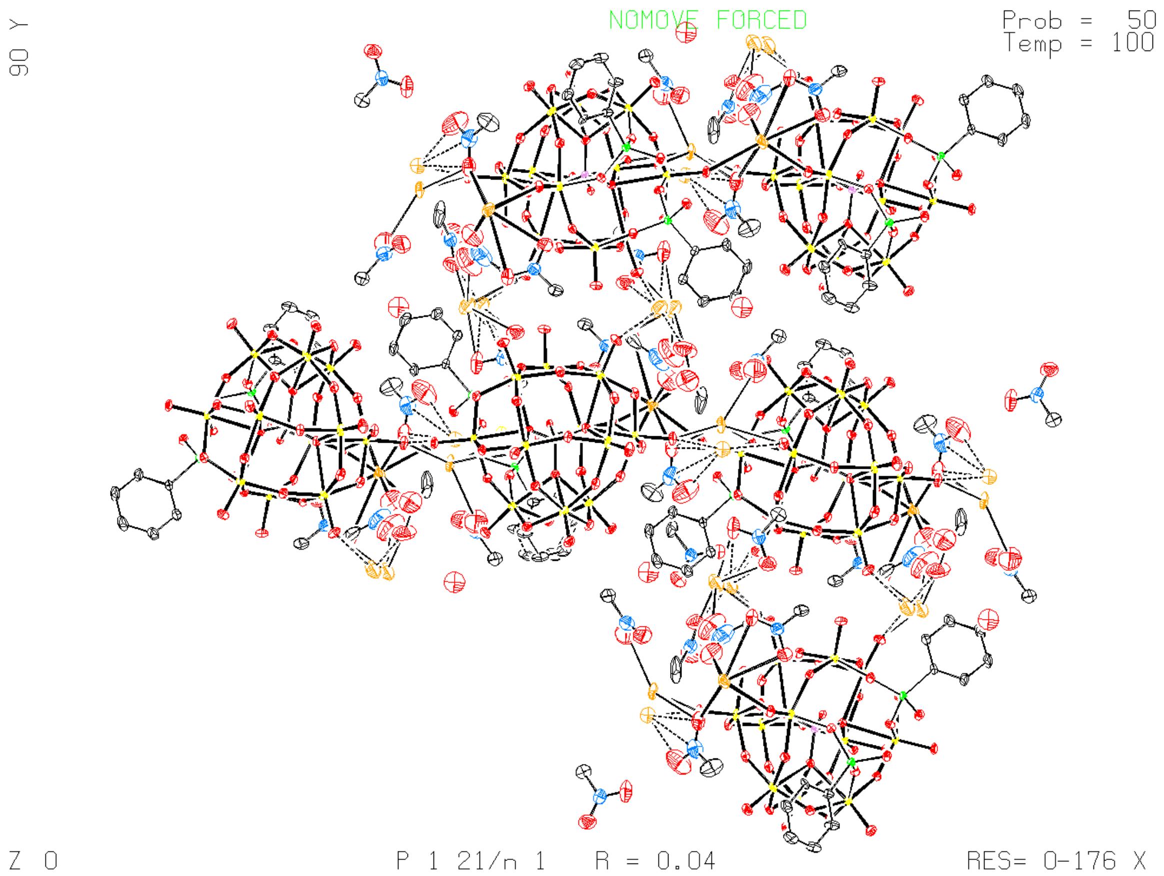

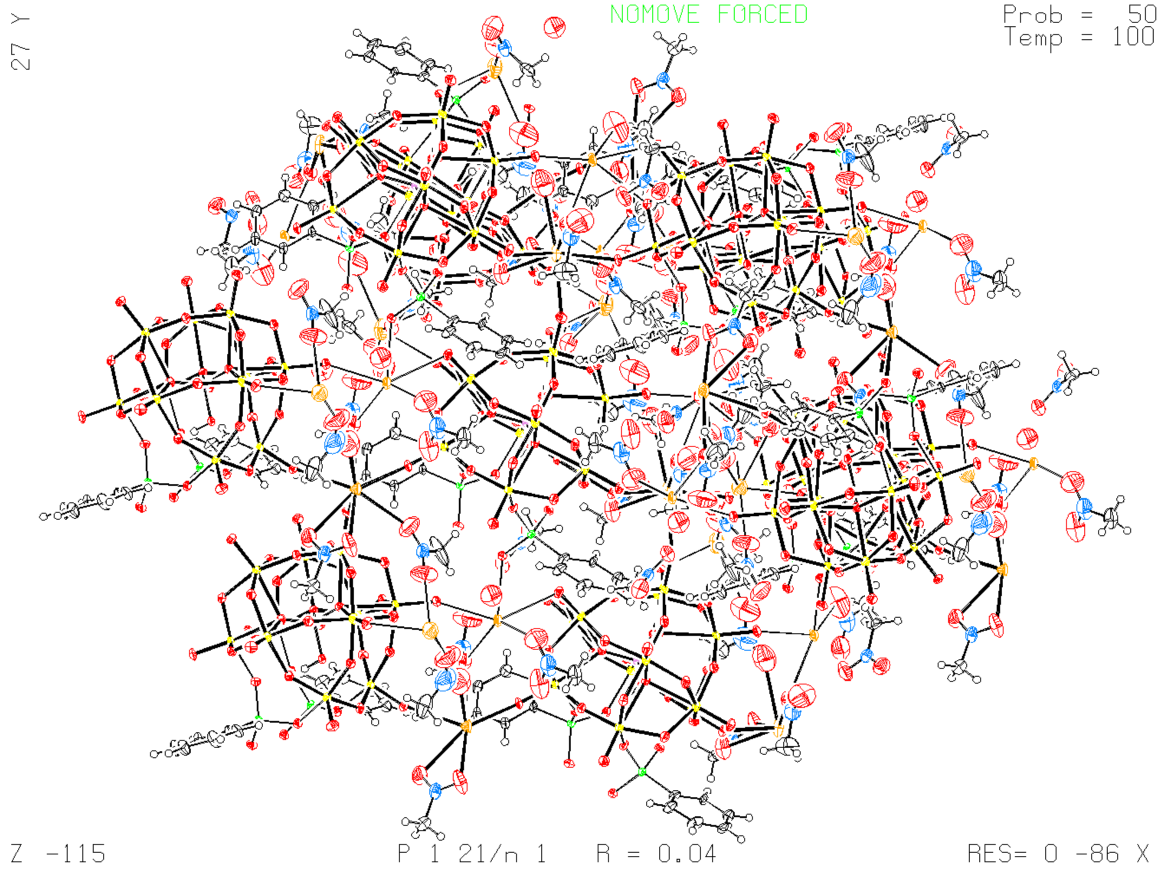

Figure S49. ORTEP diagrams of POM **KAsBW$_{11}$**. *Top*) Hydrogens omitted for clarity. *Bottom*) Disorder omitted for clarity. Colour code: red = oxygen, yellow = tungsten, green/cyan = arsenic, brown = boron, black = carbon, blue = nitrogen, orange = potassium.

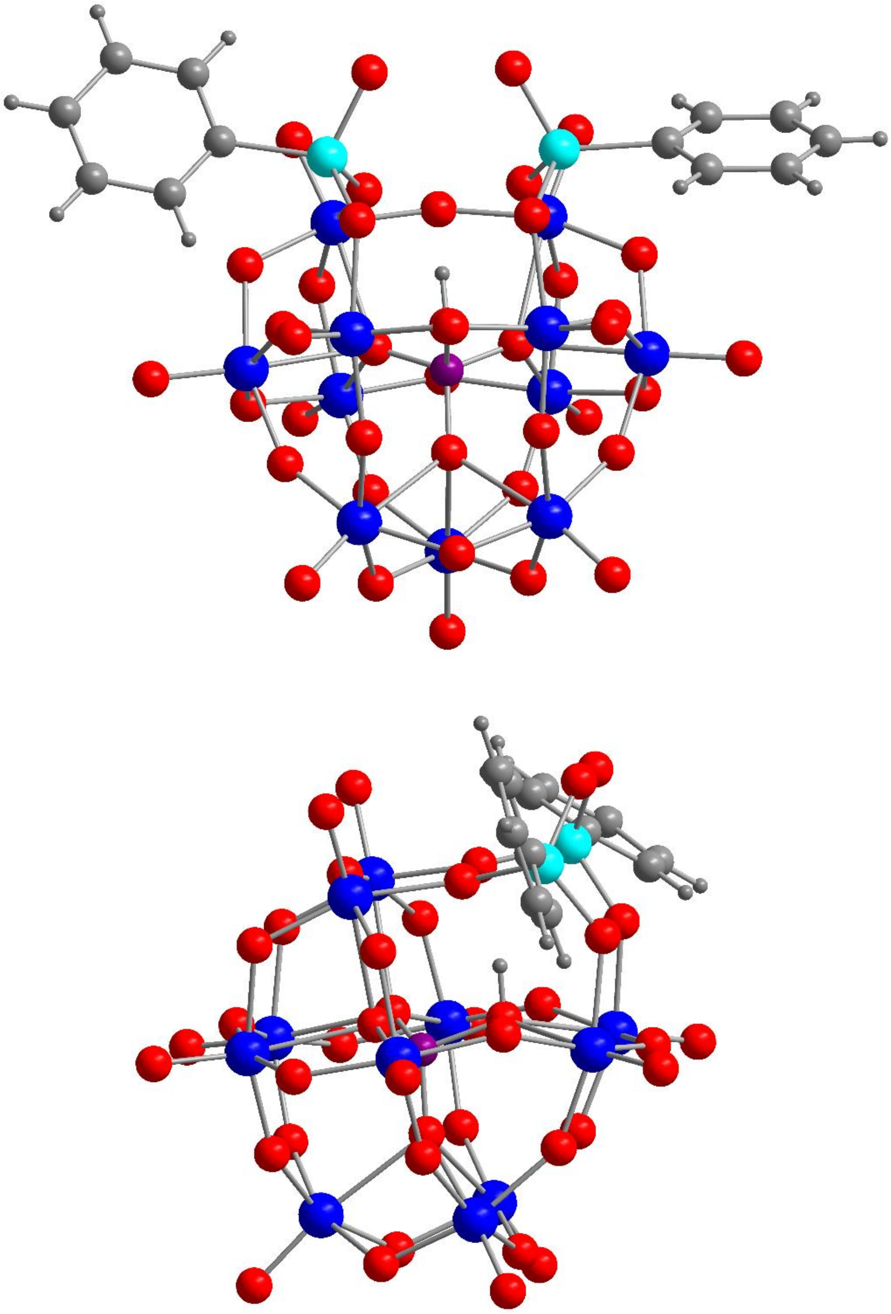

Figure S50. Z` unit of **KAsBW$_{11}$** with cation and solvent omitted for clarity. Colour code: red = oxygen, dark blue = tungsten, cyan = arsenic, purple = boron, large grey spheres correspond to carbon atoms while smaller grey spheres are hydrogen atoms.
